# The chromosome-scale reference genome of black pepper provides insight into piperine biosynthesis

**DOI:** 10.1038/s41467-019-12607-6

**Published:** 2019-10-16

**Authors:** Lisong Hu, Zhongping Xu, Maojun Wang, Rui Fan, Daojun Yuan, Baoduo Wu, Huasong Wu, Xiaowei Qin, Lin Yan, Lehe Tan, Soonliang Sim, Wen Li, Christopher A Saski, Henry Daniell, Jonathan F. Wendel, Keith Lindsey, Xianlong Zhang, Chaoyun Hao, Shuangxia Jin

**Affiliations:** 1 Spice and Beverage Research Institute, Chinese Academy of Tropical Agricultural Sciences, Wanning, Hainan 571533 China; 2 National Key Laboratory of Crop Genetic Improvement, Huazhong Agricultural University, Wuhan, Hubei 430070 China; 3 Key Laboratory of Genetic Resources Utilization of Spice and Beverage Crops, Ministry of Agriculture, Wanning, Hainan 571533 China; 4 Hainan Provincial Key Laboratory of Genetic Improvement and Quality Regulatioin for Tropical Spice and Beverage Crops, Wanning, Hainan 571533 China; 5 Academy of Sciences Malaysia, Kuala Lumpur, 50480 Malaysia; 6 Department of Plant & Environmental Science, Clemson University, Clemson, SC 29631 USA; 7 Department of Biochemistry, School of Dental Medicine, University of Pennsylvania, Philadelphia, PA 19104 USA; 8 Department of Ecology, Evolution, and Organismal Biology, Iowa State University, Ames, IA 50011 USA; 9 Department of Biosciences, Durham University, Durham, DH1 3LE UK

**Keywords:** Agricultural genetics, Phylogenomics, Plant evolution, Secondary metabolism

## Abstract

Black pepper (*Piper nigrum*), dubbed the ‘King of Spices’ and ‘Black Gold’, is one of the most widely used spices. Here, we present its reference genome assembly by integrating PacBio, 10x Chromium, BioNano DLS optical mapping, and Hi-C mapping technologies. The 761.2 Mb sequences (45 scaffolds with an N50 of 29.8 Mb) are assembled into 26 pseudochromosomes. A phylogenomic analysis of representative plant genomes places magnoliids as sister to the monocots-eudicots clade and indicates that black pepper has diverged from the shared Laurales-Magnoliales lineage approximately 180 million years ago. Comparative genomic analyses reveal specific gene expansions in the glycosyltransferase, cytochrome P450, shikimate hydroxycinnamoyl transferase, lysine decarboxylase, and acyltransferase gene families. Comparative transcriptomic analyses disclose berry-specific upregulated expression in representative genes in each of these gene families. These data provide an evolutionary perspective and shed light on the metabolic processes relevant to the molecular basis of species-specific piperine biosynthesis.

## Abstract (web summary)

Black pepper (*Piper nigrum*) belongs to the long-isolated lineage of basal angiosperm and its fruit has been used for food spice and phytomedicines for thousands of years. Here, the authors assemble the reference genome of this species and analyze gene families associated with piperine biosynthesis.

## Introduction

Black pepper (*Piper nigrum*, 2n = 52), known as the ‘King of Spices’, is one of the oldest and most widely used seasonings in the world. It had been highly valued and considered as ‘Black Gold’, occupying a preeminent status in the spice trade. It was the primary spice in early trading between Europe and Asia. The production, transportation and consumption of black pepper has influenced the destinies of nations and their people, both economically and culturally^1^. Originating in the humid, tropical evergreen forests of the Western Ghats of South India, black pepper is now cultivated in most tropical and subtropical regions, with production primarily in Vietnam, Indonesia, Brazil, India, Sri Lanka, China, Malaysia and Cambodia. In 2017, the world’s total cultivation area was 458,731 hectares, which gives 510,045 metric tons of production and 2 billion US dollar trade value. It is also an important cash crop for small farmers in many developing countries.

Black pepper is a perennial, woody climbing vine. It belongs to the family Piperaceae, which is the largest family in the order Piperales. In current phylogenetic classification^2–5^, Piperales is considered as a sister order of the Canellales, Laurales and Magnoliales orders within the Magnoliid clade. However, the phylogenetic position of magnoliids relative to eudicots and monocots is still unsettled, even based on the two newly published magnoliid genomes, which indicates a controversial phylogenetic understanding of these long-isolated lineages^6–8^.

The Piperales are well known for their special phytochemistry, particularly their unique piperidine alkaloids. Piperine is the major alkaloid responsible for the pungency and flavour of black pepper. In addition to being a common culinary spice and a preservative for meat products, black pepper has been widely used in traditional medicinal systems, such as the Indian Ayurvedic system, traditional Chinese medicine and folklore medicines of Latin America and Southeast Asia^9^. Piperine possesses a range of pharmacological activities, including the attenuation of fat cell differentiation through the downregulation of peroxisome proliferator-activated receptor (PPAR) gamma expression, leading to its use for treatment of diabetes as a PPAR agonist^10^. It has also been used as an antioxidant, antitumour, antimicrobial, anti-depressive and anti-inflammatory^11^ agent. The phytochemical and pharmacological characteristics of black pepper have received renewed attention in recent decades. However, relatively little is understood about the genetic mechanisms controlling of its biosynthetic pathway and accumulation, and few genetic resources are available for black pepper.

Here, we report a reference genome of black pepper using a combination of four technologies. Evolutionary analysis of three available magnoliid genomes from different orders provides evidence for the phylogenetic position of the magnoliids. In addition, our comparative genome and transcriptome analyses identify changes in gene expression, evolution and family size associated with piperine biosynthesis. Genomic resources provided here will be valuable for biological and agronomic research in *Piper* species.

## Results

### Genome assembly and main features

Cv. Reyin1 derived from the cultivar ‘Lampung Daun Kecil’ was used for genome sequencing. Based on the k-mer genome survey analysis (Supplementary Fig. 1), black pepper (Supplementary Note 1) was estimated to have a genome size of 761.74 Mb. K-mer analysis with a length of 17 indicates the genome had high heterozygosity (1.33%) and a repetitive sequence content of 59.54% (Supplementary Table 1).

To overcome the impact of heterozygosity and repetitive sequence content on the construction of a chromosome-scale reference genome, a comprehensive de novo assembly strategy (Supplementary Fig. 2) combining Illumina paired-end reads (137 × coverage), PacBio single-molecule long reads (N50 length of 13 Kb, ~138 × coverage), 10X Genomics, BioNano (Supplementary Table 2), and Hi-C sequencing (Supplementary Table 3) was adopted. The workflow is summarised as follows: (1) FALCON^12^ was selected for the PacBio-only assembly, using the overlap-layout-consensus algorithm and FALCON-Unzip^12^ for true diploid assembly; (2) fragScaff^13^, which leverages information derived from different barcoded pools, was used to order and orient linked contigs into longer scaffolds, which resulted in an assembly (Piper_nigrum_v1) consisting of 1277 scaffolds with an N50 of 2.3 Mb and a total length of 791.0 Mb; (3) a non-haplotype-aware hybrid assembly with ‘no extend split’ and ‘no cut segdups’ parameters (according to BioNano’s suggestions) was performed using BioNano Solve tools, yielding an assembly with a total molecule length of 316,350.85 Mb and 128 × effective coverage (Supplementary Table 2). This improved version (Piper_nigrum_v2) contains 201 hybrid scaffolds with an N50 of 7.8 Mb (3.4 fold improvement compared with the Piper_nigrum_v1) and a longest scaffold of 25.8 Mb (Supplementary Figs. 3–5); (4) additional scaffold refinement was performed in a Hi-C experiment with ~125 million unique Di-Tags read pairs (Supplementary Table 3 and Supplementary Fig. 6) and postprocessing by gap filling and polishing to generate the final version of the assembly, ‘Piper_nigrum_v3’. This final assembly contains only 45 scaffolds, with a scaffold N50 of 29.8 Mb and 99.9% of the assembled genome contained in 26 scaffolds (Table 1). Inasmuch as the chromosome number of *Piper nigrum* is 2n = 52^14^, we infer that these large scaffolds reflect a chromosome-scale assembly (Supplementary Figs. 7–14). The assembly size of 761.22 Mb (99.93% coverage of the genome) was very similar to the estimated genome size of 761.74 Mb obtained from the k-mer analysis. The higher heterozygosity estimated from the k-mer analysis was also consistent with single-nucleotide polymorphism (SNP) calling in the final reference genome (Supplementary Note 4 and Supplementary Fig. 15).

**Table 1:** Major indicators of the *Piper nigrum* genome

Assembly feature	Statistic
Estimated genome size (by k-mer analysis) (Mb)	761.74
Number of scaffolds	45
Scaffold N50 (Mb)	29.8
Longest scaffold (Mb)	48.45
Assembled genome size (Mb)	761.22
Assembly % of genome	99.93
Repeat region % of assembly	54.85%
Predicted gene models	63,466
Average coding sequence length (bp)	1347
Average exons per gene	5.84

Detailed assembly data are summarised in Table 1. The extent of comprehensive gene coverage was assessed by screening for 248 core eukaryotic genes (CEGs)^15^, which revealed a complete and partial matches for 234 (94.35%) and 244 (98.39%) genes, respectively (Supplementary Table 4). BUSCO^16^ analysis against the plant-specific database containing a total of 430 genes revealed 413 (96.1%) complete BUSCOs, 80 (19.1%) of which were duplicated genes (Supplementary Table 5). These data support the interpretation that the *P. nigrum* genome assembly is reasonably complete.

**Fig. 1:**
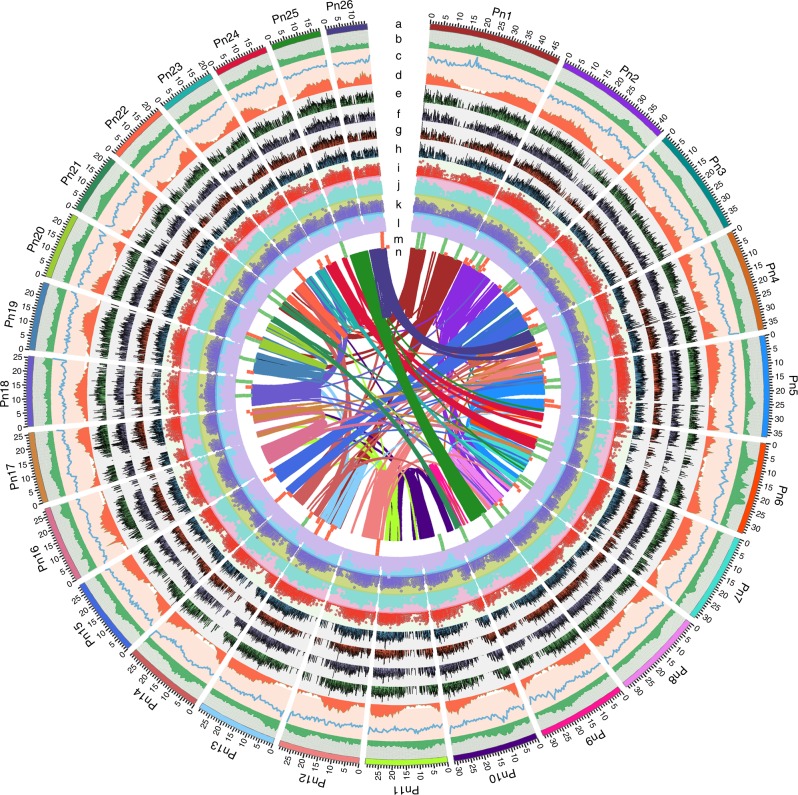
Black pepper genomic landscape of diversity and expression data. **a** Circular representation of the pseudomolecules. **b**–**d** The distribution of the GC density, repeat density and gene density, respectively, with densities calculated in 500 Kb windows. **e**–**l** Expression of berry-specific genes (from outside to inside tracks: 2 MAP, 4 MAP, 6 MAP, 8 MAP, root, stem, leaf and flower). **m** Locations of genes mapped to secondary metabolism (green square) and alkaloid metabolism (red square) pathways. **n** Syntenic blocks. The band width is proportional to syntenic block size. Source data are provided as a Source Data file

A total of 54.85% repetitive sequences were identified in the black pepper genome. Among these repeats, 54.01% are classified as interspersed repeats (Supplementary Table 7). Similar to most plant genomes^17^, the predominant type of transposable elements (TEs) was long terminal repeat (LTR) retrotransposons, accounting for 40.55% of the genome, including 27.63% LTR/Gypsy and 9.95% LTR/Copia retro-elements (Supplementary Note 2, Supplementary Tables 8–9 and Supplementary Fig. 16). These TEs exhibit an apparently random distribution on the chromosomes, and an inverse correlation with gene density (Fig. 1; Supplementary Figs. 17–25).

We conducted Illumina strand-specific RNA sequencing (RNA-seq) using eight different tissues and organs, and performed PacBio isoform sequencing (Iso-Seq) (Supplementary Fig. 26) to provide transcriptional evidence supporting the annotation and to obtain reliable gene structure annotation. We also employed a strategy that combined ab initio and evidence-based gene prediction using the BRAKER2^18^ pipeline. The black pepper genome encodes 63,466 inferred protein-coding genes, with an average length of 900 bp and an average GC content of 51.21%. Annotation Edit Distance (AED)^19^ quantification showed a high AED of 0.04 at the nucleotide-level and 0.13 at the exon level, indicating a highly credible gene model.

Five thousand eighty-two transcription factors (TFs) from 75 gene families accounting for 8.0% of the protein-coding genes are categorised in this report. In addition, 646 chromatin regulators (CRs), 157 transcriptional regulators (TRs), 6509 long non-coding RNAs (lncRNAs), 1514 tRNAs, 1206 rRNAs, 1533 small nuclear RNAs (snRNAs) and 256 microRNAs (miRNAs) were also identified (Supplementary Note 3 and Supplementary Fig. 27). An InterProScan Pfam analysis identified 3652 protein families containing 21,184 proteins and 2071 Gene Ontology (GO) terms, of which 41.63%, 13.19% and 45.18% of the genes were annotated in the biological process, cellular component and molecular function categories, respectively (Supplementary Fig. 28).

### Comparative genomic and phylogenomic analyses

**Fig. 2:**
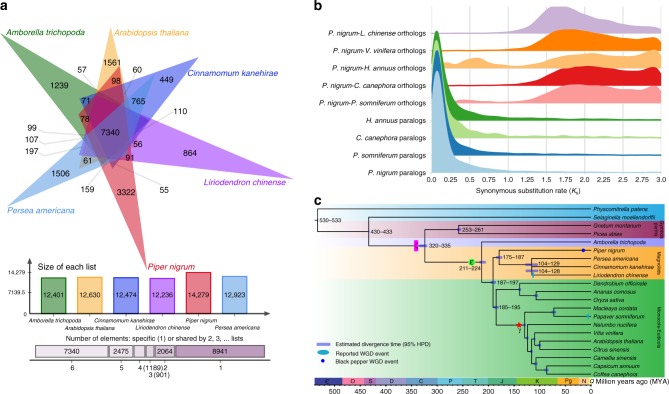
Comparative genomic analysis of black pepper and WGD. **a** Sharing of gene families by black pepper and five other species. The numbers indicate gene families identified among all selected species. **b** Synonymous substitution rate (*K*_s_) distributions of syntenic blocks for *Piper nigrum* paralogs and orthologs with other eudicots are shown by coloured lines, as indicated. **c** Phylogenetic tree with 82 single-copy orthologs from 21 species identified by OrthoMCL to show divergence times. *Piper nigrum* was placed sister to Magnoliales–Laurales among the magnoliids. Posterior probabilities for all branches exceed 0.99. Divergence times were estimated using BEAST and are indicated by light blue bars at the internodes with 95% highest posterior density (HPD). Є Cambrian, O Ordovician, S Silurian, D Devonian, C Carboniferous, P Permian, T Triassic, J Jurassic, K Cretaceous, Pg Paleogene, N Neogene. The source data underlying Fig. 2a and b are provided as a Source Data file

Ancient whole-genome duplication (WGD) (also known as polyploidization) events are important driving forces of the evolution of animals, fungi and other organisms, particularly plant lineages^20,21^. We selected a range of species to perform a comparative genomic investigation and assess WGD in black pepper: *Papaver somniferum* (WGD ~7.8 million years ago (MYA))^22^; *Liriodendron chinense* as a representative of the Magnoliidae, with a WGD event ~116 MYA^6^; *Coffea canephora* as a representative of whole-genome triplication (WGT) of the eudicots (WGT-γ) without WGT-1 and WGT-2^23^; *Helianthus annuus* for its lineage-specific WGD-2 with shared ancestral WGT-γ and WGT-1^24^; and *Vitis vinifera* which represents the closest modern chromosome relative of the ancestral eudicot karyotype (AEK) with seven protochromosomes^25,26^. The reciprocal best hit (RBH) gene pair synonymous substitution rate (*K*_s_) distribution (Supplementary Fig. 29) recovered the WGT-γ in *Coffea canephora* and a relatively recent WGD event in *Helianthus annuus* and *Papaver somniferum*, consistent with the findings of previous reports^24,27,28^. Indeed, the all-vs-all paralog analysis in black pepper genome detected 31,138 RBH paralogous gene pairs and the RBH paralog *K*_s_ distribution showed a single peak at ~ 0.1 (Fig. 2b; Supplementary Fig. 29a). Second, the synteny dot plot analysis revealed duplications within the black pepper genome that are either inter-chromosomal or intra-chromosomal duplications (Supplementary Fig. 30).

An analysis of the genomic synteny of black pepper using MCScanX revealed 1295 syntenic blocks across the whole genome including 28,621 genes that accounted for 45.10% of the total number of genes. Among these syntenic blocks, 855 (66.0%) of the paralogous gene pairs were located inter-chromosomally, and the other 440 (34.0%) were located within chromosomes (Fig. 1). In addition, an analysis of the type of duplication of the black pepper paralogs using MCScanX indicated that most genes were classified as WGDs or segmental duplications (32,547 genes, accounting for 51.3%), followed by three other types: dispersed (19.1%), proximal (7.4%) and tandem (3.6%) duplications. We also performed a comparative genomic analysis of black pepper with *Amborella* and *Cinnamomum kanehirae* and identified 1:1 and 1:2 syntenic depth ratios in the *Amborella*-*Cinnamomum kanehirae* and *Amborella*-*Piper nigrum* comparisons (Supplementary Fig. 31a), respectively. Furthermore, an analysis of synteny in black pepper with *Amborella* showed 316 syntenic blocks that covered 40% and 34% of the assembled genomes, respectively (Supplementary Fig. 31b and Supplementary Table 10). By calculating the *K*_s_ of the black pepper syntenic block gene pairs, a major peak was detected at ~0.1 (Supplementary Figs. 29b, 32).

The RBH and syntenic block gene pair *K*_s_ distribution (Supplementary Note 4) provided convincing evidence for a WGD event during black pepper genome evolution. Based on the slow substitution rate of basal angiosperms, we speculate that the black pepper WGD event (*K*_s_ = 0.106 ± 0.002) occurred ~17.2–17.9 MYA with a synonymous substitution rate of 3.02E−9 synonymous substitutions per year^29^ (Fig. 2c).

The high-quality reference genome for black pepper enabled us to perform comparative genomics among relatively early diverging angiosperms. We compared the black pepper genome with the genomes of nine eudicots, three monocots, three magnoliids, *Amborella*, two gymnosperms and two species *Selaginella moellendorffii* and *Physcomitrella patens* as the outgroups.

Eighty-two single-copy orthologous gene families among the 21 species were identified using OrthoMCL to accomplish this goal, and a phylogenetic analysis of these families using BEAST placed magnoliids as a sister clade to the monocots–eudicots clade, consistent with *Liriodendron* genome research^6^, the Plastid Phylogenomic Angiosperm (PPA) tree^30^ and Angiosperm Phylogeny Group (APG) IV^5^. Furthermore, *Piper nigrum* was placed as a sister to Magnoliales–Laurales among the magnoliids (Fig. 2c), consistent with its phylogenetic position inferred from chloroplast genomes^31^. Based on our results, Piperales, representated by *Piper nigrum*, first diverged from the Magnoliales (*Liriodendron chinense*) plus Laurales (*Cinnamomum kanehirae* and *Persea americana*) approximately 175–187 MYA (95% highest posterior density (HPD) interval).

### Evolution piperine biosynthesis-associated genes

Piperine is synthesised from two direct precursor substrates, piperoyl-coenzyme A and piperidine, in a reaction catalysed by an acyltransferase^32^. Thus, piperine production is associated with three major gene groups (see below): group I: genes in the phenylpropanoid pathway (KEGG pathway: map00940), which produce cinnamoyl-CoA for piperoyl-coenzyme A biosynthesis via a few complex reactions, such as amino transfer and elimination of ammonia-lyase and cinnamate 4-hydroxylation; group II: genes involved in L-lysine metabolism (KEGG pathway: map01064), which catalyse the transformation of lysine to piperidine via a series of reactions including decarboxylation, amine oxidation, cyclization and reduction; and group III: acyltransferase genes, that catalyse the synthesis of piperine in the presence of piperoyl-coenzyme A and piperidine.

We acquired insights into the genomic basis of piperine biosynthesis, using OrthoMCL to identify orthologous genes and CAFE to identify gene family clusters, with a specific focus on the expansion (gain) and contraction (loss) events related to the above three gene groups in black pepper. This process identified 39,400 gene families consisting of 471,854 genes among the 21 species (Fig. 2a; for clarity, only species with a close evolutionary relationship to black pepper are shown). Four hundred twenty-three gene families containing 4228 genes were unique to black pepper.

**Fig. 3:**
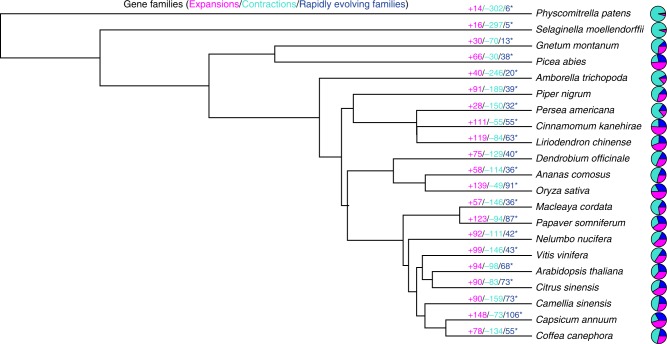
Estimation of gene family expansion and contraction on each evolutionary branch. Numbers over the branches indicate the number of expansions and contractions in gene families. Magenta indicates the number of expansions, turquoise indicates the number of contractions and blue indicates the number of significantly (*p*-value ≤ 0.01) expanded and contracted gene families. The pie charts on the right show the proportions of these categories. Source data are provided as a Source Data file

**Fig. 4:**
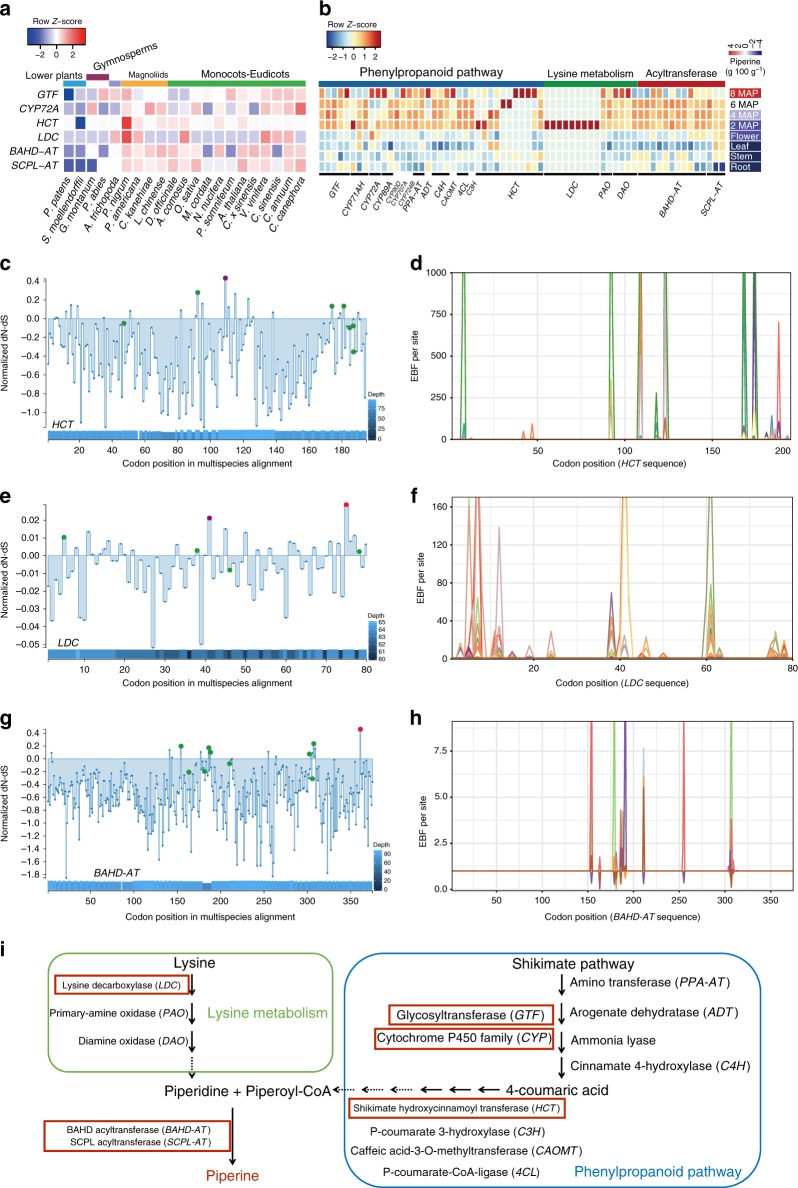
Analysis of gene families involved in black pepper piperine metabolism. **a** Expansion of genes involved in the phenylpropanoid pathway, lysine metabolism and acyltransferase family in black pepper. **b** Tissue-specific upregulated expression of genes involved in the phenylpropanoid pathway, lysine metabolism and acyltransferases in different tissues. The heatmap on the right shows the piperine content in the freeze-dried fresh tissues. MAP: months after pollination. **c**–**h** Selection analysis of *HCT*, *LDC* and *BAHD-AT* gene expansion events. Red points indicate positive selection. Green points indicate episodic selection. Purple points indicate episodic positive selection. Blue bars show sequencing depth. A significance threshold of α = 0.1 was used for both SLAC and MEME. **c** Normalised dN-dS (SLAC) values across a multispecies alignment of 94 *HCT* sequences with 195 sites. Points indicate statistically significant evidence for codons under selection. One site shows positive selection across the entire tree (SLAC); eight sites show episodic selection (MEME). **d** Comparison of episodic selection on particular codons across black pepper *HCT* genes (*n* = 69). **e** Normalised dN-dS (SLAC) values across a multispecies alignment of 72 *LDC* sequences with 80 sites. Two sites show positive selection across the entire tree (SLAC); five sites show episodic selection (MEME). **f** Comparison of episodic selection on particular codons across black pepper *LDC* genes (*n* = 57). **g** Normalised dN-dS (SLAC) values across a multispecies alignment of 87 *BAHD-AT* sequences with 375 sites. One site shows positive selection across the entire tree (SLAC); nine sites show episodic selection (MEME). **h** Comparison of episodic selection on particular codons across black pepper *BAHD-AT* genes (*n* = 6). **i** Schematic representation of the phenylpropanoid pathway and lysine metabolism branch with the reactions associated with piperine biosynthesis genes. The solid lines indicate genes catalysing major reactions that were characterised in our study. The dotted lines indicate genes catalysing reduction reactions in lysine metabolism and approximate derivative reactions in the phenylpropanoid pathway that were not characterised in our study. The source data underlying Fig. 4a–h are provided as a Source Data file

According to the CAFE analysis, 91 gene clusters (including 21,368 genes) and 189 gene clusters (including 14,201 genes) expanded and contracted, respectively, in the black pepper genome. Among these clusters, 35 and 4 families exhibited significant (family-wide *p*-value ≤ 0.01) contractions and expansions, respectively (Fig. 3). Furthermore, the species-specific expanded genes were significantly enriched in two main types, one of which was genes with secondary metabolite-associated functions, such as glycosyltransferases (*GTFs*; 19 genes), shikimate hydroxycinnamoyl transferases (*HCTs*; 69 genes), cytochrome P450s (*CYP*; 31 genes), lysine decarboxylases (*LDCs*; 75 genes), BAHD acyltransferases (*BAHD-ATs*; 6 genes) and serine carboxypeptidase-like acyltransferases (*SCPL-ATs*; 7 genes) (Fig. 4a; Supplementary Fig. 33). These expanded genes occurred in gene clusters on some chromosomes (Supplementary Fig. 34a–f). A KEGG pathway analysis of these specific gene families revealed marked enrichment in genes involved in piperine synthesis, including the biosynthesis of alkaloids from the shikimate pathway (map01063); alkaloids from ornithine, lysine and nicotinic acid (map01064); phenylpropanoid biosynthesis (map00940); and phenylalanine, tyrosine and tryptophan biosynthesis (map00400). The other category was disease resistance-related genes, such as disease-resistance proteins (RPS5, RPP13, RGA1, RGA2, RGA3, RGA4 and RGA5), NBS-LRR disease-resistance proteins, LRR receptor-like serine/threonine protein kinases (EFR, BAM1, FLS2, GSO2 and EMS1) (Supplementary Fig. 34 g,  h) and salicylic acid-binding protein 2. These genes have been widely reported to play important roles in pathogen-resistance mechanisms. The expansion of these genes might indicate that biotic stresses from pathogen infection and animal ingestion are a source of major selection pressure on black pepper in the tropical rainforest.

### Upregulation of piperine biosynthesis-related genes

The piperine content determined using high-performance liquid chromatography (HPLC) (Supplementary Note 5) revealed that the organ with the highest content was berry at 8 months after pollination (MAP), followed by 6 MAP, 4 MAP, 2 MAP, flower, root, stem and leaf (Fig. 4b). A transcriptomic analysis was performed with RNA-seq using samples from different organs of black pepper, including berries (which synthesise piperine) at different developmental stages (2 MAP, 4 MAP, 6 MAP and 8 MAP; Supplementary Fig. 35), and organs not containing piperine, i.e., the stem, root, leaf and flower (Supplementary Note 6 and Supplementary Figs. 36–38), to investigate genes involved in piperine production. A gene set enrichment analysis (GSEA)^33^ and weighted gene co-expression network analysis (WGCNA)^34^ revealed that upregulated genes in berries relative to nonberry organs were associated with the phenylpropanoid pathway (Supplementary Fig. 39) and lysine metabolism. The transcriptional module (Supplementary Figs. 40–42) of piperine synthesis in berries exhibited significant enrichment of the amino transferase (*PPA-AT*), arogenate dehydratase (*ADT*), cinnamate 4-hydroxylase (*C4H*/*CYP73*^35,36^), *HCT*, *p*-coumarate 3-hydroxylase (*C3H*), caffeic acid-3-O-methyltransferase (*CAOMT*), *p*-coumarate-CoA-ligase (*4CL*), *GTF, CYP72* and *CYP71* clans (Fig. 4b; Supplementary Fig. 43). These genes are involved in the metabolism of phenylpropanoids to produce piperoyl-CoA^37^ (Fig. 4i). In addition, the differential expression of *LDC*, primary-amine oxidase (*PAO*) and diamine oxidase (*DAO*) in black pepper berries (Fig. 4b) is consistent with the hypothesis that lysine, rather than quinolizidine or indolizidine, is the intermediate precursor of piperidine synthesis in black pepper. Importantly, the expansion of the *BAHD-AT* and *SCPL-AT* gene families (11 *BAHD-ATs* and 3 *SCPL-ATs*) (Fig. 4a), accompanied by the high transcriptional activity of these genes in berries (Fig. 4b), is associated with the metabolism of phenylpropanoids and lysine into piperine (Fig. 4i).

### Purifying selection of piperine biosynthesis gene families

Based on our analysis of gene expansion and expression, we synthetically investigated the gene-sequence-level features of the aforementioned three gene groups exhibiting expansion and berry-specific upregulated expression that might contribute to the unique piperine production in black pepper.

Within group I, the *GTF*, *CYP* and *HCT* gene families were significantly expanded in black pepper (Fig. 4a). *HCT* has been reported to play a crucial role in piperoyl-CoA synthesis (a precursor for piperine biosynthesis) by catalysing the transformation of *p*-coumaroyl-CoA into *p*-coumaroyl shikimic acid and caffeoyl shikimic acid into caffeoyl-CoA^38^. The *HCT* gene family was expanded in the black pepper genome (69 genes in black pepper compared with one or two genes in the other species). The particularly high expression in berries (2 MAP, 6 MAP and 8 MAP; Fig. 4b) detected by analysing transcriptomes from different organs is consistent with a role for this family in the biosynthesis of piperine. In the sequence-level analysis of *HCT* expansion, most conserved regions (107 sites) were under strong purifying selection, with one site showing diversifying selection and eight sites showing episodic selection (Fig. 4c, d). The adaptive expansion of *HCT* and the maintenance of duplicates appear to have operated in concert, resulting in higher enzyme levels for the accumulation of the necessary donor precursor used for piperine biosynthesis (Fig. 4i). Despite the significant expansion of the *GFT* and *CYP* gene families in black pepper, no positively selected sites were detected (Supplementary Fig. 44a, b). The significant expansion of *GFT*, *CYP* and *HCT* family genes suggests the importance of the phenylpropanoid pathway, which provides precursors for the biosynthesis of alkaloids and flavonoids typical of modern pepper cultivars (Fig. 4i).

Within group II, LDC catalyses the first step in lysine metabolism (Fig. 4i) and serves as the rate-limiting enzyme in the synthesis of lysine-derived alkaloids, including piperidine alkaloids (piperine), quinolizidine alkaloids and indolizidine alkaloids^39^. Seventy-five *LDC* genes were detected in black pepper, compared with just six in *Ananas comosus*, five in *Vitis vinifera* and at most one in the other species studied (Fig. 4a). An analysis of sequence evolution in the *LDC* family indicated that 19 sites have been under strong purifying selection, two sites have been under diversifying selection and five sites under episodic selection (Fig. 4e, f). The expansion of *LDC* genes suggests the unique activation of lysine-derived alkaloid synthesis in black pepper.

Within group III, *BAHD-AT* and *SCPL-AT* encode the two main acyltransferase families that use phenolic compounds as donor molecules^40^ (Fig. 4i). The *BAHD-AT* and *SCPL-AT* genes were expanded to include six and seven members, respectively, in black pepper. An assessment of sequence evolution revealed that 261 sites have been under strong purifying selection, one site has been under diversifying selection and nine sites under episodic selection in *BAHD-ATs* (Fig. 4g, h), and no sites showed positive selection in *SCPL-ATs* (Supplementary Fig. 45). This adaptive expansion may have resulted from purifying and diversifying selection and led to high levels of *BAHD-AT* and *SCPL-AT* transcripts in the berries (Fig. 4b), further explaining why piperine is uniquely accumulated in black pepper.

Overall, the main pathway leading to piperine synthesis and key genes involved in its regulation (Fig. 4i) are consequences of the expansion of multiple gene families, followed by evolutionary selection leading to transcription specifically in the berries. However, we fully recognise that a direct causal link between each identified gene and metabolite abundance remains to be established. Nevertheless, the genetic framework underpinning the evolution of piperidine alkaloid biosynthesis is being clarified.

## Discussion

The Piperales represents a useful taxonomic group for the study of the link between evolution, ecology and phytochemistry^41^ because of its geographic distribution pattern, the diversity of its lineages, and its characteristic secondary metabolism. The black pepper genome will provide resources for phylogenomic analyses and studies of piperine synthesis. Piperales is one of the most diverse lineages of basal angiosperms. In terms of stem anatomy, *Piper nigrum* has a pattern of vascular bundle arrangement that is similar to monocots, whereas the vascular bundle arrangement of *Piper colubrinum* is similar to eudicots^42^. In addition, the cotyledonary forms of *Peperomia pellucida*, *Peperomia peruviana* and *Peperomia parvifolia* show an evolution typical of eudicots to monocots. Interestingly, *Liriodendron* (Magnoliales) also shows features of both monocots and eudicots^6^. Because of their antiquity and morphological diversity, the phylogenetic positions of various lineages within the magnoliid clade have remained unclear, with three primary proposed tree topologies reported^6^. Two newly issued genome sequences have been used to address the phylogenetic position of magnoliids, but have produced conflicting results^6–8^. In our comparative genome analysis, a combination of three representative orders (Piperales, Magnoliales and Laurales) of magnoliids indicates that magnoliids are sisters to the monocots–eudicots clade, consistent with research into the *Liriodendron* genome^6^, the PPA tree^30^ and APG IV^5^. In addition, *Piper nigrum* is placed as a sister to Magnoliales–Laurales among the magnoliids (Fig. 2c). According to Soltis et al.^8^, the inclusion of complete angiosperm lineages that were missing in the present study (Nymphaeales, Austrobaileyales, Chloranthales, Ceratophyllales and Canellales in magnoliids) will facilitate a better understanding of the phylogenetic relationships of these diverse and long-isolated lineages of flowering plants.

The current assembly of the black pepper genome provides insights into the underpinning genetic changes following WGD events that are responsible for the unique biosynthesis of piperine. Our current study has focused on analysing the biological processes related to piperine biosynthesis and provides useful information on the significant expansion of gene families that are involved in piperine synthesis. Most notable is the discovery of the berry-specific expression of a series of relevant genes, including *LDC* genes involved in lysine metabolism; *GTF*, *CYP* and *HCT* genes in the phenylpropanoid pathway, and *BAHD-AT* and *SCPL-AT* genes (Fig. 4a). The phenylpropanoid, amino acid pathways and *ATs* are ubiquitous in plant secondary metabolism. *Coffea canephora*, *Malus domestica*, *Vitis vinifera*, *Theobroma cacao*, *Camellia sinensis*, *Ananas comosus* and *Citrus sinensis* are rich in derivatives of phenylpropanoids, and these species synthesise secondary metabolites through the convergence of the two metabolic pathways described above. One example is *Capsicum* (chili pepper) species, in which capsaicinoids are derived from the phenylpropanoid and branched-chain fatty acid pathways^43^. In addition, the precursors of nicotine alkaloids in tobacco (*Nicotiana*) species are derived from terpenoid and amino metabolism^44^. Lysine-derived quinoline alkaloids are synthesised through the convergence of phenylpropanoid and lysine metabolism in *Nelumbo nucifera*, *Papaver somniferum*, *Macleaya cordata*^27^ and *Carica papaya*^45^. However, piperine synthesis originates from the decarboxylation and amine oxidation of lysine, which distinguishes it from the polymerisation of two tyrosines in benzylisoquinoline alkaloid biosynthesis (Fig. 4i). Finally, two precursors derived from phenylpropanoid and lysine metabolism are catalysed by acyltransferase to produce piperine, and the associated genes exhibit gene expansion and high, berry-specific transcriptional activity in black pepper. The convergence of phenylpropanoid and lysine metabolism, specifically the decarboxylation and amine oxidation of lysine, and acyl transformation represent the characteristic features of piperine synthesis (Fig. 4i), and we describe the genetic and evolutionary basis of these features in this study.

Sequencing the black pepper genome has advanced our understanding of the unique piperine biochemistry of black pepper. Our study therefore provides valuable insights that may serve as a foundation for future research on Piperales taxonomy and piperine biosynthesis, leading to a better understanding of the evolution, phytochemistry and ecology of the *Piper* genus.

## Methods

### Leaf sample collection and DNA library construction

Fresh leaf tissues from single-living black pepper plants were collected to extract genomic DNA and RNA (Supplementary Note 1). For genome survey analysis, a short paired-end Illumina DNA library with a 350 bp insert size (137 × coverage) was sequenced on the Illumina HiSeq 2500 sequencer. For PacBio Sequel sequencing, 50 µg of high-molecular-weight (HMW) genomic DNA were prepared to generate five standard SMRTbell libraries with 20 Kb insertions. PacBio long reads were sequenced using 15 SMRTcells on the PacBio Sequel System (Pacific Biosciences) with SMRTbell Template Prep Kit 1.0-SPv3 (Pacific Biosciences). HMW genomic DNA was also prepared for 10 × Genomics libraries according to the manufacturer’s protocol (Chromium Genome v1, PN-120229). Sequencing-read libraries were sequenced using HiSeq 2500 with 2 × 150 paired-end reads to generate ~96 Gb (120 × coverage) raw data.

### Transcriptome library preparation and sequencing

RNA-seq experiments (three biological replicates) used RNA extracted from different organs of the black pepper Cv. Reyin1 (root, stem, leaf, flower and berries at four different stages: 2 months after pollination (MAP), 4 MAP, 6 MAP and 8 MAP) (Supplementary Fig. 35). Prepared libraries were sequenced on the Illumina HiSeq 2500 platform according to the manufacturer’s recommended protocol. We generated an average of 28.0 million paired-end reads for each sample.

RNA samples from black pepper Cv. Reyin1 leaves were also prepared for full-length transcriptome sequencing using the PacBio Iso-Seq protocol. The synthetic full-length cDNAs were selected to prepare a 20 Kb SMRTbell Template library for sequencing on a PacBio Sequel instrument.

### Preprocessing of PacBio Iso-Seq reads

The PacBio Iso-Seq3 pipeline (https://github.com/PacificBiosciences/IsoSeq3) was applied to obtain high-confidence transcriptome reads through the CCS (circular consensus sequence), classify, cluster and polishing process. The high-quality, full-length and consistent isoform transcript sequences were prepared for subsequent analysis.

### De novo genome assembly

The errors in the PacBio single-molecule real-time (SMRT) sequences were initially corrected using Canu^46^ with the default parameters. Because of heterozygosity and repeated sequences, FALCON^12^ was subsequently employed for de novo assembly using the corrected reads to produce primary contigs (p-Contigs). Then, FALCON-Unzip used the p-Contigs to perform phasing and directional classification of the heterozygosity from the initial assembly into updated primary Contigs (p-Contigs) and haplotigs (h-Contigs). Finally, the postprocessing step was used to polish using Arrow^47^ based on corrected PacBio long reads. The 10X Genomics Linked-reads were mapped to the consensus assembly described above using BWA-MEM^48^ with a default parameter. Then, fragScaff^13^ was used to extend contigs into initiatory scaffolds (Piper_nigrum_v1) according to the recommended scripts and processes of the fragScaff software.

### BioNano optical maps and hybrid assembly

Total of 750 ng of fresh young leaf tissues were collected from living plant material following BioNano Genomics guidelines. HMW genomic DNA with a fragment distribution ranging from 150 Kb to 2 Mb was fluorescently labelled using single-sequence-specific DLE-1 endonucleases (BioNano Genomics) based on the BioNano Direct Label and Stain (DLS) technology. The fluorescently labelled DNA was stained for at least two hours at room temperature and then loaded onto a Saphyr chips to scan on the BioNano Genomics Saphyr System by the sequencing provider Berry Genomics Corporation.

BioNano data were first filtered based on molecule length, mapping rate and label density using BioNano Solve (https://bionanogenomics.com/wp-content/uploads/2017/10/30182-Bionano-Tools-Installation-Guide.pdf). Non-haplotype de novo assembly was performed in BioNano Solve using filtered high-quality BNX files based on the Overlap–Layout–Consensus paradigm. The 350,823 filtered DLE-1 molecules (N50 length 0.288 Mb) produced 547 genome maps with an N50 of 3.8 Mb for a total map length of 1304 Mb. The de novo assembly containing BioNano molecules was combined with Piper_nigrum_v1 input into the BioNano Solve hybrid scaffolding pipeline to produce a hybrid scaffold assembly with -T *p*-value set to 1e-10 in both initial alignment, final alignment and hybrid scaffold steps, and more stringent value of 1e−11 for both the ‘merge_Tvalue’ and ‘T_cutoff’ *p*-value thresholds. Finally, the hybrid assembly had an N50 of 7.8 Mb, and the longest scaffold was 25.8 Mb. This stage of assembly was term Piper_nigrum_v2 (Supplementary Note 1).

### Scaffolding with Lachesis

About 5 g of fresh young leaf tissue from living plants were macerated and crosslinked using paraformaldehyde to capture the interacting DNA segments. Chromatin was subsequently digested with HindIII (NEB), and biotinylated nucleotides were used to fill in the resulting sticky ends. Following ligation, a protease was used to remove the crosslinks. Finally, genomic DNA was extracted, sheared into 350 bp fragments using a focused ultrasonicator (Covaris, Woburn, USA), and fragments into which biotin had been incorporated were pulled down with streptavidin-coated magnetic beads based. Purified DNA was then prepared and sequenced on an Illumina HiSeq instrument according to the manufacturer’s recommendations.

Hi-C paired-end reads were trimmed to remove low-quality bases and Illumina adapter sequences using Trimmomatic (http://www.usadellab.org/cms/?page = trimmomatic), and then checked with HiCUP^49^. The scaffolding began by aligning the clean Hi-C read pairs to Piper_nigrum_v2 using the aln and sampe commands from BWA^48^ with default parameters. Then, Lachesis (https://github.com/shendurelab/LACHESIS) with the cluster number set to 26 and other parameters set to the default values were used to cluster, order and orient the scaffolds. The oriented scaffolds were used to build the interaction matrices with juicer (https://github.com/aidenlab/juicer), inspect and manually correct with Juicebox assembly tools (https://github.com/aidenlab/Juicebox).

### Post-processing

We also performed an additional round of gap filling to eliminate the gaps generated in the final scaffolding steps using PBJelly (https://github.com/alvaralmstedt/Tutorials/wiki/Gap-closing-with-PBJelly) with PacBio subreads. The assembled scaffolds were further polished with Pilon (https://github.com/broadinstitute/pilon) using Illumina paired-end reads to correct base errors. The ultimate assembly consisted of 45 scaffolds with a total size of 761.2 Mb and a scaffold N50 of 29.8 Mb. This assembly is designated as final version of black pepper genome: Piper_nigrum_v3.

For an assessment of completeness, the Piper_nigrum_v3 genome was subjected to a BUSCO analysis^16^ and compared with the Viridiplantae_odb10 database (Update date: 2017–12–01) with the --long parameter. In addition, Core Eukaryotic Genes (CEGs) were also aligned to Piper_nigrum_v3 using CEGMA^15^ with the default parameters.

### Genomic annotation

We used a combination of the de novo repeat library and homology-based strategies to identify repeat structures. TransposonPSI (http://transposonpsi.sourceforge.net/) was used to identify transposable elements; GenomeTools suite^50^ (LTRharvest and LTRdigest) was used to annotate LTR-RTs with protein HMMs from the Pfam (Supplementary Table 6) database (Supplementary Note 2). Then, a de novo repeat library of black pepper was built using RepeatModeler software (http://www.repeatmasker.org/RepeatModeler/), and each of the three repeat libraries was classified with RepeatClassifier, followed by Merge and de-redundancy using USEARCH (https://www.drive5.com/usearch/) with ≥ 80% identity. Subsequently, the non-redundant repeat library was analysed using BLASTX to search the transposase database (-evalue 1e-10) and non-redundant plant protein databases (-evalue 1e-10) to remove protein-coding genes. Unknown repetitive sequences were further classified used CENSOR (https://www.girinst.org/censor/index.php). Then, the de novo repeat library was used to discover and mask the assembled genome with RepeatMasker (http://www.repeatmasker.org/RMDownload.html) with the -xsmall parameter.

For gene structure annotations, the Illumina RNA-seq data from black pepper Cv. Reyin1 were aligned to repeat-softmasked genome using GSNAP^51^, which generates intron hints with other aligned hints (ESTs, proteins and nucleotides from NCBI) for gene structure annotation. For PacBio Iso-Seq reads, GMAP^52^, a splice-aware aligner, was used to align the high-quality isoform transcripts to the repeat-softmasked genome for the detection of new isoforms.

The structural annotation of protein-coding genes was performed using BRAKER2^18^, which integrates GeneMark-ET and AUGUSTUS by combining the aligned resulted from ab initio predictions, homologous protein mapping, RNA-seq mapping and GMAP PacBio mapping to produce the final gene set. Gene models from these different approaches were combined using the EVM software (version 1.1.1)^53^. Moreover, tRNA loci, rRNA, lncRNAs, snRNA and miRNAs and non-protein-coding genes were annotated by performing homologous searching and deep-learning analyses across the assembled genome sequence (Supplementary Note 3).

### Functional annotation of protein-coding genes

Predicted genes were subjected to functional annotation by performing a BLASTP homologue search against the UniProtKB Viridiplantae database, and the NCBI non-redundant protein database with an e-value threshold of 1e-10. In addition, a comprehensive annotation was also achieved using InterProScan (v5.31–70.0)^54^, which includes motifs/domains prediction, functional classifications, protein family identification, transmembrane topology, predicted signal peptides and GO annotations. KAAS (https://www.genome.jp/kegg/kaas/) and KOBAS 3.0 (http://kobas.cbi.pku.edu.cn/) were used to search the KEGG GENES database for KO (KEGG Orthology) assignments and generating a KEGG pathway membership. PlantTFcat^55^ was also used to systematically analyse InterProScan domain and categorise possible chromatin regulators (CRs), transcription factors (TFs) and other transcriptional regulators (TRs) in the current assembly. The plantiSMASH version 3.0.5-a04b4cd^56^ software was used to cluster the plant-specific secondary metabolism genes.

### Transcriptome assembly and gene expression analysis

RNA-seq reads from three replicates of the eight tissue types were preprocessed using Trimmomatic by removing adaptor sequences and filtering low-quality reads. HISAT2 was used to align the samples with the genome for genome-guided transcript assembly using StringTie^57^.

Read counts extracted from StringTie were filtered using the sva R package to decrease batch effects and hidden variables. Differentially expressed genes (DEGs) were detected using DESeq2^58^, and calculated based on absolute log2 transformed fold-change values greater than 2 and *p*-value of 0.05 using the Benjamini–Hochberg correction^59^. A gene set enrichment analysis (GSEA)^33^ was performed to determine significant gene sets, and the WGCNA package^34^ in R was applied to perform a multivariate analysis of gene co-expression modules. See Supplementary Note 6 for additional details.

### Synteny analyses

For estimation of the time of whole-genome duplication events, BLASTP reciprocal best hit (RBH) pairwise sequences of paralogous (within the species genome) and orthologous relationships (between black pepper and other species) were identified and the synonymous divergence levels (*K*_s_) were calculated using the YN model in KaKs_Calculator v2.0^60^. The raw *K*_s_ distributions were used to fit a mixture model of Gaussian distributions and thus derive the mean *K*_s_ values between paralogs and orthologues in R package Mclust 5. The *K*_s_ distributions of orthologues between black pepper and other species were adopted to compare the relative substitution rates in different species by plotting with the ggplot2 package. The divergence time of black pepper was calculated by combining the *K*_s_ value with synonymous substitutions at each site per year (*r*) for magnoliids through the formula: divergence date (*T*) = *K*_s_/*2r*. Synteny and syntenic block gene pair analyses were performed using MCScanX and the CoGe Comparative Genomics Platform (Supplementary Note 4).

### Comparative genomics for phylogenomic and gene family

Putative orthologous genes were constructed from nine eudicots, three monocots, three magnoliids, *Amborella*, two gymnosperms and the outgroups *Selaginella moellendorfii* and *Physcomitrella patens* were inferred using OrthoMCL^61^ and compared with protein genes from the current assembly genome of black pepper to investigate the evolution and phylogenetic placement of black pepper among seed plants (Supplementary Note 7). The total of one-to-one orthologous gene sets were identified and extracted for alignment using muscle^62^ and further trimmed using Gblocks 0.91b^63^. A maximum likelihood phylogenetic tree was constructed using concatenated alignment with RAxML v8.2.12^64^ and the PROTGAMMAILGF model to automatically determine the best reasonable tree by conducting 1000 bootstrap replicates. The maximum likelihood tree was used as a starting tree to estimate species divergence time using BEAST v2.1.2 (Bayesian Evolutionary Analysis Sampling Trees2)^65^. A Calibrated Yule model with a Strict Clock rate and gamma hyperparameter of prior distribution were used to estimate the divergence time. Speciation event dates for *Ananas comosus*-*Oryza sativa* (Normal model, Mean: 105 MYA, Sigma: 0.5) and *Liriodendron chinense*-*Cinnamomum kanehirae* (Normal model, Mean: 118 MYA, Sigma: 0.5), which were obtained using Timetree web service (http://www.timetree.org/), *Papaver somniferum*-*Nelumbo nucifera* (Normal model, Mean: 125 MYA, Sigma: 0.5)^22^, monocots–eudicots (Log Normal model, Mean: 150 MYA, Std dev: 4MYA), were used to calibrate the divergence time. The Markov chain Monte–Carlo analysis was repeated 10,000,000 times with 1000 steps.

The orthologous genes and phylogenetic tree topology inferred from the OrthoMCL analysis were taken into CAFE v4.2^66^, which employed a random birth and death model, to estimate the size of each family at each ancestral node and obtain a family-wise *p*-value (based on a Monte–Carlo re-sampling procedure) to indicate whether has a significant expansion or contraction occurred in each gene family across species

### Identification of alkaloid and piperine biosynthesis genes

Predicted protein-coding genes from the black pepper genome were aligned against annotated alkaloid and piperine biosynthesis pathway genes in the KEGG database by BLASTP with an e-value cut-off of 1e-5, identity > 50% and alignment coverage ≥ 50%. In addition, all orthologous gene sequences involved in alkaloid and piperine biosynthesis were also captured from comparative genomics analyses, and corresponding gene functions were verified by searching the published literature and the NCBI and UniProt databases.

### Sequence evolution of gene families

Gene families derived from OrthoMCL identification were aligned using ClustalW (http://www.clustal.org/clustal2) and trimmed with trimAl (v1.2) (http://trimal.cgenomics.org/introduction). Evidence of selection across genes within gene families was tested using a multispecies alignment in HyPhy with the datamonkey webserver^67^. The genetic algorithm recombination detection method^68^ was performed to detect breakpoints at nucleotide sites. Evidence of positive selection and episodic selection sites in genes was inferred by estimating the rates of synonymous and non-synonymous changes at each site in the aligned sequence through single likelihood ancestor counting (SLAC)^69^ and a mixed effects model of episodic diversifying selection (MEME)^70^ (Supplementary Note 8).

### Reporting summary

Further information on research design is available in the Nature Research Reporting Summary linked to this article.

## Supplementary information

Supplementary Information

Peer Review

Reporting Summary

Source Data

## Data Availability

The data supporting the findings of this work are available within the paper and its Supplementary Information files. A reporting summary for this Article is available as a Supplementary Information file. The data sets generated and analysed during this study are available from the corresponding author upon request. All the raw sequencing data generated during this study have been deposited at NCBI as a BioProject under accession PRJNA529758. Transcriptome sequence reads have been deposited in the SRA database under BioProject number PRJNA529760. The genome assemblies and annotation files are available at the website http://cotton.hzau.edu.cn/EN/download.php. The source data underlying Figs. 1, 2a, b, 3 and 4a–h are provided as a Source Data file.
