# Supplementary material for: The chromosome-scale reference genome of black pepper provides insight into piperine biosynthesis
Source: Nat Commun. 2019 Oct 16;10:4702. doi: 10.1038/s41467-019-12607-6 (PMC6795880; doi:10.1038/s41467-019-12607-6)
Supplement: Supplementary file 1 — Supplementary Information [file 41467_2019_12607_MOESM1_ESM.pdf]

The chromosome-scale reference genome of black pepper provides insight into piperine biosynthesis

Hu *et al.*

Supplementary Note 1

Sample collection and DNA extraction

'Reyin1', one of the black pepper cultispecies derived from elite cultivar 'Lampung Daun Kecil' of Asia, which accumulates piperine in berry and is grown in the Flavor Beverage Institute, Chinese Academy of Tropical Agriculture Science, Hainan, was chosen for the sequencing and assembly of the black pepper reference genome. The fresh leaf tissues were collected from a single living plant into the gaseous phase of liquid nitrogen (-196 °C) and then stored at -80 °C. For Hi-C and BioNano optical map sequencing, the fresh leaf was used for experimental treatments.

Assessment of the genomic size

The genome size and heterozygosity were evaluated by k-mer (k=17) distribution analysis with Jellyfish[1] and GCE[2] using 350 bp Illumina paired-end reads (102.8 Gb). Notably, our k-mer (k=17) distribution displayed two main distinct peaks (left peak: heterozygous regions and right peak: homozygous regions) (Supplementary Figure 1). Based on two hypotheses, all the k-mer distributions obtained from sequenced reads traverse the entire genome and the frequencies of a k-mer along the sequence depth gradient follow a Poisson distribution, the genome size (G) is defined as G = k-mer number/k-mer depth, where the k-mer number is the total number of k-mers, and k-mer depth is the frequency occurring more frequently than other frequencies. The 17-kmer analysis captured a k-mer number of 78,519,660,276 and main peak depth of 101 in a plot of the frequency distribution of k-mer numbers, suggesting that the *P. nigrum*

genome is approximately 761.74 Mb. The secondary peak that has just half of the average sequencing depth of the primary peak reveals high heterozygosity (1.33%), and the percentage of k-mer numbers after the homozygous peak at 1.8 of the total number of k-mers shows a repetitive sequence ratio of 59.54%.

Genome assembly

Given the challenges of high heterozygosity (1.33%) and repetitive sequences (59.54%) (Supplementary Table 1 and Supplementary Figure 1), we adopted a comprehensive assembly strategy in this project (Supplementary Figure 2). The PacBio long reads span repeat-rich and heterozygous genomic regions, to effectively overcome the challenges in plant genome assembly. Chromium 10X data was also utilized to support scaffold validation and allow further elongation of the phased scaffolds (Piper_nigrum_v1).

We performed scaffolding of Piper_nigrum_v1 assembly using the BioNano optical maps sequence. DLS labelled DNA was loaded into a nanochannel array of a Saphyr Chip (BioNano Genomics) and imaged using the Saphyr system and associated software (BioNano Genomics). Notably, 3,433,888 BioNano molecules with a molecule N50 0.176 Mb for molecules above 20 Kb and 0.266 Mb for molecules above 150 Kb were obtained with an average label density of 14.21/100 Kb for molecules above 150 Kb. The map rate was 50.6% for molecules above 150 Kb. The effective coverage was 128X.

The BioNano data were filtered and *de novo* assembly was performed using BioNano Solve v3.2.1 software. The assembly type performed was the "non-haplotype" with "no extend split" and "no cut segdups" (optArguments_nonhaplotype_noES_noCut_DLE1_saphyr.xml). A more stringent strategy was used according to the manufacturer's guidelines to overcome the higher heterozygosity and polyploidy in the black pepper genome. A total of 350,823 filtered DLE-1 molecules with an N50 of 0.288 Mb (theoretical coverage of the reference 74x) produced 547 maps with an N50 length of 3.8 Mb and a total length of 1,304 Mb (coverage = 23x).

For the DLE-1 scaffolding, HybridScaffold config file hybridScaffold_DLE1_config.xml was used as default settings. The autoNoise1.errbin file from *de novo* assembly of BioNano molecule that without reference was also used as an auto-noise. Despite undergoing filtering and under a more stringent strategy, many conflict sites remained between the PacBio assembly sequence and BioNano optical maps *de novo* assembly because of high heterozygosity and repetitive sequences in the black pepper genome. We reduced the redundancy in the PacBio long read assembly using Falcon, but not in BioNano Solve arithmetic at present. Therefore, we selected to cut the BioNano contigs and retain PacBio assembly at the conflict sites (the software Hybrid-scaffold parameter of '-B 2 -N 1'). Finally, we visualized the genome map using BioNano Access (https://bionanogenomics.com/support-page/bionano-access-software/) and manually examined the conflict sites together with mapping Illumina paired-end reads and PacBio long reads to conflict regions. Then, the genomeCoverageBed[3,4] command with the "-d"

parameter was used to define the coverage of each base (including the bases that are covered by no reads), and coverage files were employed to verify whether the connections were authentic and reliable. If the cut was inappropriate, we edited the assignAlignType/cut_conflicts/conflict_cut_status.txt file, and reran the hybrid scaffold pipeline using the "-M" option along with the newly edited status file. The resulting DLS hybrid assembly had an N50 of 7.8 Mb for a total length of 837 Mb and consisted of 201 scaffolds (Piper_nigrum_v2). We then conducted additional scaffolding using Hi-C data, followed by gap filling using corrected PacBio long reads and consensus polishing using Illumina paired-end reads (Piper_nigrum_v3).

SNP calling for heterozygosity

BWA-MEM[5] was also used to remap the final assembled Piper_nigrum_v3 genome with Illumina paired-end reads to calculate the observed heterozygosity. SAMtools[6] sorted aligned results were marked and duplicates were removed using Picardtools, followed by SNP calling and filtering (QUAL > 20) using GATK[7]. The heterozygosity of each scaffold was calculated using GWASTools[8] with the hetByScanChrom function.

Supplementary Note 2

Annotation of repeat DNA sequences

For the LTR-RT annotation, Profile HMM files were selected from Pfam[9] (http://pfam.xfam.org/search#tabview=tab2) using the search terms "retrotransposon",

“env transposon”, “reverse transcriptase”, “retroelements” and “gag transposon”. The resulting list in matching Pfam families was subsequently checked via click to enter: Species → Tree. Only the results belonging to Viridiplantae were added to the final set (Supplementary Table 6). When using LTRdigest, this set is organized as a directory containing the downloaded pHMM files, which represent an argument for using the “-hmms” parameter.

For repeat annotation, we first removed unknown sequences from non-redundant sequences using RepeatClassifier, resulting in identified and unknown sequences. The unknown sequences were searched with BLASTX against a transposase database with “-evalue 1e-10”. Then, the hits were combined with identified sequences into ModelerID.lib and other sequences were classified into ModelerUnknown.lib. Gene fragments were excluded from these two files using ProtExcluder (http://www.hrt.msu.edu/uploads/535/78637/ProtExcluder1.2.tar.gz) by searching a plant protein database (customized python script), which contains sequences from SwissProt plant proteins (ftp://ftp.uniprot.org/pub/databases/uniprot/current_release/knowledgebase/taxonomic_divisions/uniprot_sprot_plants.dat.gz, 2018) and NCBI Refseq plants (using Entrez Direct: https://www.ncbi.nlm.nih.gov/books/NBK179288/). Protein sequences were also searched against the NCBI EST database (TBLASTN, e=10-5), and only sequences with a match were retained. Second, the remaining sequences were searched against the transposase database (BLASTP e=10-5) mentioned above, and sequences with matches were excluded. Finally, the sequences were combined with KnownRepeats

(ModelerID.libnoProtFinal) and the ModelerUnknown (ModelerUnknown.libnoProtFinal) library into a *de novo* repeat library, which was ranRepeatMasker on the assembled genome with -xsmall parameter.

Comparison of transposable elements

The repeat family identification approach for black pepper was used to exquisitely annotate transposable elements (TEs) of species employed in the phylogenomics analysis (see below). The percentage of TEs in black pepper (~ 54.01%) was higher than in the other magnoliids (*Liriodendron chinense* (~ 44.59%) and *Cinnamomum kanehirae* (~ 33.41%)), *Amborella trichopoda, Selaginella moellendorffii* and nearly equivalent to that in *P. patens*. Among the retrotransposons (Class I), LTR retrotransposons were more prevalent than NonLTR retrotransposons and were the most dominant type of repeats in all species. A comparison with *Arabidopsis thaliana* indicated higher proportions of LTR retrotransposons in magnoliids (Supplementary Table 8). In addition, LTR/Gypsy members displayed a greater percentage than that of members of the LTR/Copia superfamilies, except in *Dendrobium officinale* (LTR/Gypsy: 19.95% and LTR/Copia: 45.22%) and *Nelumbo nucifera* (LTR/Gypsy: 35.89% and LTR/Copia: 47.81%). NonLTR retrotransposons are less prevalent in magnoliids than they are in *Amborella trichopoda*. Compared to monocots and eudicots, the LTR/Gypsy families of repeats appear to have expanded in magnoliids and lower plants. Conversely, LTR/Copia repeats appear to have contracted in lower plants (Supplementary Table 9). Large differences in the Gypsy-to-Copia ratio were observed among the species, with the largest differences of ~15.5 and 13.9 observed in lower plants, followed by smaller differences in

magnoliids (~3.7 to ~1.5) and angiosperms (~9.6 to ~0.4) (Supplementary Table 9). The proportion of DNA transposons (Class II) in *Cinnamomum kanehirae* (17.9%) was comparable to that in black pepper (21.5%) but higher than that in *Liriodendron chinense* (5.6%). The miniature inverted-repeat transposable element (MITE) accounted for 4.0% of transposons in black pepper — a larger fraction of the genome than in similarly sized plant genomes, including the genomes of *Cinnamomum kanehirae* and *Nelumbo nucifera*. However, the Helitrons were less frequent in black pepper (~0.44%), *Liriodendron chinense* (~0.45%) and *Amborella trichopoda* (~0.16%), than they were in *Cinnamomum kanehirae* (~1.07%) (Supplementary Figure 16).

Supplementary Note 3

Non-coding RNA annotation

Next, tRNA loci (tRNAScan-SE[10]), rRNA (RNAmmer[11]), lncRNAs (intersection of PLE[12], PLncPRO[13], RNAplonc (http://rnaplonc.cp.utfpr.edu.br/about.php)), snRNA and miRNAs (RfamScan[14]) and non-protein coding genes were annotated by performing homologous searching and deep learning across the assembled genome sequence.

For the RfamScan analysis, Infernal[15] was used to search the black pepper genomic sequences in the Rfam library of CMs from ftp://ftp.ebi.ac.uk/pub/databases/Rfam/CURRENT/Rfam.cm.gz and Rfam clanin file from ftp://ftp.ebi.ac.uk/pub/databases/Rfam/CURRENT/Rfam.clanin for RNA structure annotations. In total, 256 miRNA genes were predicted and classified into 26 miRNA families (Supplementary Figure 27) and 1533 snRNAs.

Supplementary Note 4

Genome synteny and polyploidization

We first performed a self-alignment of the assembled genome sequence using SynMap in the CoGe Comparative Genomics Platform[16] and merged the syntenic blocks using Quota Align Merge algorithm with the default parameters to reveal the evolution of the black pepper genome. The analysis revealed long stretches of duplications within the black pepper genome that are either inter-chromosomal (between chromosomes 1 and 13, 2 and 8, 3 and 15, 4 and 12, 5 and 7, 6 and 24, 9 and 25, 10 and 11) or intra-chromosomal (Pn4 and Pn8) duplications (Supplementary Figure 30). Then, we performed an all-vs-all paralog analysis in the black pepper genome using the reciprocal best hit (RBH) and calculated the synonymous substitution rate (K_s) of RBH gene pairs using KaKs_Calculator v2. 0[17] based on the YN model. We detected a single K_s peak at approximately 0.1 through the *Ks* distribution of 31,138 RBH paralogous gene pairs with K_s greater than 0.02 and less than 3. We also performed a synteny analysis of the black pepper genome using MCScanX[18] with the default parameters and calculated the K_s distribution of syntenic block gene pairs to distinguish whether this peak represents a whole genome duplication event or background small-scale duplication, as observed in the opium poppy genome[19]. The results clearly show a major peak at around 0.1 (Supplementary Figure 32). In addition, the syntenic K_s distribution reveals a minor peak at approximately 0.8, indicating that the black pepper genome has undergone additional segmental duplications.

Supplementary Note 5

Piperine determination

High-performance liquid chromatography (HPLC) was used to determine piperine content in pepper berry and tissues, as described[20]. Briefly, all fruit samples were powdered after vacuum freeze drying. Ethanol (95% [m m^-1]) was used for piperine extraction as previously described[20]. The mobile phase (methanol/H2O, 77:23 [v v^-1]) was used to perform HPLC at a flow rate of 1 ml min^-1. The identification and quantitation of piperine were performed by comparing the characteristic retention time and relative peak area of the piperine standard purchased from Sigma-Aldrich (purity: 97.0%).

Supplementary Note 6

Transcriptome data

RNA-seq libraries were statistically analysed using FastQC[21] and results were aggregated with MultiQC[22], as described in Supplementary Figure 36. We also performed a quality assessment of each tissue through sample clustering and visualization (Supplementary Figure 37).

Statistical analysis and visualization of transcriptome data

Unless stated otherwise, all statistical analyses were performed with R version 3.4.2 (R Core Team 2017). Heatmaps were generated with the R pheatmap[23] function. Circular plots of tissues (average of three biological repeats) were generated with circos v0.69-4 and circos helper tools v0.67[24] (Fig. 1). The distribution of differentially expressed genes was displayed using karyoploteR package[25] (Supplementary Figure 38).

Transcriptomic study linked to piperine biosynthesis

Differentially expressed genes in berry were analysed using the DESeq2[26]. Count matrices were used as the input, as specified in the package manual. The IHW[27] package was used to adjust the p-value, with an FDR cut off of 0.05. Differentially expressed genes were further divided into up- or down-regulated genes, depending on the sign of the fold change (FC).

Cytoscape visualization of the WGCNA network

Cytoscape[28] was used to visualize the module network obtained from the WGCNA analysis, and modules that contained genes required for piperine biosynthesis were selected. The MCODE[29] clustering algorithm was used to cluster all densely connected regions to a highly interconnected region. “Attribute Circle Layout” with “MCODE_Node_Status” automatic layout algorithms was performed to arrange all nodes. The genes in different clusters were marked with different colors and shapes. Lines in different colors indicate the connections with specific genes (Supplementary Figure 43).

Supplementary Note 7

Phylogenomic analysis

Putative orthologous genes were constructed from nine eudicots (*Coffea canephora*[30], *Capsicum annuum*[31], *Camellia sinensis*[32], *Vitis vinifera*[33], *Citrus sinensis*[34], *Nelumbo nucifera*[35], *Papaver somniferum*[19], *Macleaya cordata*[36] and *Arabidopsis thaliana*[37]), three monocots (*Oryza sativa japonica*[38], *Ananas comosus*[39] and *Dendrobium officinale*[40]), three magnoliids (*Liriodendron chinense*[41], *Cinnamomum kanehirae*[42] and *Persea americana* (transcriptome datasets)[43]), one Amborella species (*Amborella trichopoda*[44]), two gymnosperms (*Gnetum montanum*[45] and *Picea abies*[46]) and the outgroups *Selaginella moellendorfii*[47] and *Physcomitrella patens*[48] were inferred using OrthoMCL[49] and compared with protein-coding genes from the current assembly genome of black pepper to assess the evolution and phylogenetic placement of black pepper among seed plants.

Supplementary Note 8

Evolution of gene families related to piperine biosynthesis

The orthologous gene clusters from the black pepper genome and twenty other sequenced plant species (used in phylogenomic analysis of black pepper) were identified using OrthoMCL[49] to investigate the evolutionary processes of piperine biosynthesis. Redundant and incomplete protein sequences in all genomes were discarded. CAFE v4.2[50] and custom scripts were employed to identify family expansion and contraction.

We then tested for evidence of selection across gene families related to piperine biosynthesis in HyPhy[51] using the datamonkey webserver[52]. The aligned and trimmed gene family files were first used to screen for evidence of recombination and topological incongruence with a breakpoint at nucleotide positions via the Genetic Algorithm for Recombination Detection (GARD) method[53]. Then, we proceeded to a subsequent selection analysis (SLAC[54] and MEME[55]) with a significance threshold of $\alpha = 0.1$.

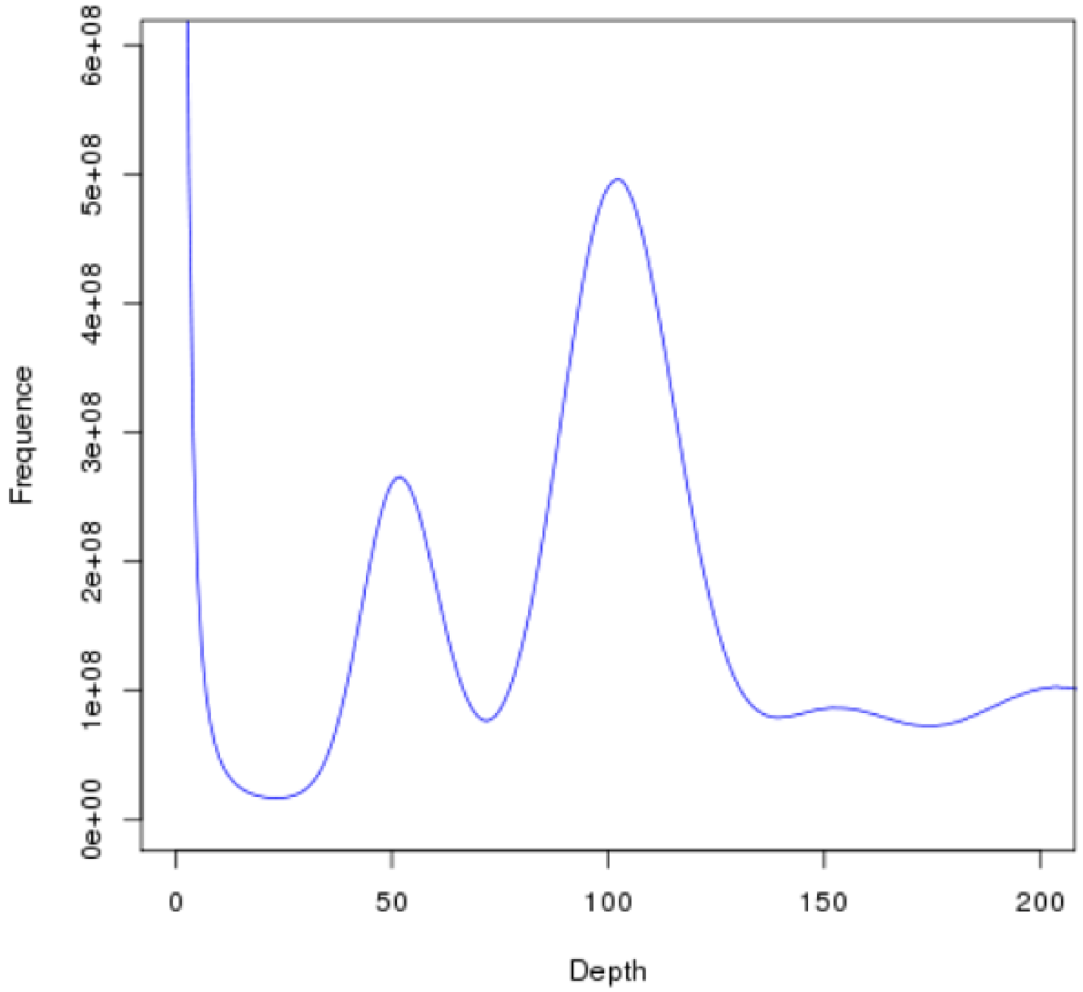

Supplementary Figure 1. Kmer frequency distributions. When k-mer=17, a frequency peak value at 101 is observed and used to estimate the genome size.

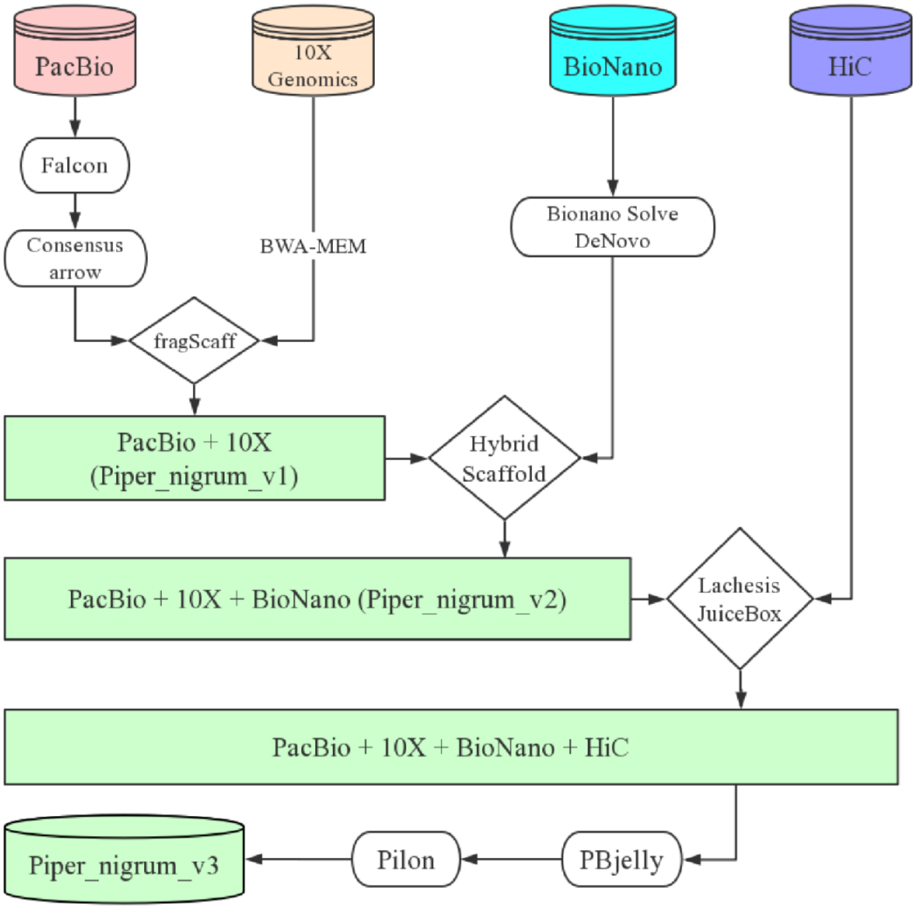

Supplementary Figure 2. Overview of the processing pipeline used to assemble the black pepper genome. Four datasets, PacBio reads, 10X Genomics Linked-reads, BioNano molecule and Hi-C mapping, were used for hybrid assembly strategy. PacBio reads were used to performed contigs assembly and preliminary extension with Linked-reads from 10X Genomics sequencing, which were defined as "Piper_nigrum_v1" assembly version. Subsequently, the BioNano DLS optical mapping was used to order and orient these scaffolds into superscaffolds (Piper_nigrum_v2), and Hi-C mapping was used to anchor and orient the scaffolds into pseudomolecule. The additional round of gap filling and polished were performed to yield final version of assembly "Piper_nigrum_v3".

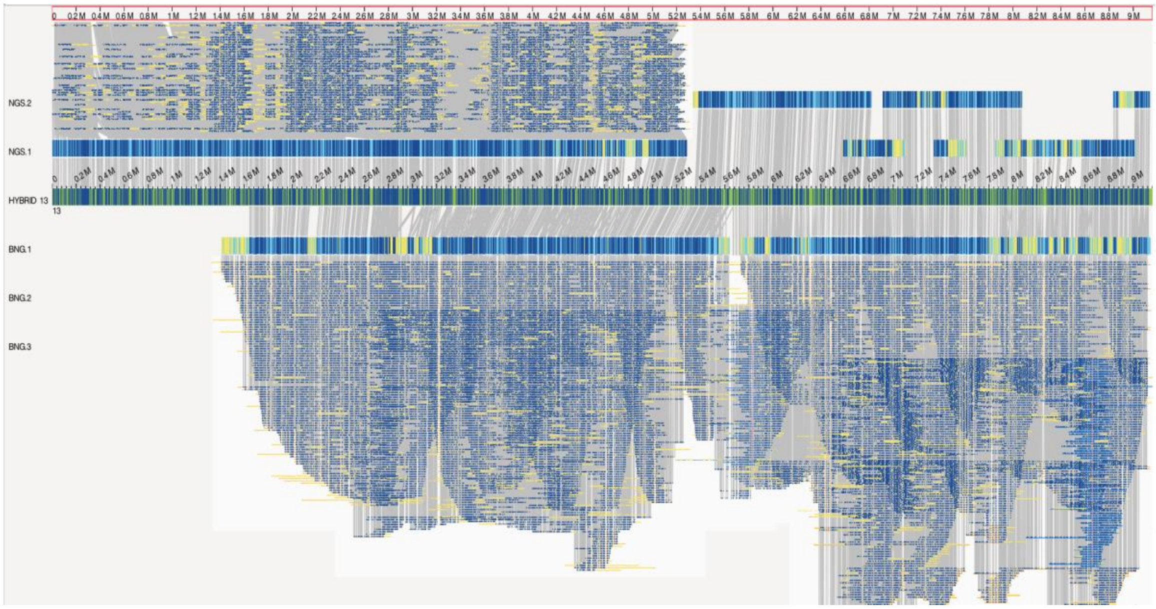

Supplementary Figure 3. Use of BioNano molecules to extend and connect scaffolds from the PacBio and Chromium 10X assembly. The dark blue bar in the middle represents the assembled scaffold based on NGS and BioNano molecules. The blue bar above represents the assembled scaffolds based on NGS, and below represents the assembled BioNano molecules. Each blue line represents a BioNano molecule and yellow represents the molecule labels.

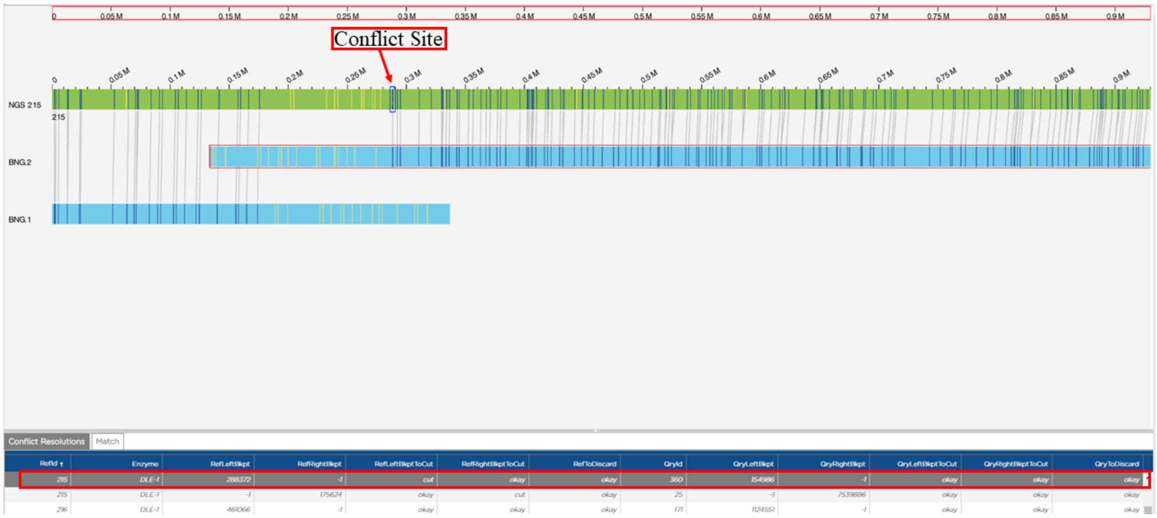

Supplementary Figure 4. A conflict site that occurred during the BioNano hybridScaffold step. The green bar represents an assembled scaffold based on NGS reads and cyan represents BioNano molecules. Yellow lines in the bars represent the molecule labels. The grey line will connect the scaffold and BioNano molecules when this region has corresponding labels. Otherwise, it is defined as a conflict site.

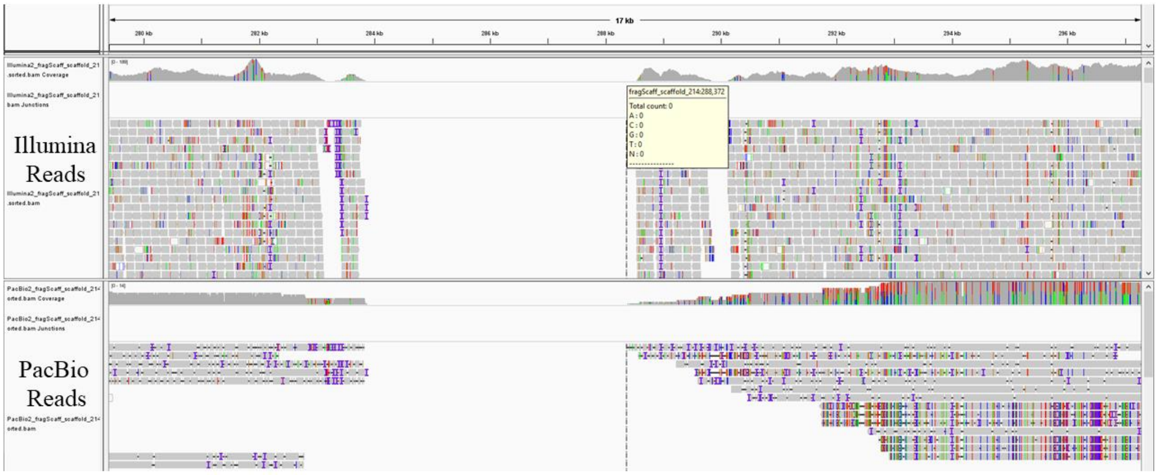

Supplementary Figure 5. Map of the Illumina and PacBio long reads to Piper_nigrum_v1 to examine the conflict sites. The mapped BAM files were visualized in IGV.

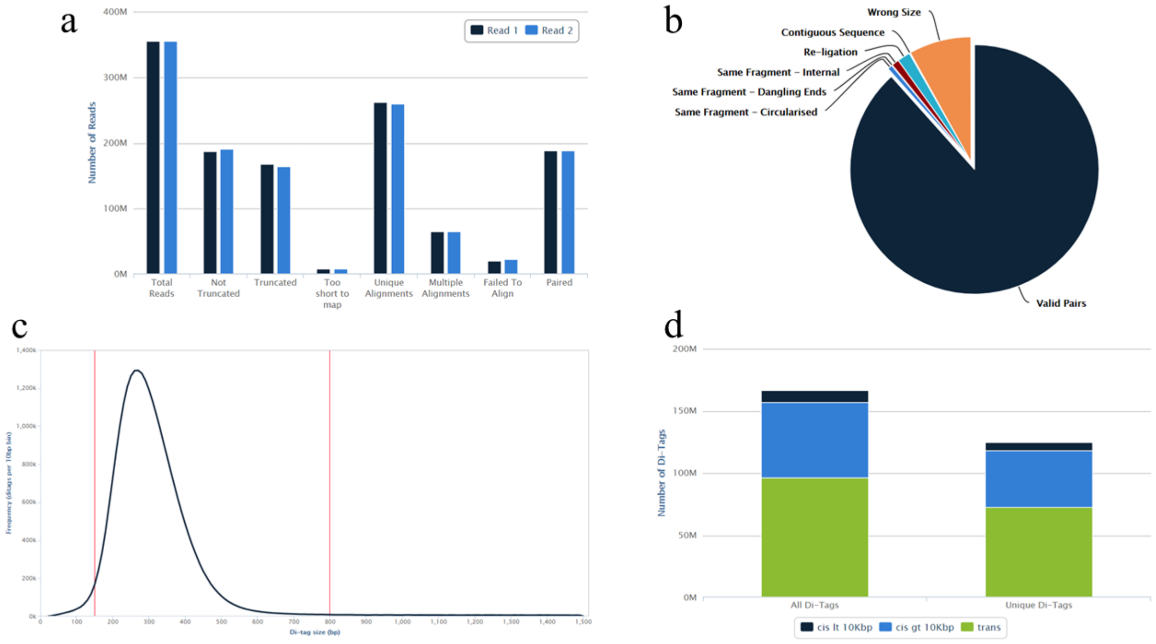

Supplementary Figure 6. HiCUP report of Hi-C data. (a) Statistics of truncated and mapped reads. (b) Statistics of reads after filtering. (c) Di-tag length distribution. (d) De-duplicated reads.

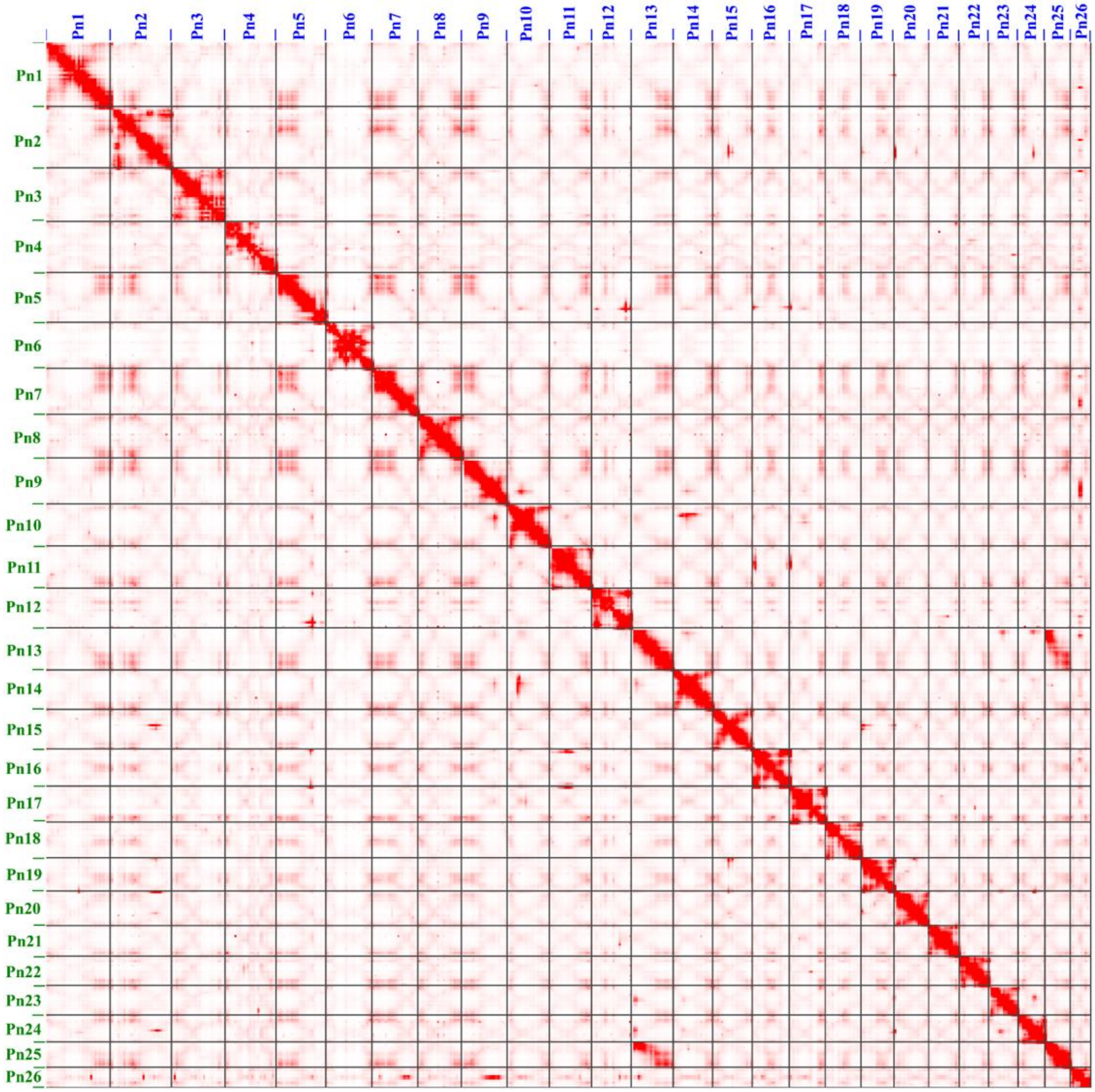

Supplementary Figure 7. Hi-C map of the black pepper genome showing genome-wide all-by-all interactions. The map shows high-level interactions that occur within chromosomes (cis) rather than between chromosomes (trans).

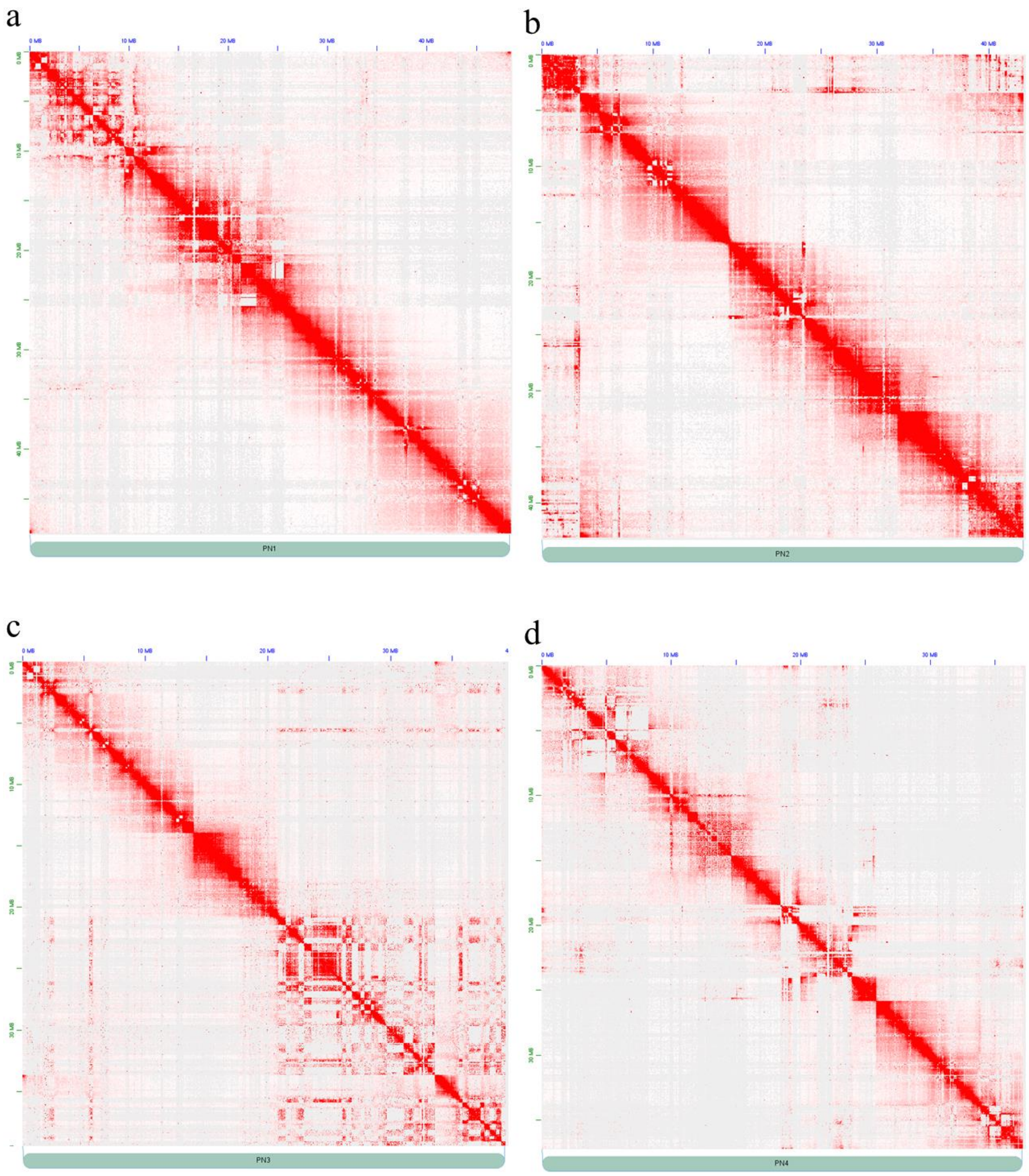

Supplementary Figure 8. Chromatin interactions in chromosome 1 to 4 of black pepper. (a-d) Each heatmap shows the observed values at a resolution of 100 Kb and normalization with balancing.

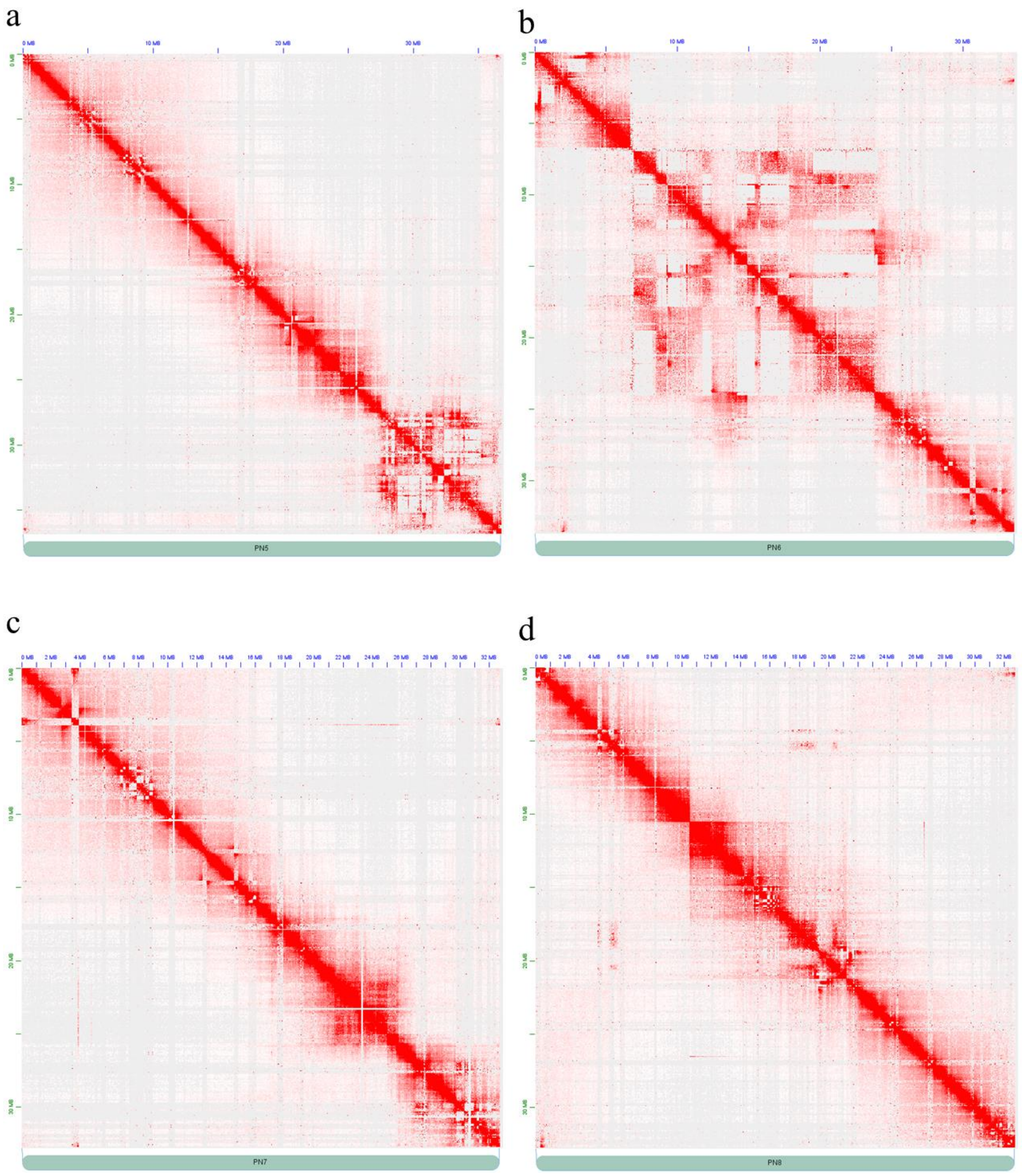

Supplementary Figure 9. Chromatin interactions in chromosome 5 to 8 of black pepper. (a-d) Each heatmap shows the observed values at a resolution of 100 Kb and normalization with balancing.

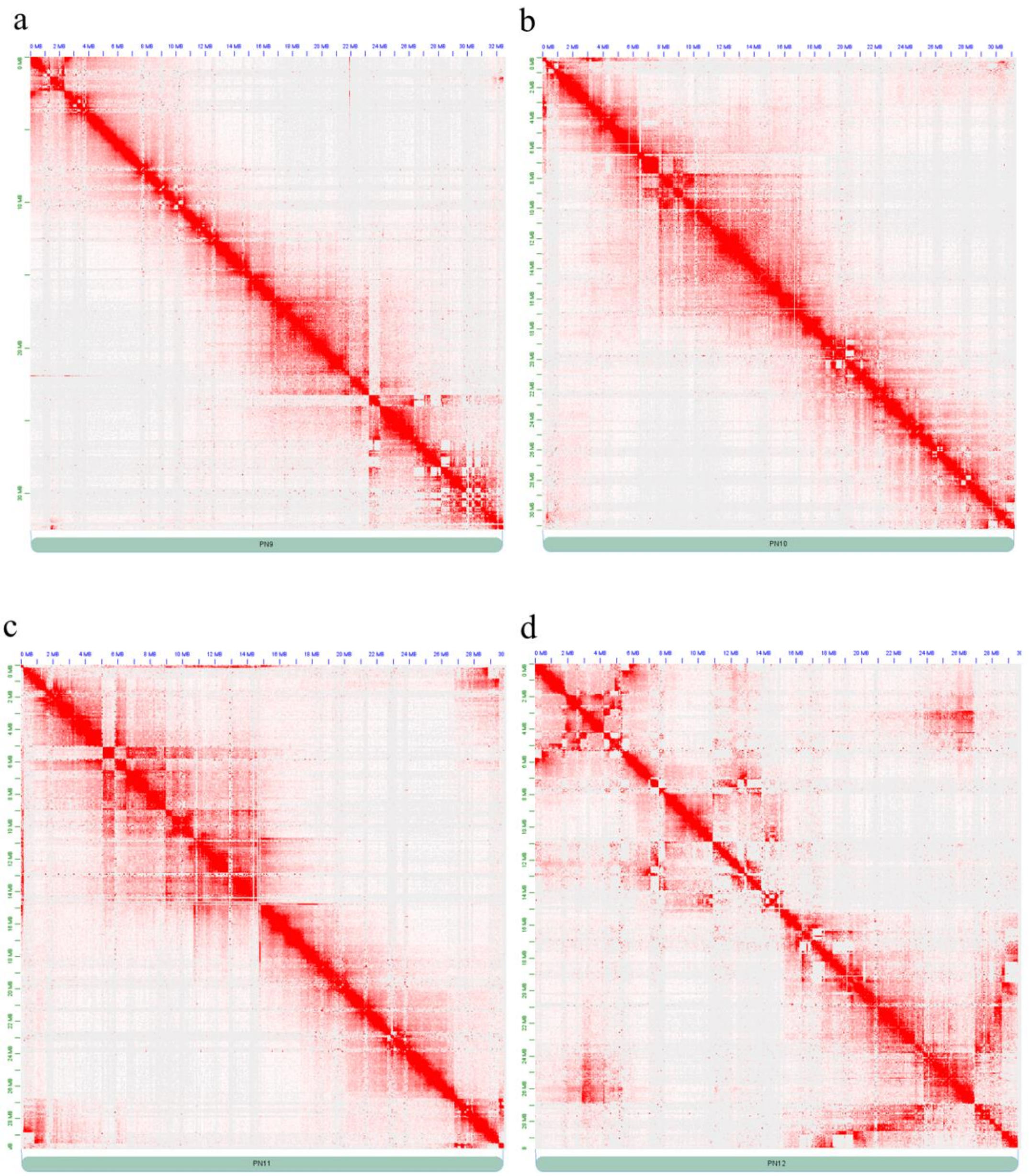

Supplementary Figure 10. Chromatin interactions in chromosome 9 to 12 of black pepper. (a-d) Each heatmap shows the observed values at a resolution of 100 Kb and normalization with balancing.

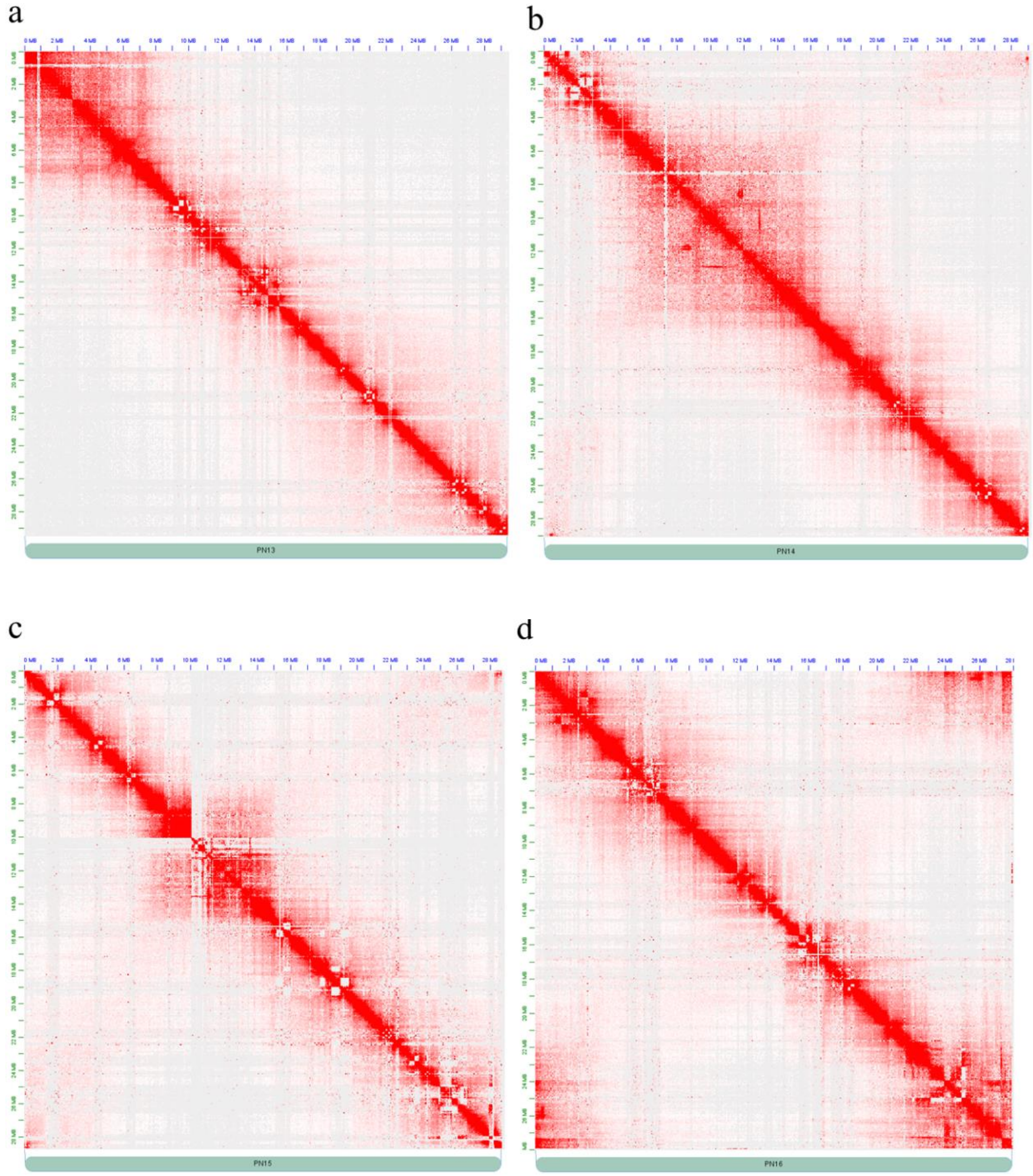

Supplementary Figure 11. Chromatin interactions in chromosome 13 to 16 of black pepper. (a-d) Each heatmap shows the observed values at a resolution of 100 Kb and normalization with balancing.

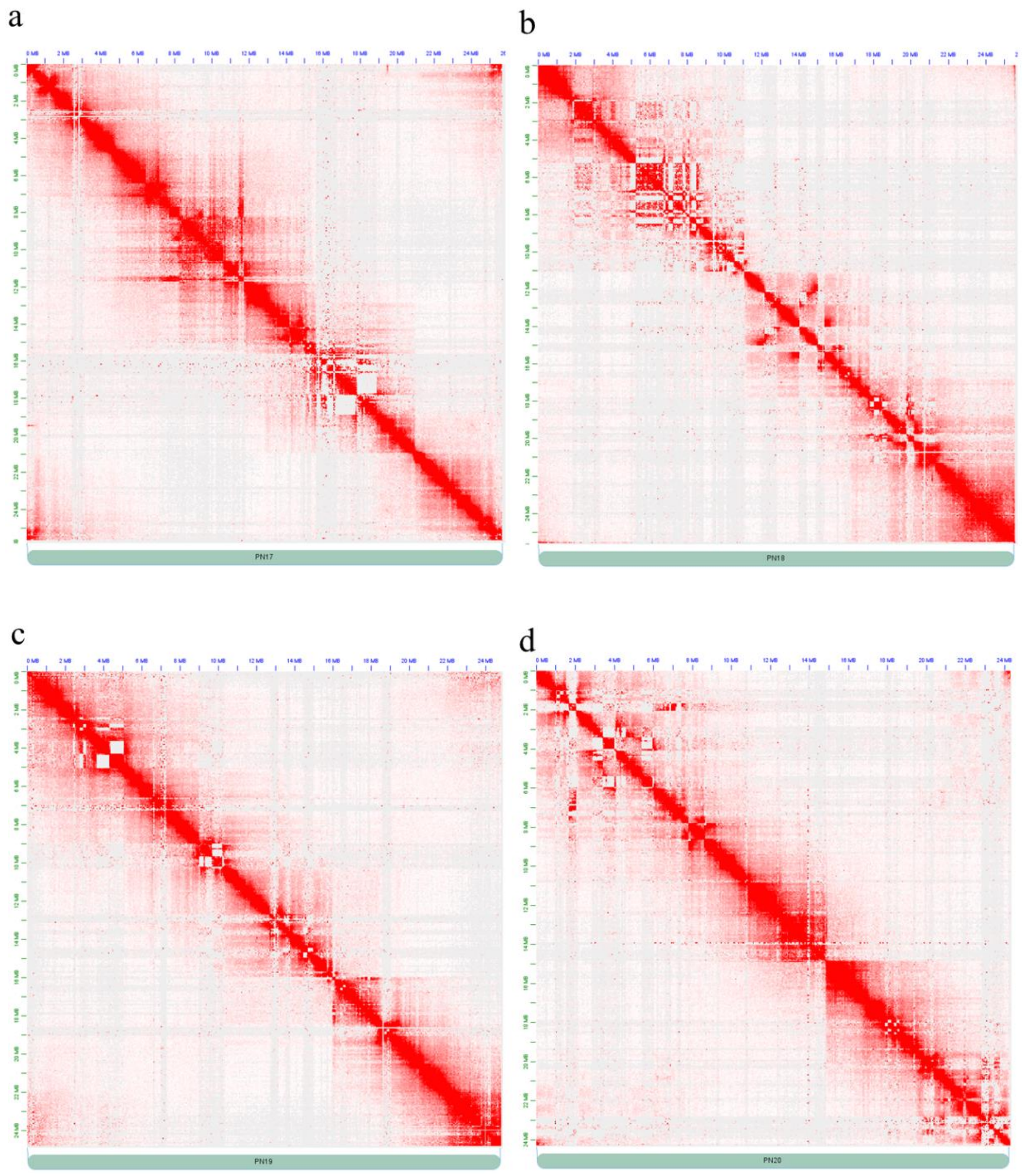

Supplementary Figure 12. Chromatin interactions in chromosome 17 to 20 of black pepper. (a-d) Each heatmap shows the observed values at a resolution of 100 Kb and normalization with balancing.

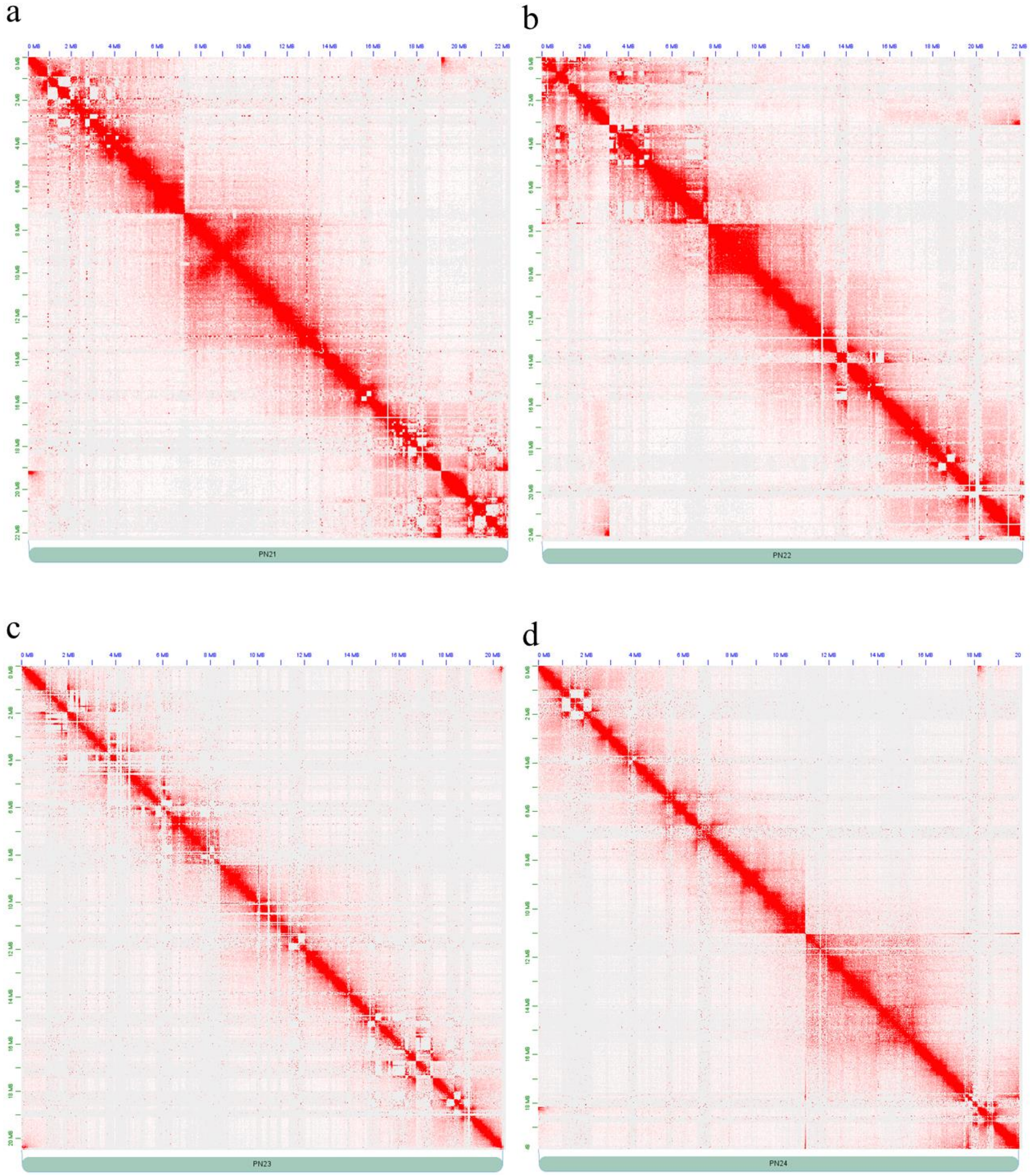

Supplementary Figure 13. Chromatin interactions in chromosome 21 to 24 of black pepper. (a-d) Each heatmap shows the observed values at a resolution of 100 Kb and normalization with balancing.

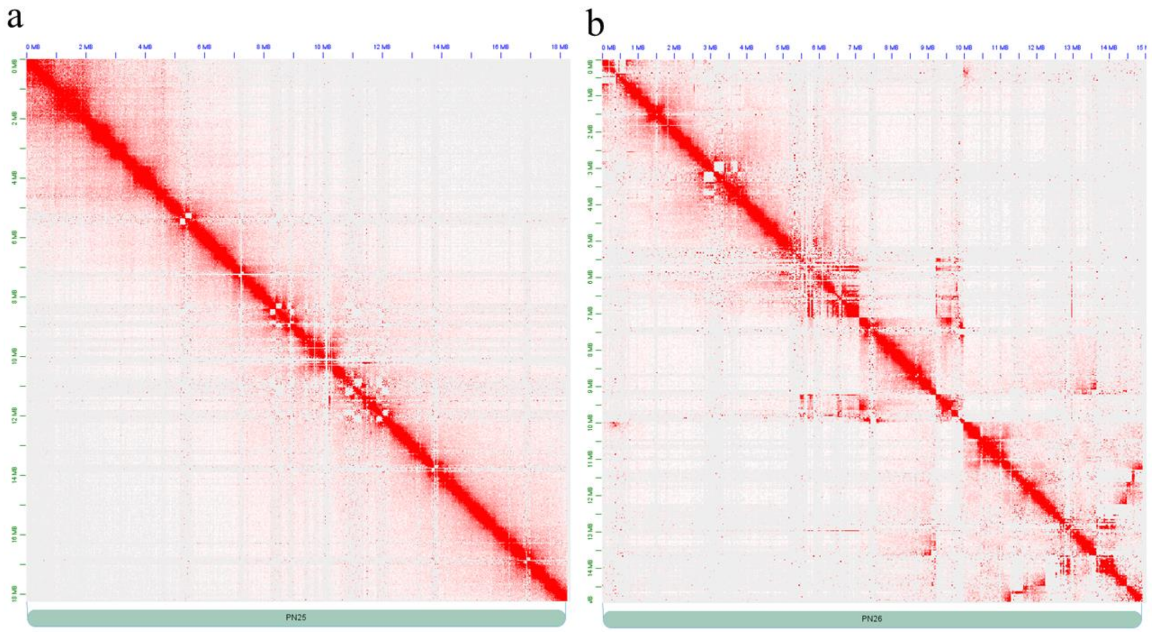

Supplementary Figure 14. Chromatin interactions in chromosome 25 and 26 of black pepper. (a-b) Each heatmap shows the observed values at a resolution of 100 Kb and normalization with balancing.

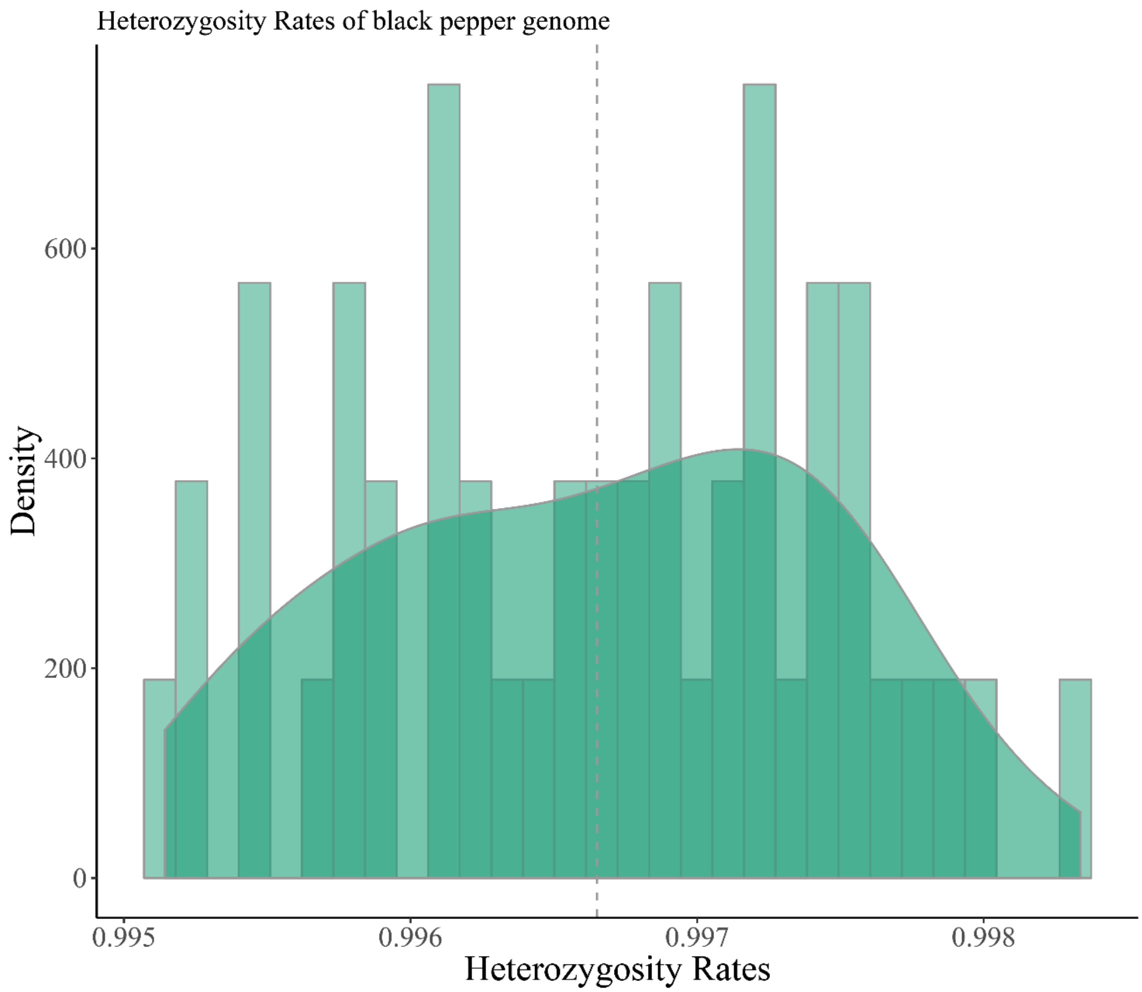

Supplementary Figure 15. Heterozygosity rates of the black pepper genome based on SNP calling. The dashed line indicates the mean value of the heterozygosity rates.

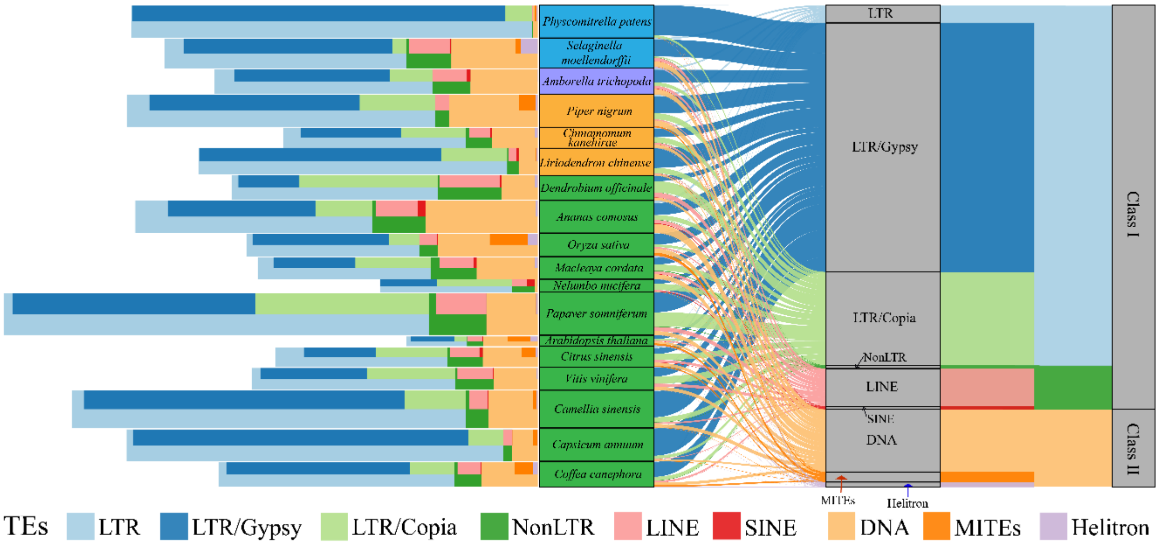

Supplementary Figure 16. Distribution of TEs in species analysed in this study. The size of the bars and flows indicates the percentage of base pairs present in TEs in the genomic sequence. Retrotransposons (Class I) are shown in shades of cyan, and DNA transposons (Class II) are shown in shades of blue. The relative frequency as percentages of Gypsy, Copia, LINE, SINE, MITEs, Helitron, and unclassified LTR, NonLTR, DNA are represented in different colours. The species order is consistent with the species tree of black pepper.

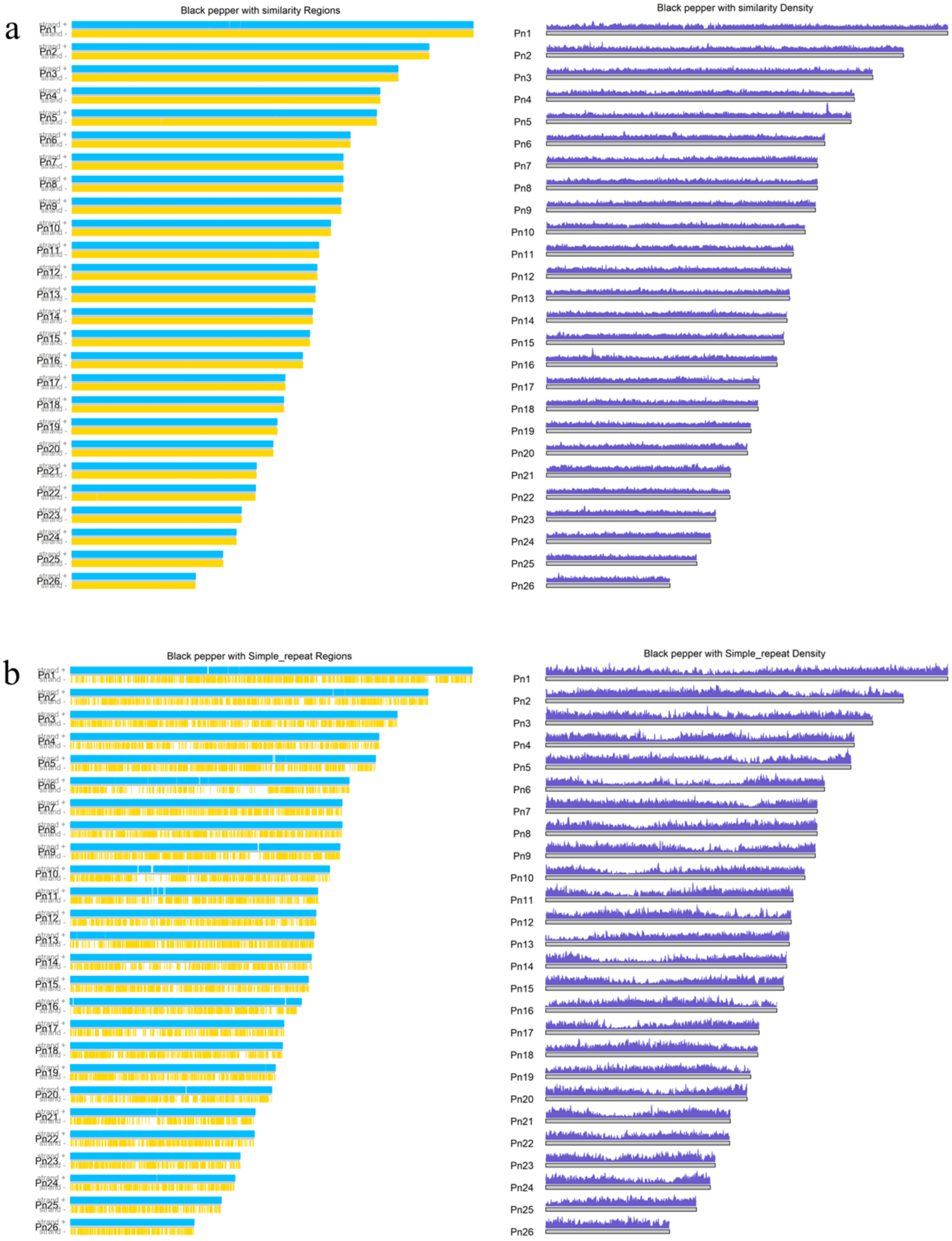

Supplementary Figure 17. Repeat regions and density of the black pepper genome.

(a) Repeat regions and density of all repeat sequences in the black pepper genome. (b) Repeat regions and density of simple repeat sequences in the black pepper genome.

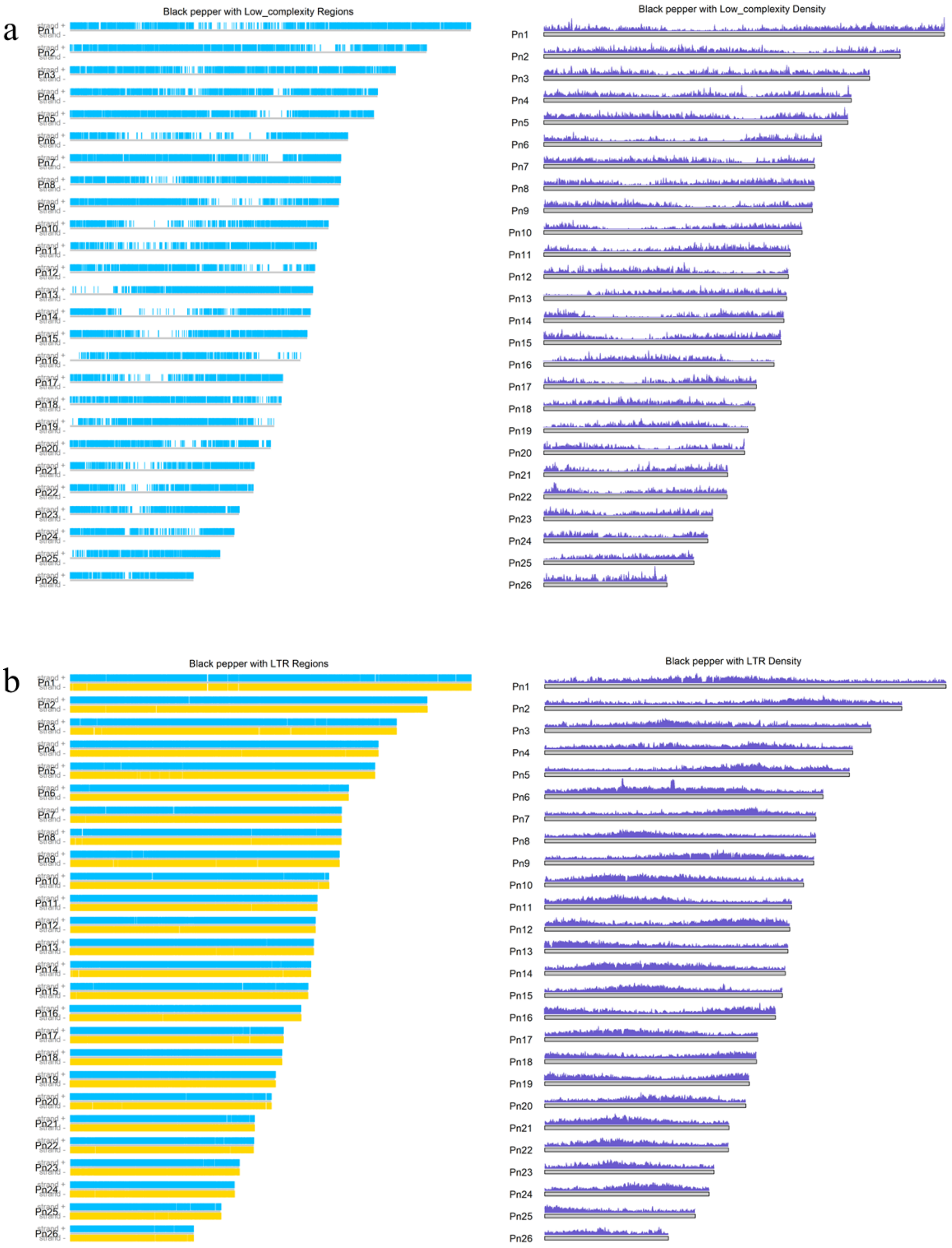

Supplementary Figure 18. Repeat regions and density of the black pepper genome. (a) Repeat regions and density of low complexity repeat sequences in the black pepper genome. (b) Repeat regions and density of LTR sequences in the black pepper genome.

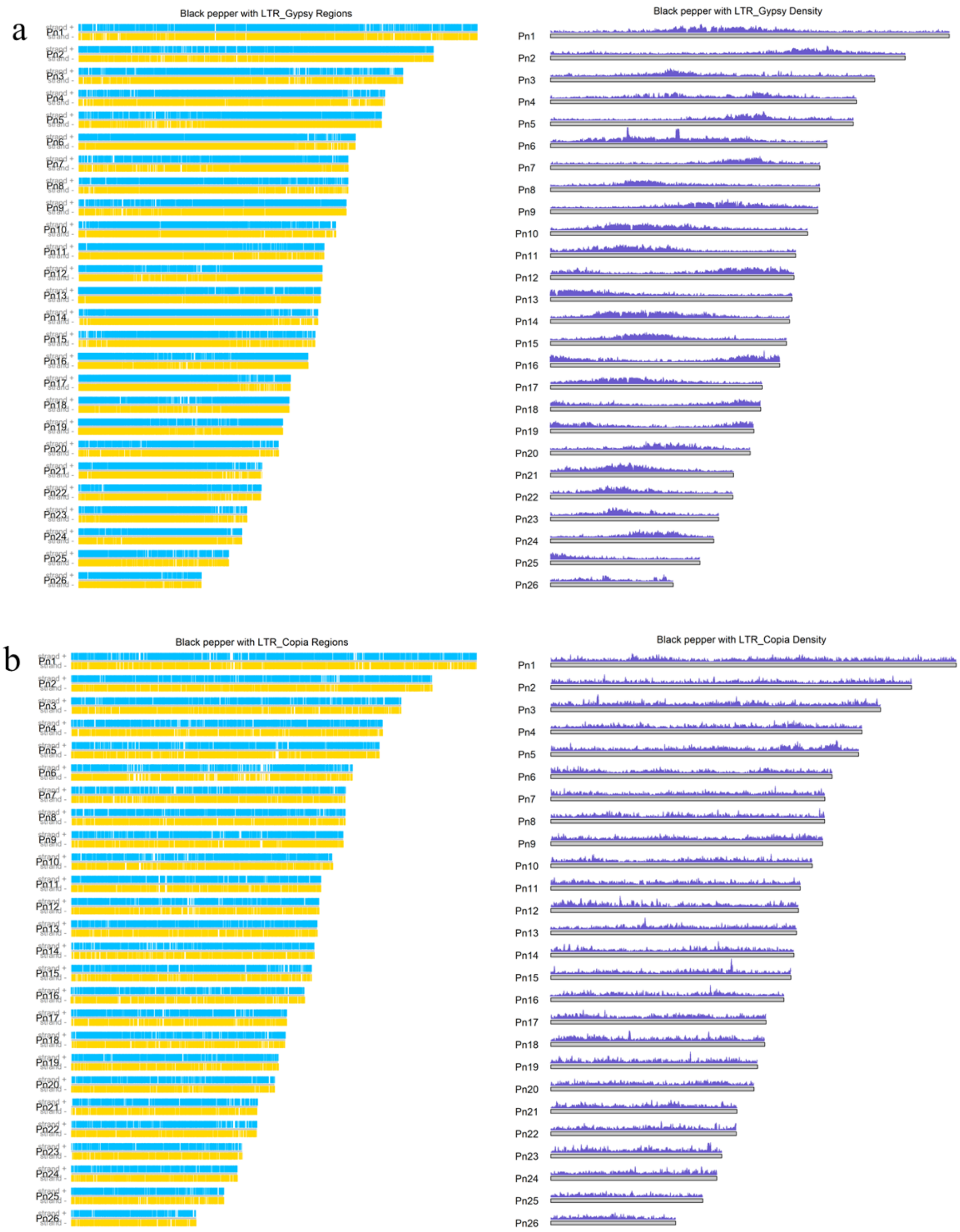

Supplementary Figure 19. Repeat regions and density of the black pepper genome. (a) Repeat regions and density of LTR/Gypsy repeat sequences in the black pepper genome. (b) Repeat regions and density of LTR/Copia repeat sequences in the black pepper genome.

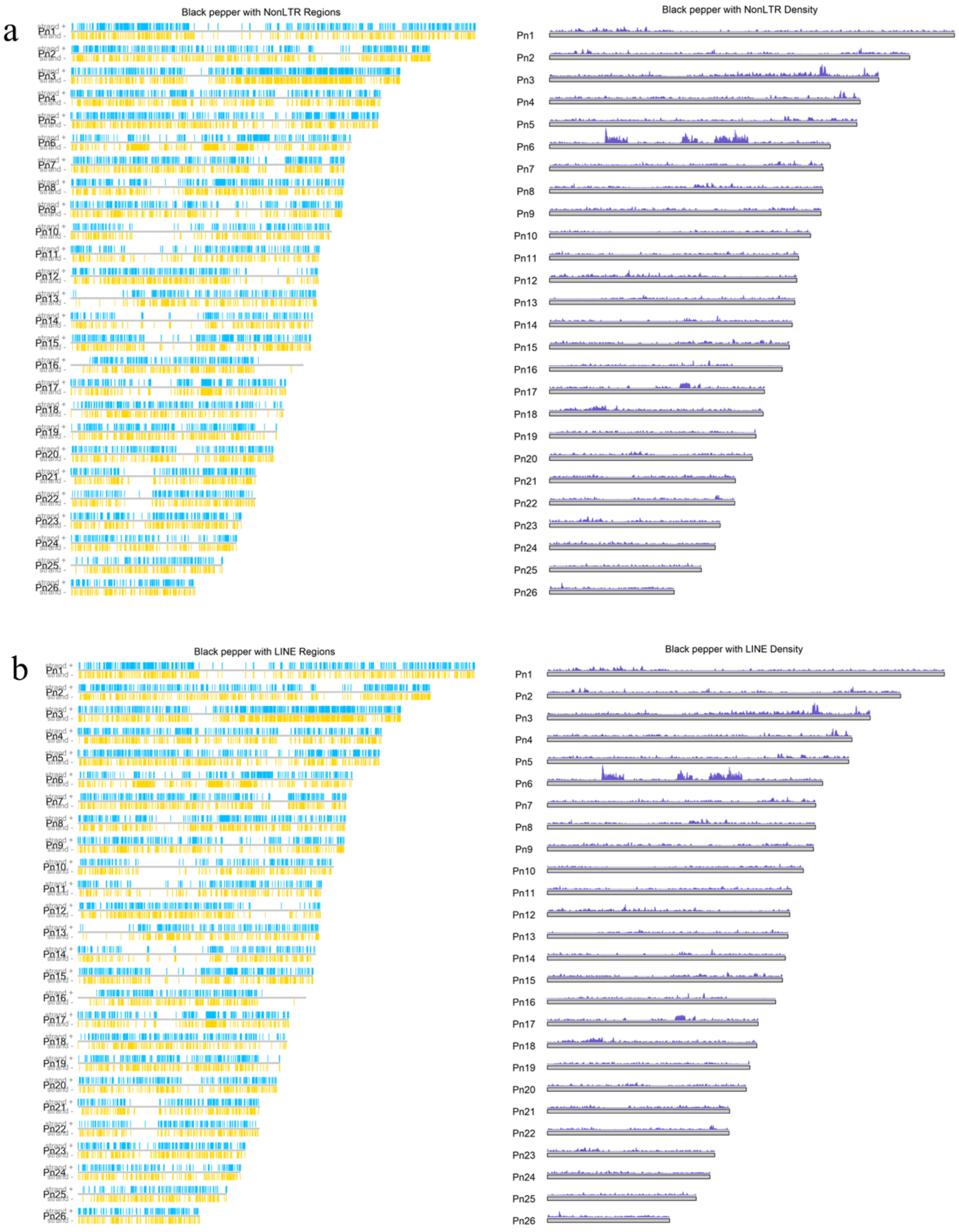

Supplementary Figure 20. Repeat regions and density of the black pepper genome.

(a) Repeat regions and density of NonLTR repeat sequences in the black pepper genome.

(b) Repeat regions and density of LINE repeat sequences in the black pepper genome.

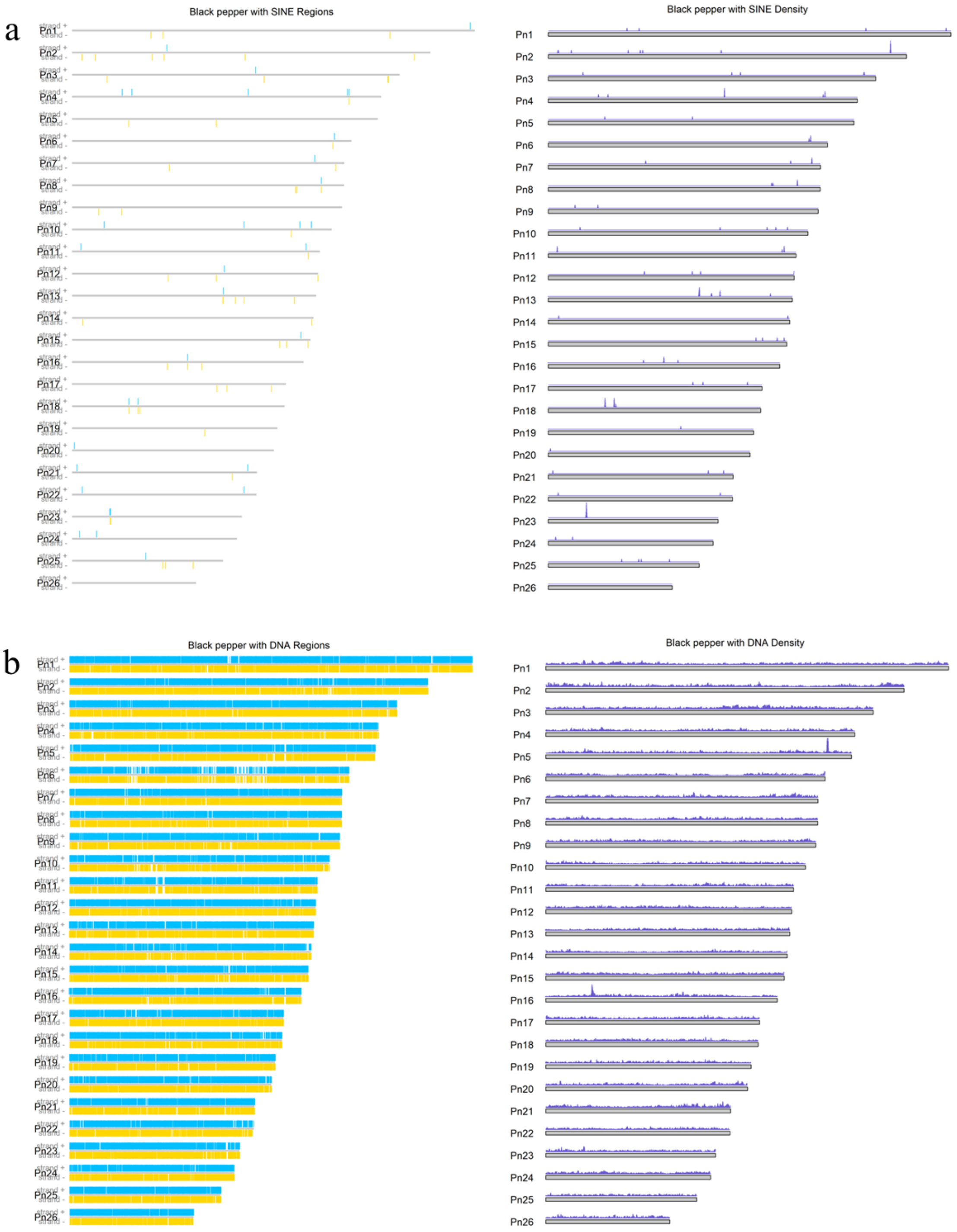

Supplementary Figure 21. Repeat regions and density of the black pepper genome. (a) Repeat regions and density of SINE repeat sequences in the black pepper genome. (b) Repeat regions and density of DNA transposons in the black pepper genome.

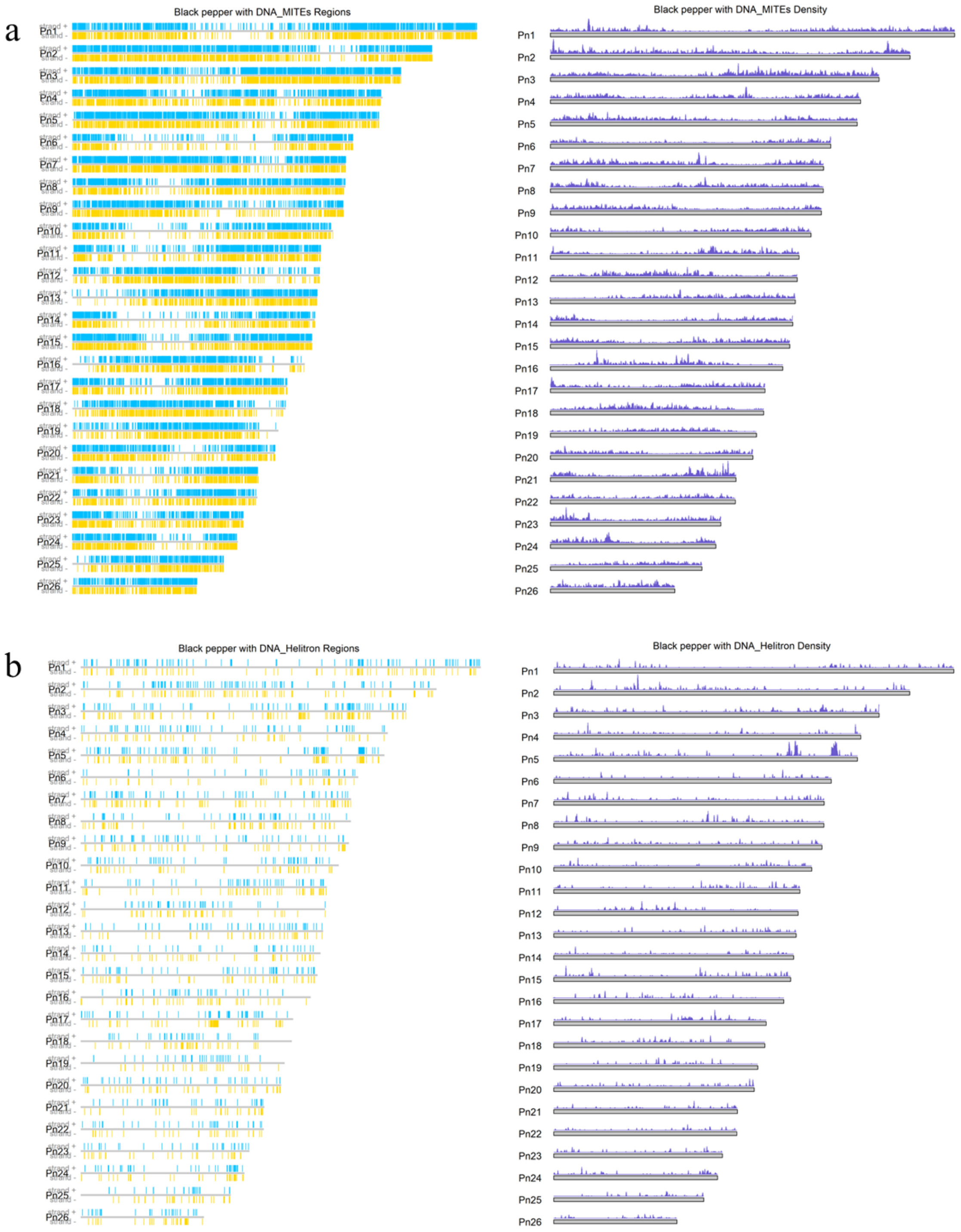

Supplementary Figure 22. Repeat regions and density of the black pepper genome. (a) Repeat regions and density of MITE repeat sequences in the black pepper genome. (b) Repeat regions and density of Helitron repeat sequences in the black pepper genome.

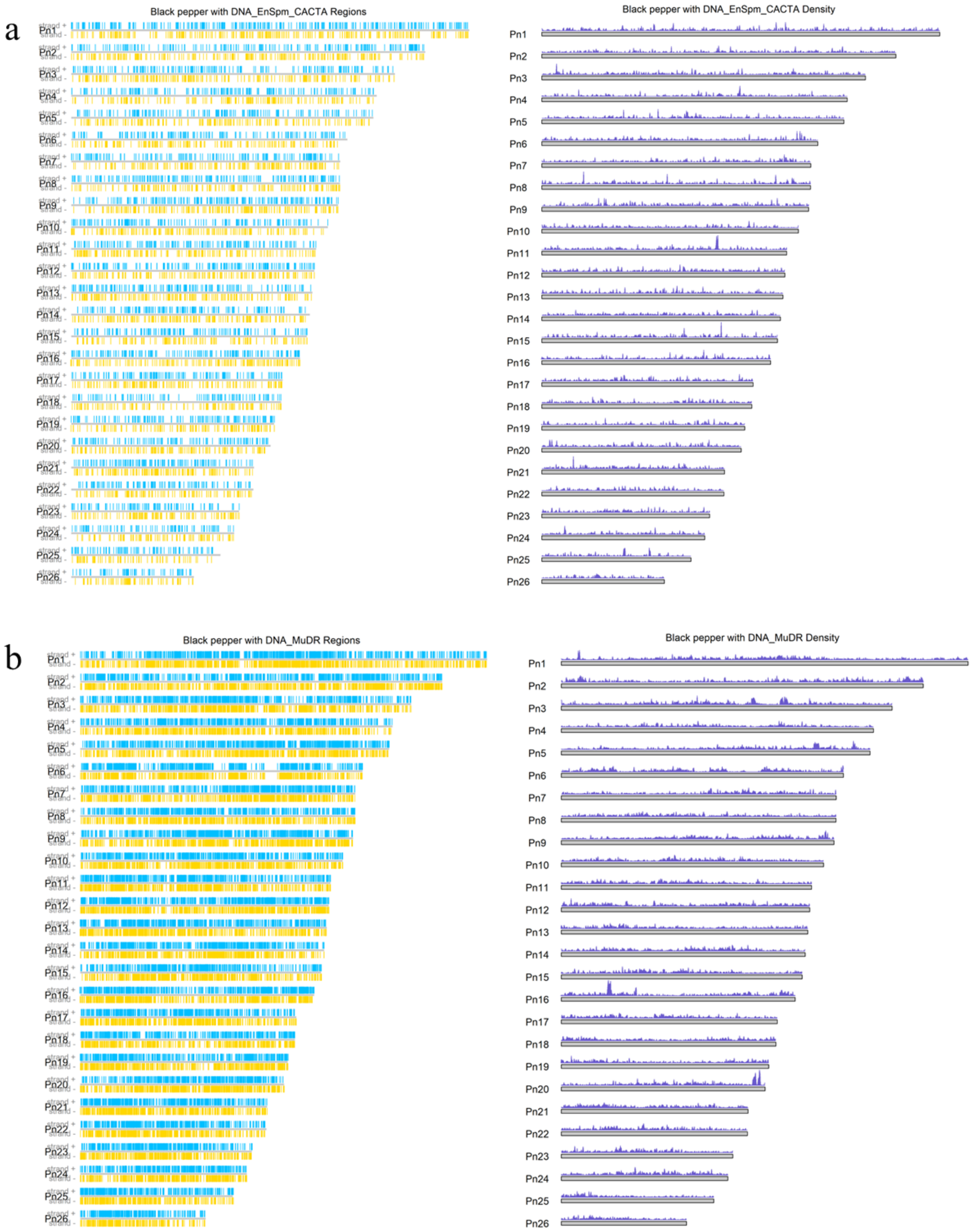

Supplementary Figure 23. Repeat regions and density of the black pepper genome. (a) Repeat regions and density of EnSpm/CACTA repeat sequences in the black pepper genome. (b) Repeat regions and density of MuDR repeat sequences in the black pepper genome.

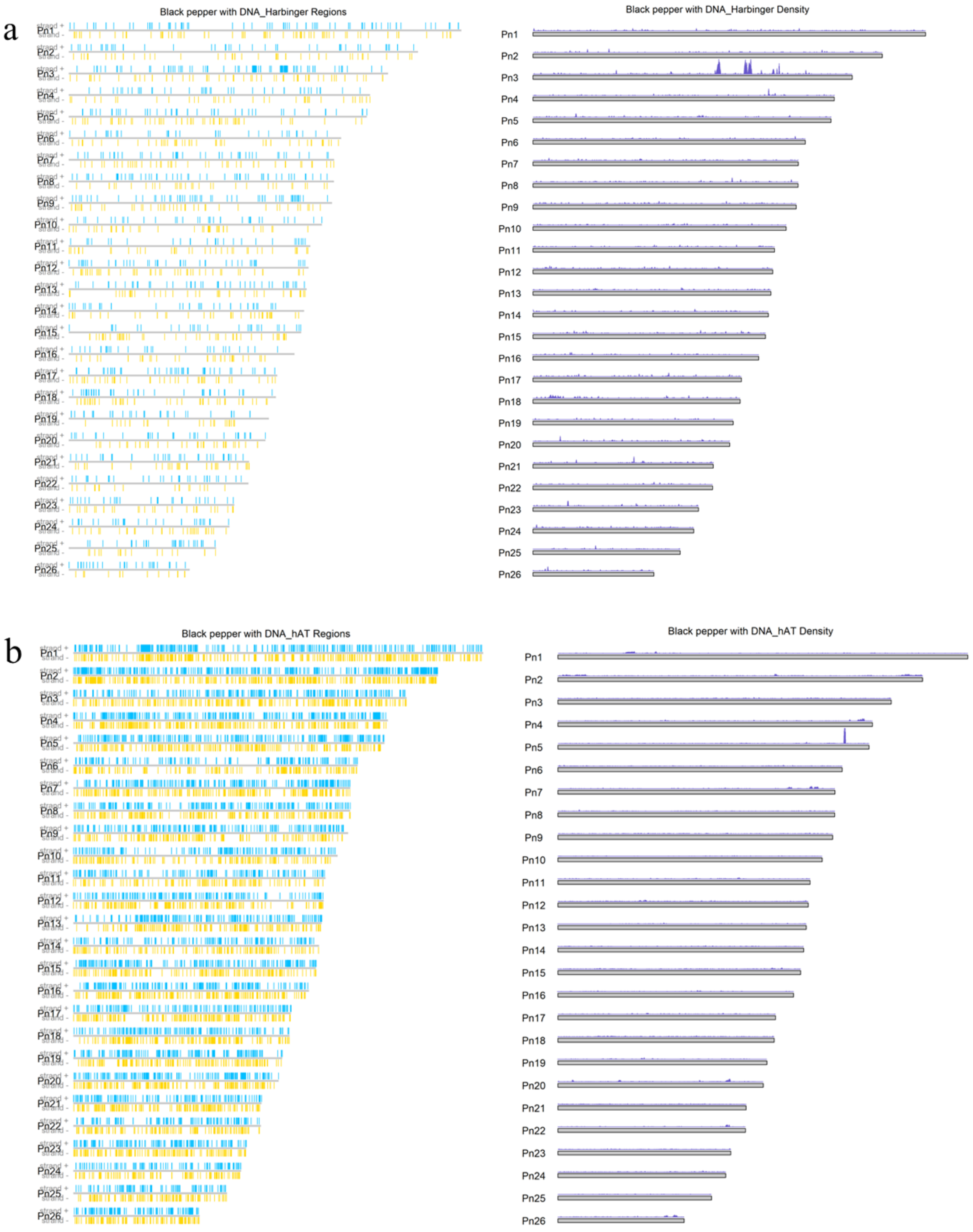

Supplementary Figure 24. Repeat regions and density of the black pepper genome.

(a) Repeat regions and density of Harbinger repeat sequences in the black pepper genome.

(b) Repeat regions and density of hAT repeat sequences in the black pepper genome.

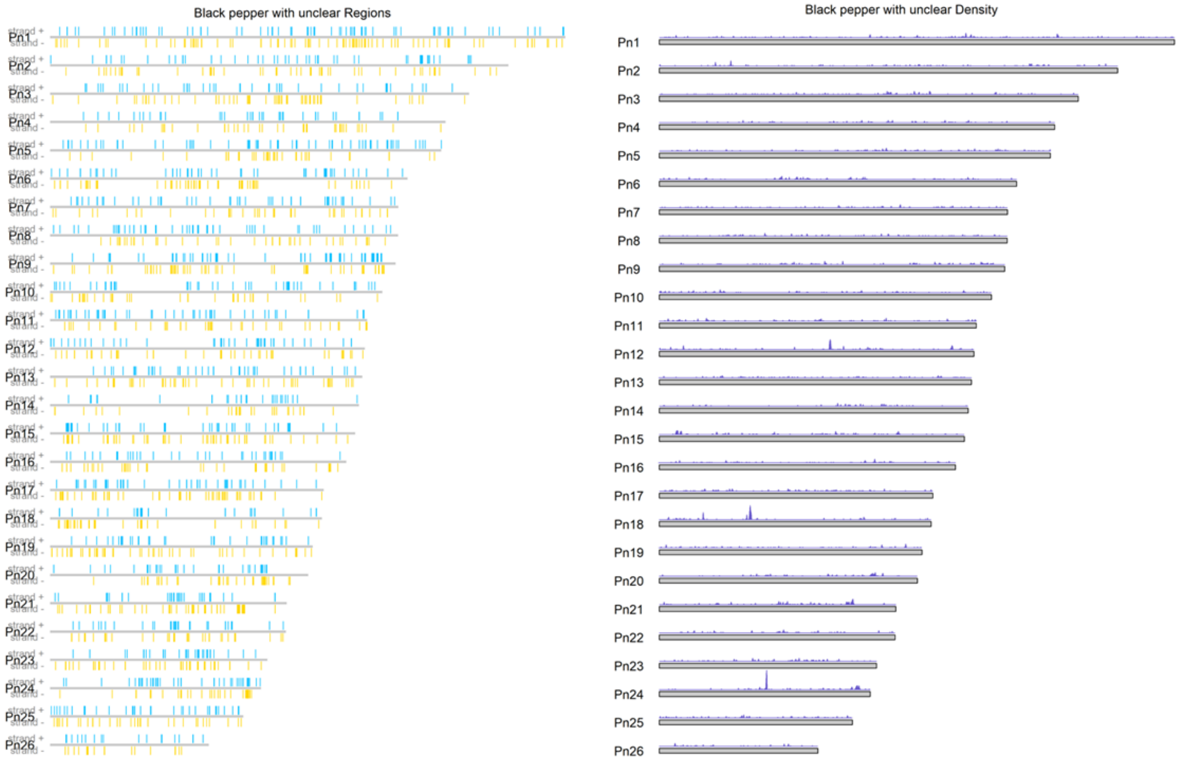

Supplementary Figure 25. Repeat regions and density of the black pepper genome.

Repeat regions and density of unclear repeat sequences in the black pepper genome.

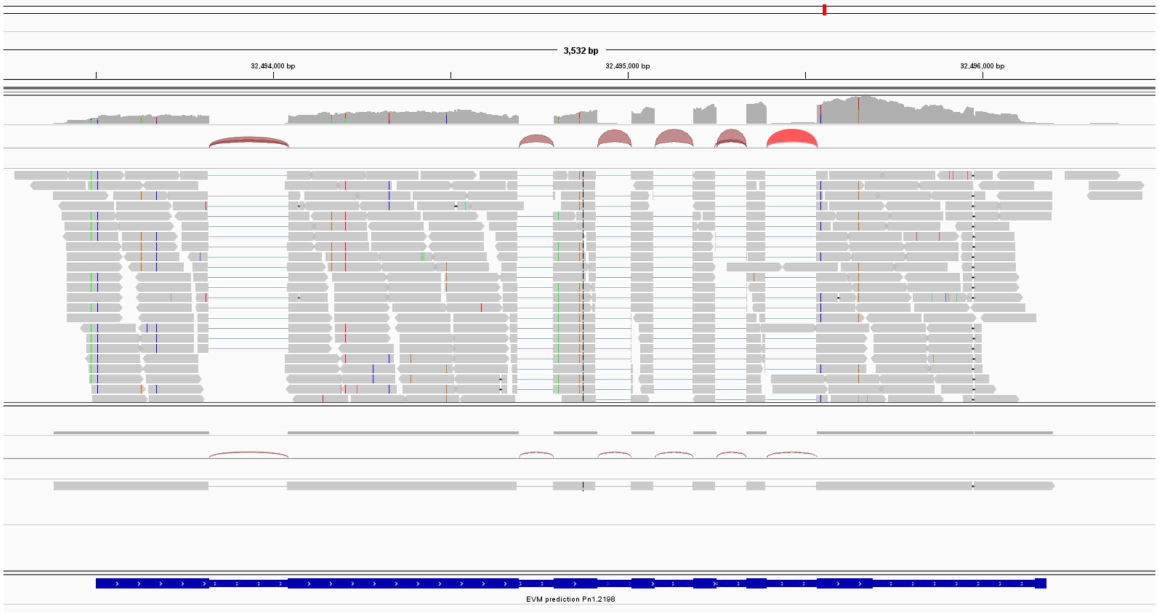

Supplementary Figure 26. Black pepper genome annotation. Representative gene model showing mapped RNA sequencing reads generated using Illumina or PacBio Iso-Seq sequencing technologies. The top and middle panels show RNA-seq reads and PacBio Iso-Seq sequencing, respectively, mapped to the chromosomal location containing the Pn1.2198 gene model, which is shown in the bottom panel.

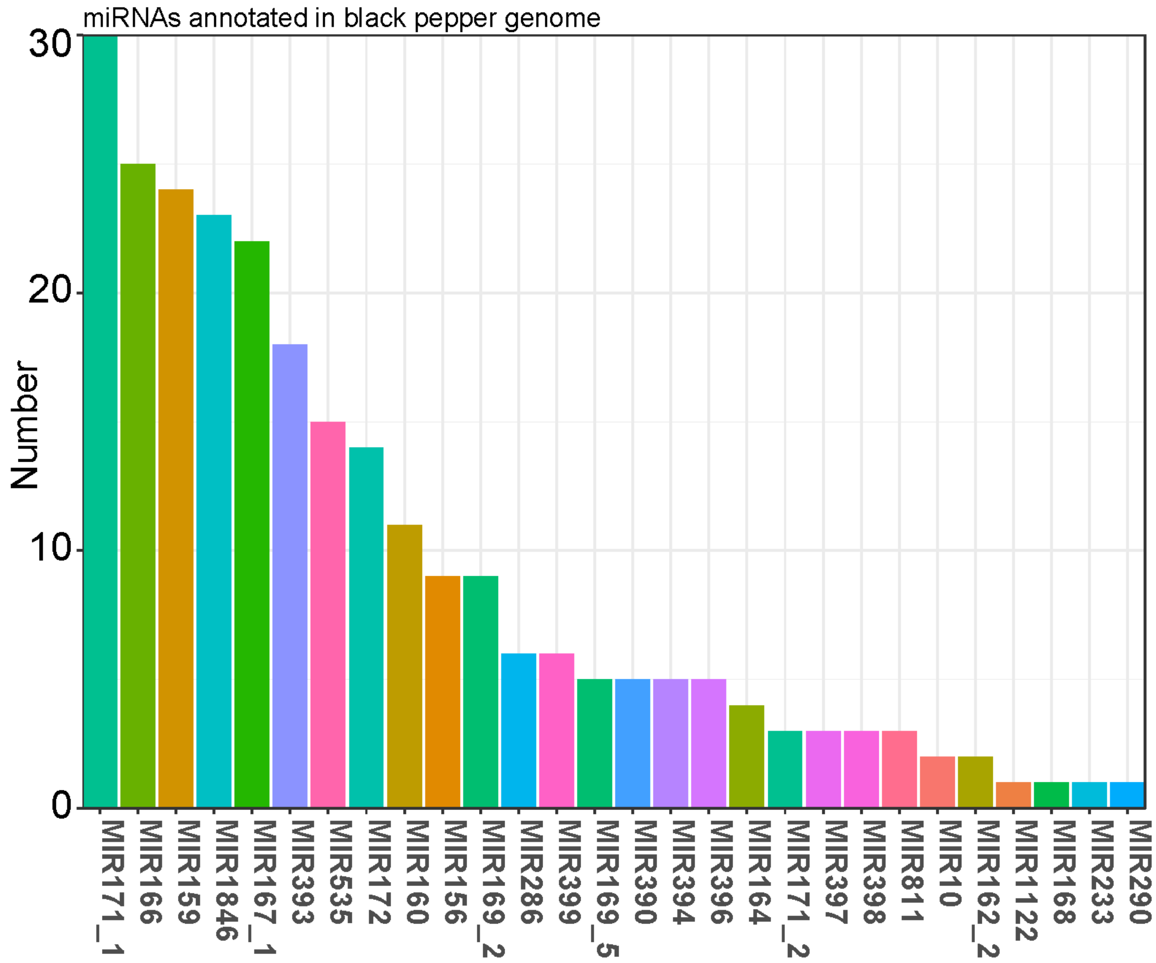

Supplementary Figure 27. Distribution of annotated miRNAs in the black pepper genome.

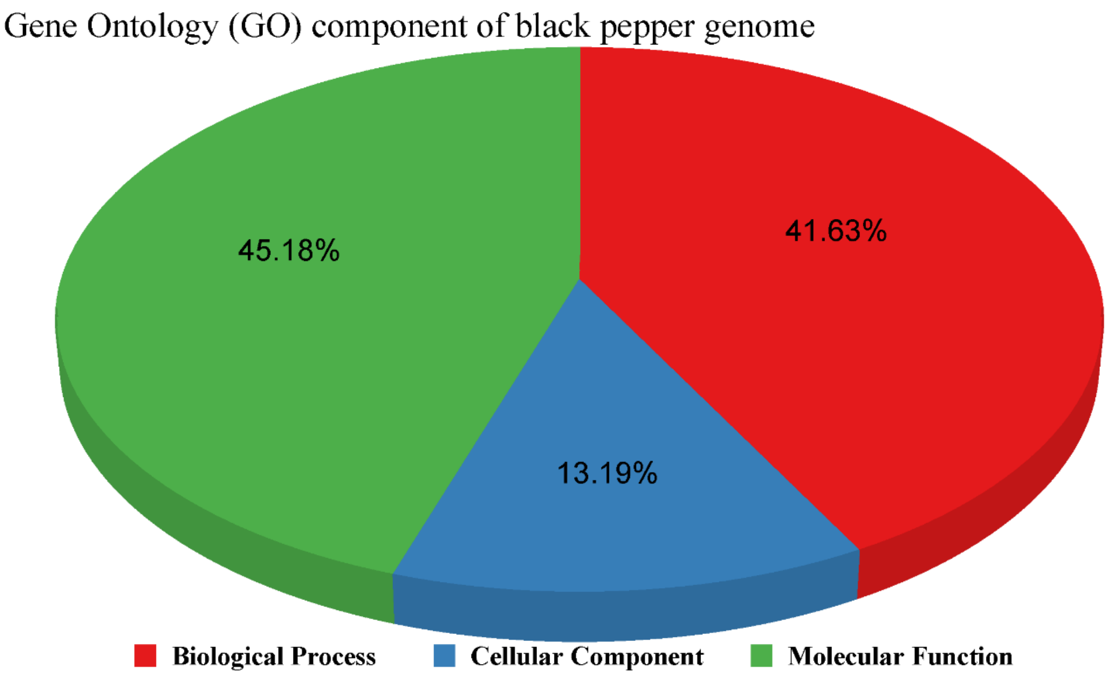

Supplementary Figure 28. Gene Ontology distribution of annotated genes in the black pepper genome.

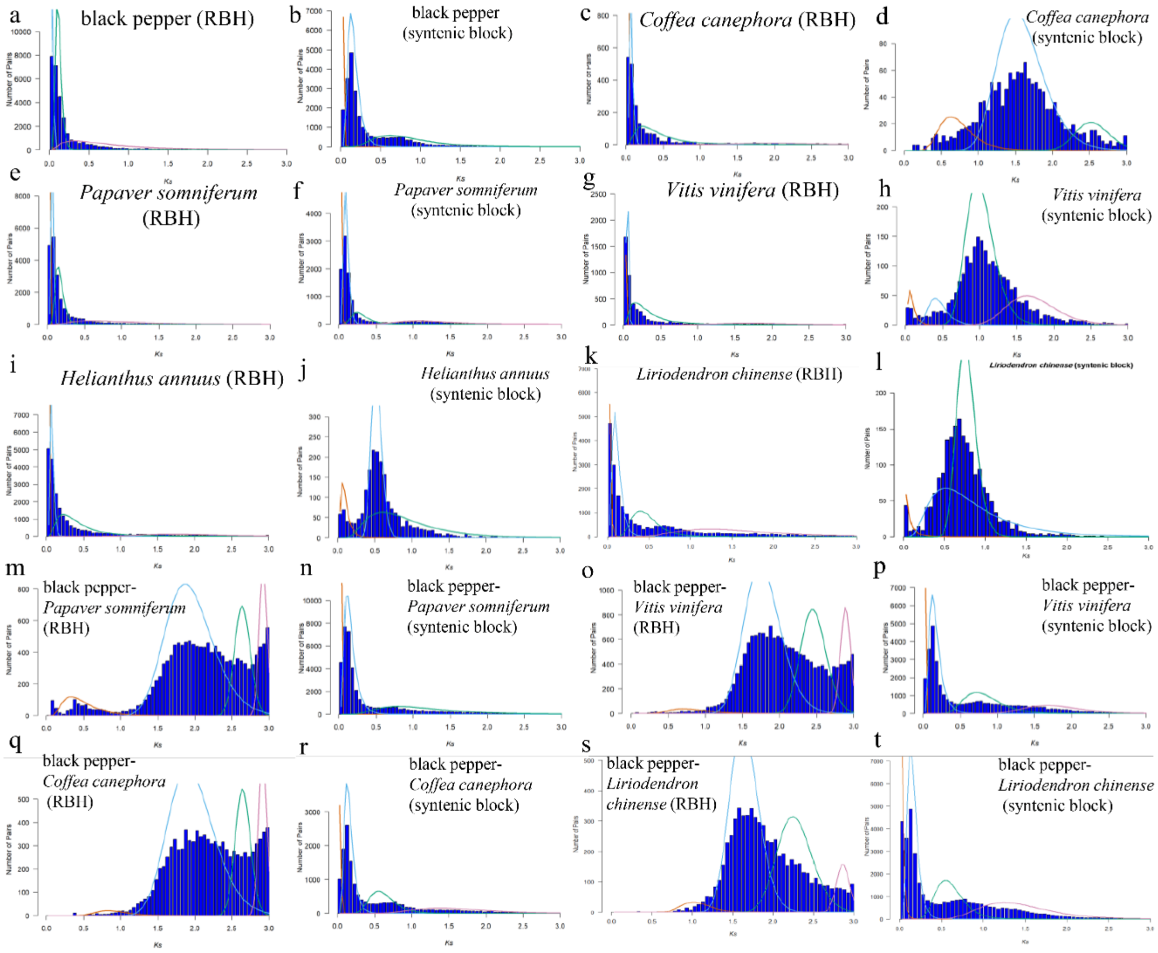

Supplementary Figure 29. Histogram distribution of synonymous substitution rate for homologous gene pairs. (a, c, e, g, i, k, m, o, q and s) Identified using the reciprocal best hit (RBH) analysis. (b, d, f, h, j, l, n, p, r and t) Syntenic block gene pairs identified with MCScanX analysis.

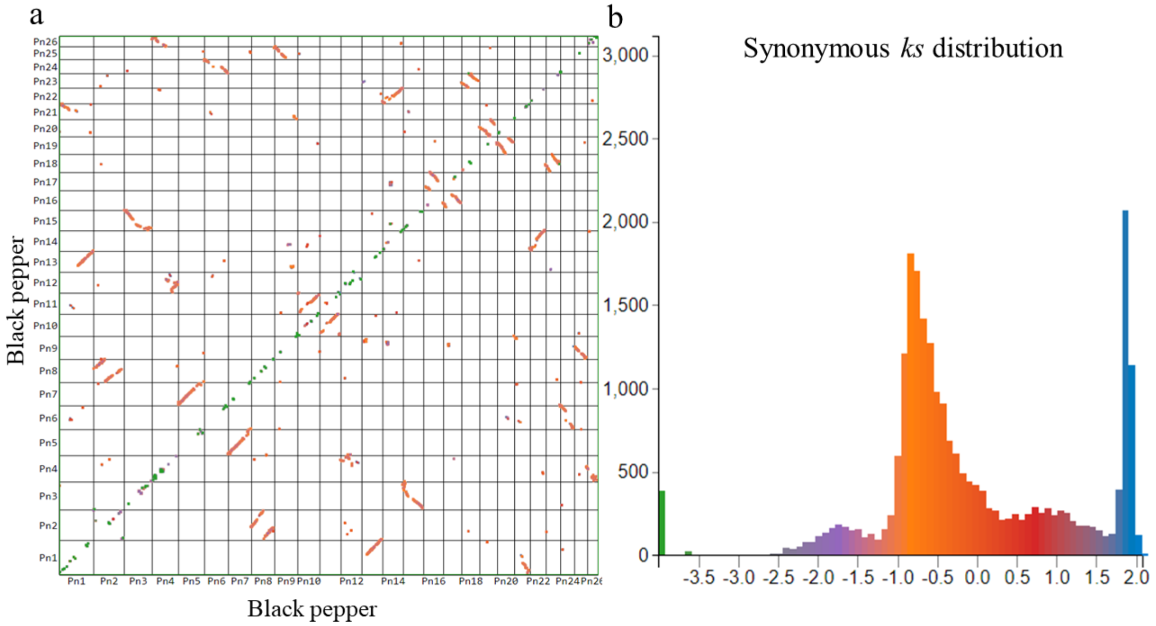

Supplementary Figure 30. Synteny analysis within the black pepper genome. (a) Dot plot matrix displaying the paralogs in black pepper. (b) Synonymous K_s distribution of paralogs genes in the black pepper genome.

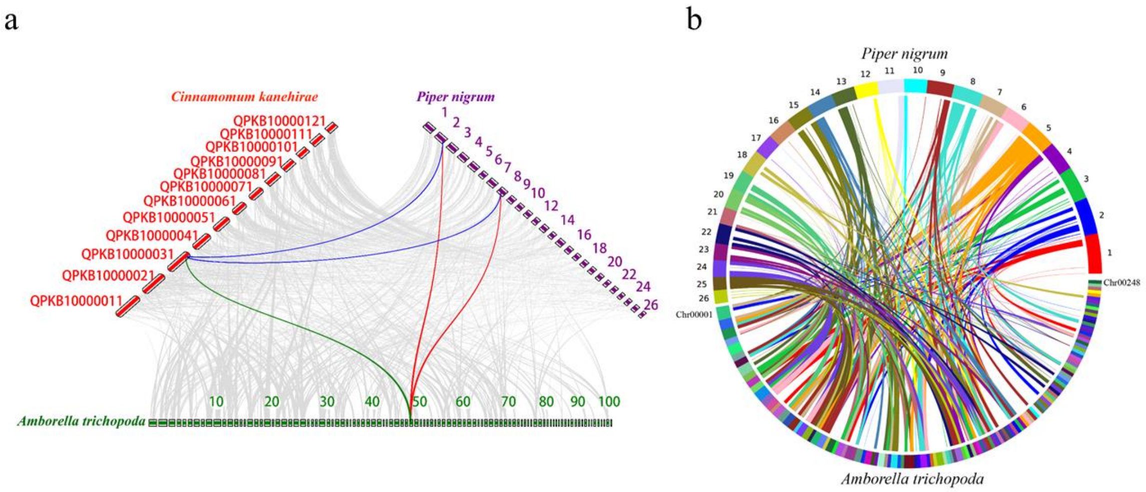

Supplementary Figure 31. Synteny analysis of black pepper. (a) Macrosynteny patterns show that a typical ancestral region in the basal angiosperm *Amborella* can be tracked to up to two regions in black pepper and to up to one region in *Cinnamomum micranthum*. Grey wedges in the background highlight major syntenic blocks spanning the genomes (highlighted by one syntenic set shown in colour). (b) Synteny of black pepper and *Amborella trichopoda* genomes.

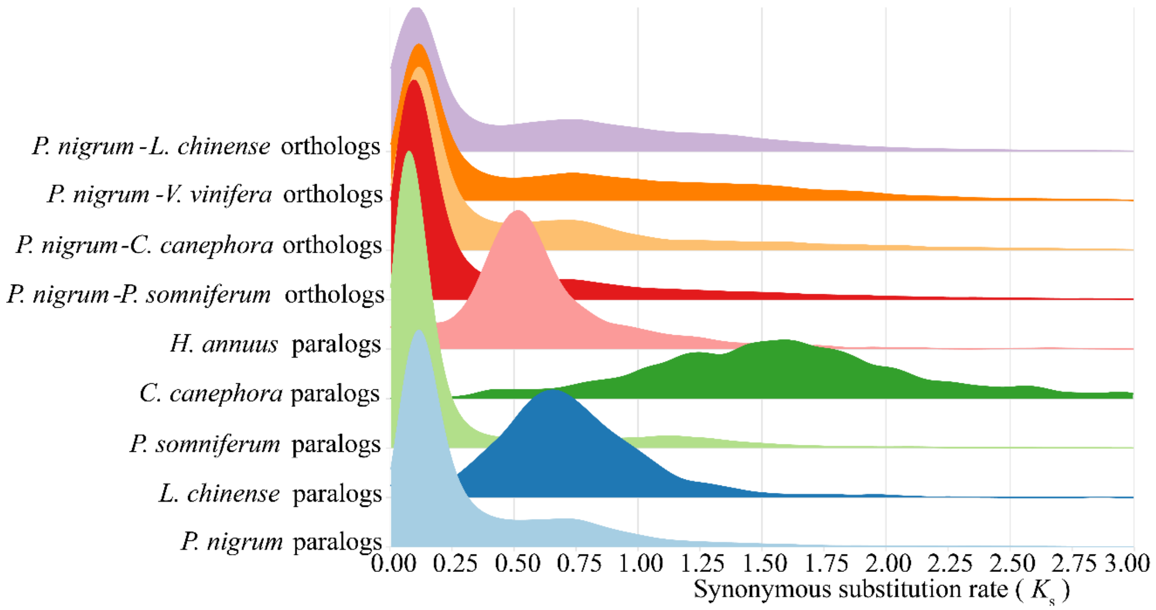

Supplementary Figure 32. Synonymous substitution rate distribution of syntenic block gene pair. All is identified using MCScanX analysis in different species.

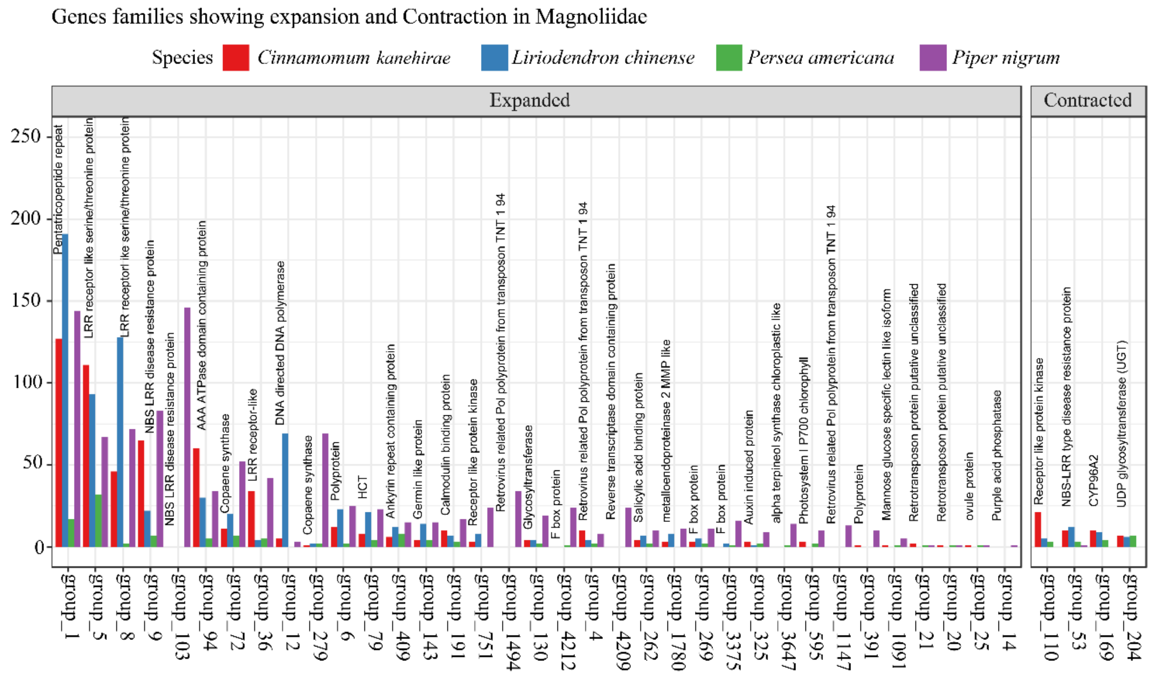

Supplementary Figure 33. Gene family expansion and contraction in the Magnoliidae. The text over the bar indicates the function of corresponding gene family.

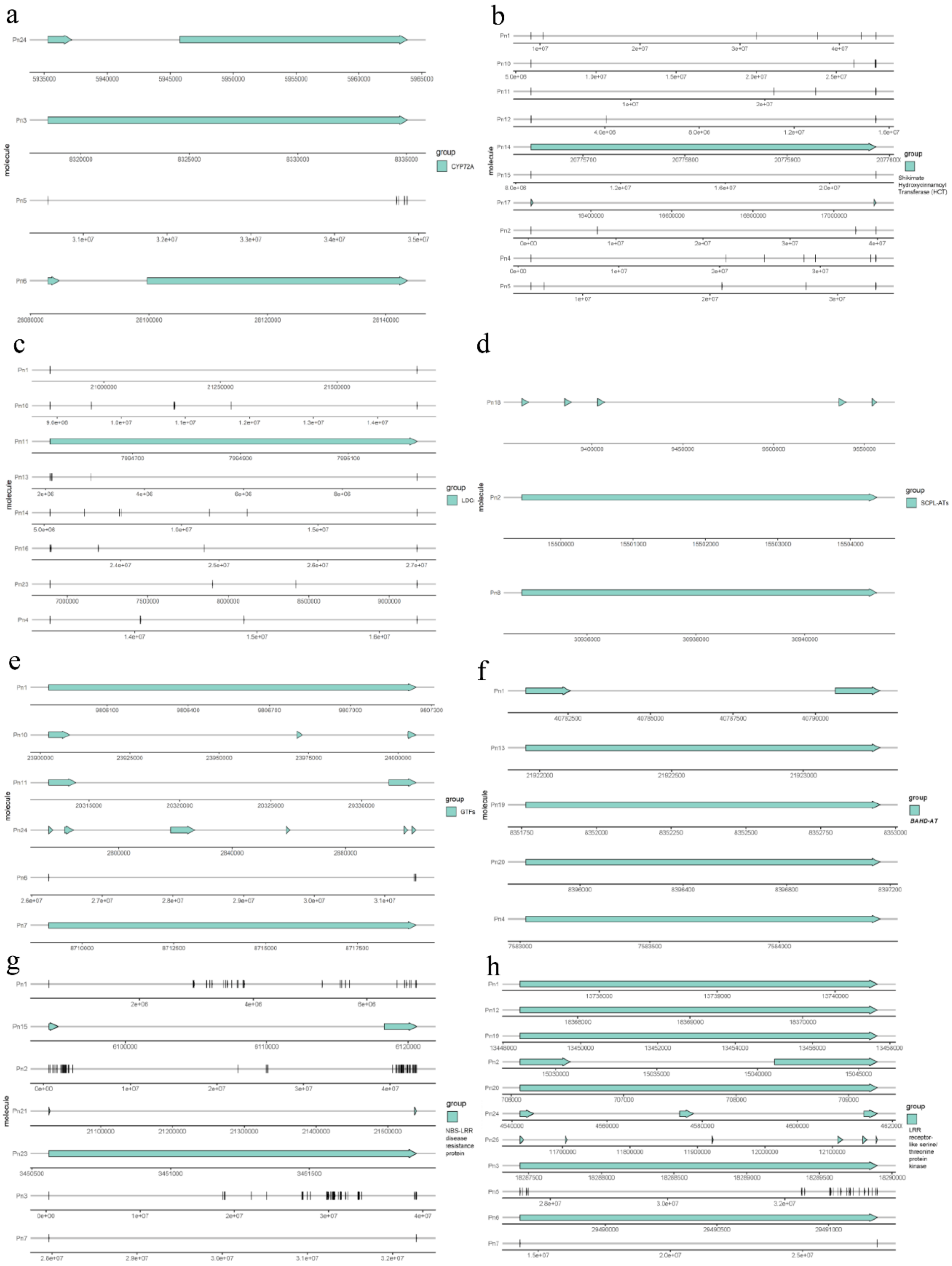
a
b
c
d
e
f
g
h
molecule
group
CYP72A
Shikimate Hydroxycinnamoyl Transferase (HCT)
SCPL-ATs
GTFs
BAHD-AT
NBS-LRR disease resistance protein
LRR receptor-like serine/threonine protein kinase

Supplementary Figure 34. Arrangement and chromosomal position of expanded genes in black pepper**.** (a-f) Secondary metabolism-associated genes. and (g and h) Disease resistance-associated genes.

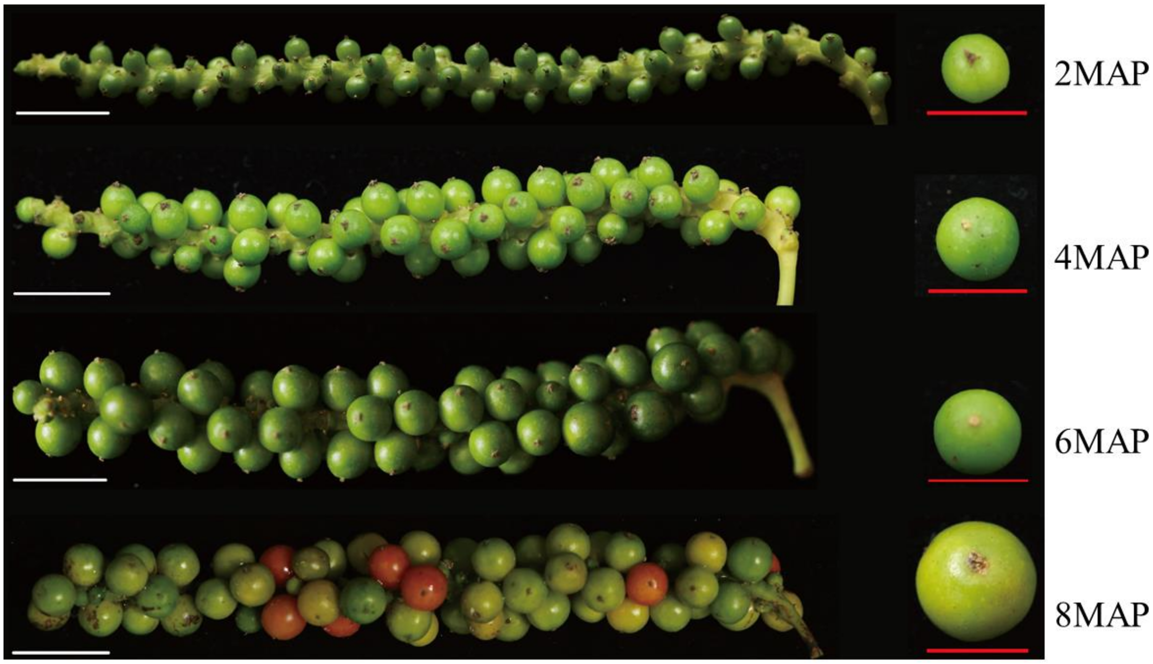

Supplementary Figure 35. Picture of black pepper berry at different developmental stages. White bar = 1 cm and red bar = 0.5 cm. All the images are taken from black pepper that was sequenced, and was grown at the Spice and Beverage Research Institute, Chinese Academy of Tropical Agricultural Sciences.

a

Sample Name	% Dups	% GC	Length	% Failed	M Seqs
PN_FL_1	37.2%	48%	150 bp	27%	24.1
PN_FL_2	30.3%	48%	150 bp	27%	24.1
PN_FL_29_1	61.6%	47%	150 bp	36%	28.2
PN_FL_29_2	61.1%	47%	150 bp	36%	28.2
PN_FL_30_1	58.6%	47%	150 bp	36%	22.9
PN_FL_30_2	58.1%	47%	150 bp	36%	22.9
PN_FR_11_1	47.7%	48%	150 bp	27%	26.5
PN_FR_11_2	47.2%	48%	150 bp	27%	26.5
PN_FR_12_1	46.6%	48%	150 bp	27%	26.4
PN_FR_12_2	46.2%	48%	150 bp	27%	26.4
PN_FR_1_1	38.9%	48%	150 bp	27%	31.1
PN_FR_1_2	33.6%	48%	150 bp	27%	31.1
PN_FR_21_1	52.5%	48%	150 bp	36%	26.2
PN_FR_21_2	52.1%	48%	150 bp	36%	26.2
PN_FR_22_1	53.5%	48%	150 bp	36%	31.6
PN_FR_22_2	52.9%	48%	150 bp	36%	31.6
PN_FR_2_1	36.8%	48%	150 bp	27%	22.4
PN_FR_2_2	27.6%	48%	150 bp	27%	22.4
PN_FR_31_1	51.5%	49%	150 bp	36%	26.2
PN_FR_31_2	51.1%	49%	150 bp	36%	26.2
PN_FR_32_1	51.7%	49%	150 bp	36%	24.1
PN_FR_32_2	51.2%	49%	150 bp	36%	24.1
PN_FR_3_1	36.0%	47%	150 bp	27%	19.7
PN_FR_3_2	31.6%	47%	150 bp	27%	19.7
PN_FR_41_1	52.9%	48%	150 bp	36%	28.3
PN_FR_41_2	52.3%	48%	150 bp	36%	28.3
PN_FR_42_1	47.1%	48%	150 bp	27%	25.6
PN_FR_42_2	46.7%	48%	150 bp	27%	25.6
PN_FR_4_1	39.8%	48%	150 bp	27%	22.4
PN_FR_4_2	34.3%	48%	150 bp	27%	22.4
PN_L_1	37.4%	48%	150 bp	27%	27.2
PN_L_2	33.5%	48%	150 bp	27%	27.2
PN_L_27_1	53.6%	48%	150 bp	36%	29.1
PN_L_27_2	52.9%	48%	150 bp	36%	29.1
PN_L_28_1	51.4%	48%	150 bp	36%	28.0
PN_L_28_2	50.9%	48%	150 bp	36%	28.0
PN_R_1	35.9%	49%	150 bp	27%	23.2
PN_R_2	31.9%	49%	150 bp	27%	23.2
PN_R_23_1	53.5%	47%	150 bp	36%	31.8
PN_R_23_2	53.0%	47%	150 bp	36%	31.8
PN_R_24_1	52.6%	48%	150 bp	36%	27.9
PN_R_24_2	52.2%	48%	150 bp	36%	27.9
PN_SM_1	35.5%	47%	150 bp	27%	26.6
PN_SM_2	29.6%	47%	150 bp	27%	26.6
PN_SM_25_1	52.0%	48%	150 bp	36%	27.3
PN_SM_25_2	51.4%	48%	150 bp	36%	27.3
PN_SM_26_1	50.2%	48%	150 bp	36%	23.4
PN_SM_26_2	49.7%	48%	150 bp	27%	23.4

b

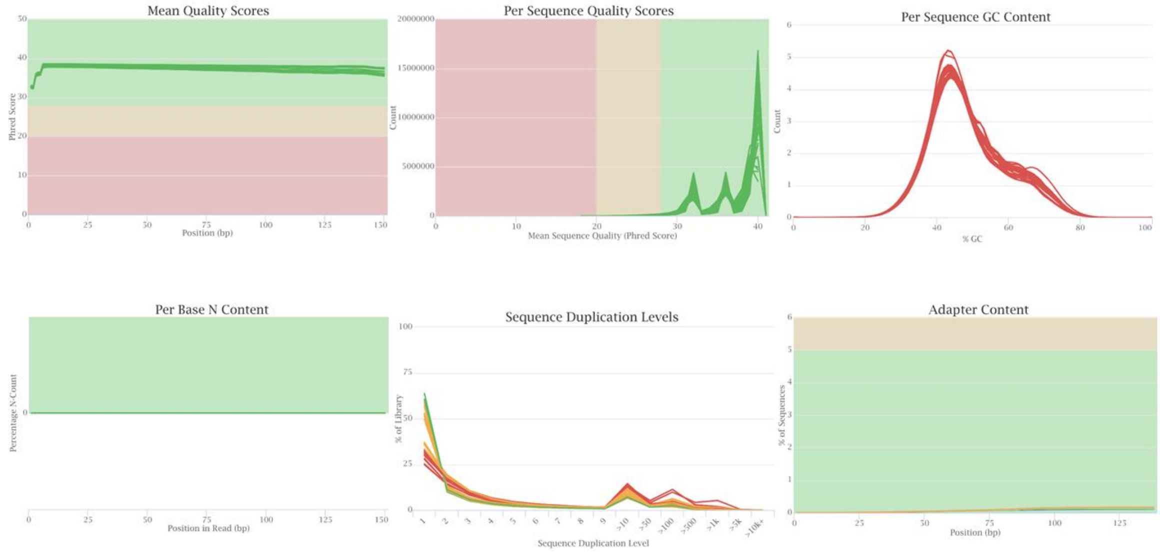

Supplementary Figure 36. **Quality checks of RNA-seq data.** (a) Statistical analysis of duplicate reads, average GC content and total sequences in RNA-seq data obtained from black pepper. (b) Distribution of sequence quality, N content, duplication levels and adapter content in RNA-seq data from black pepper.

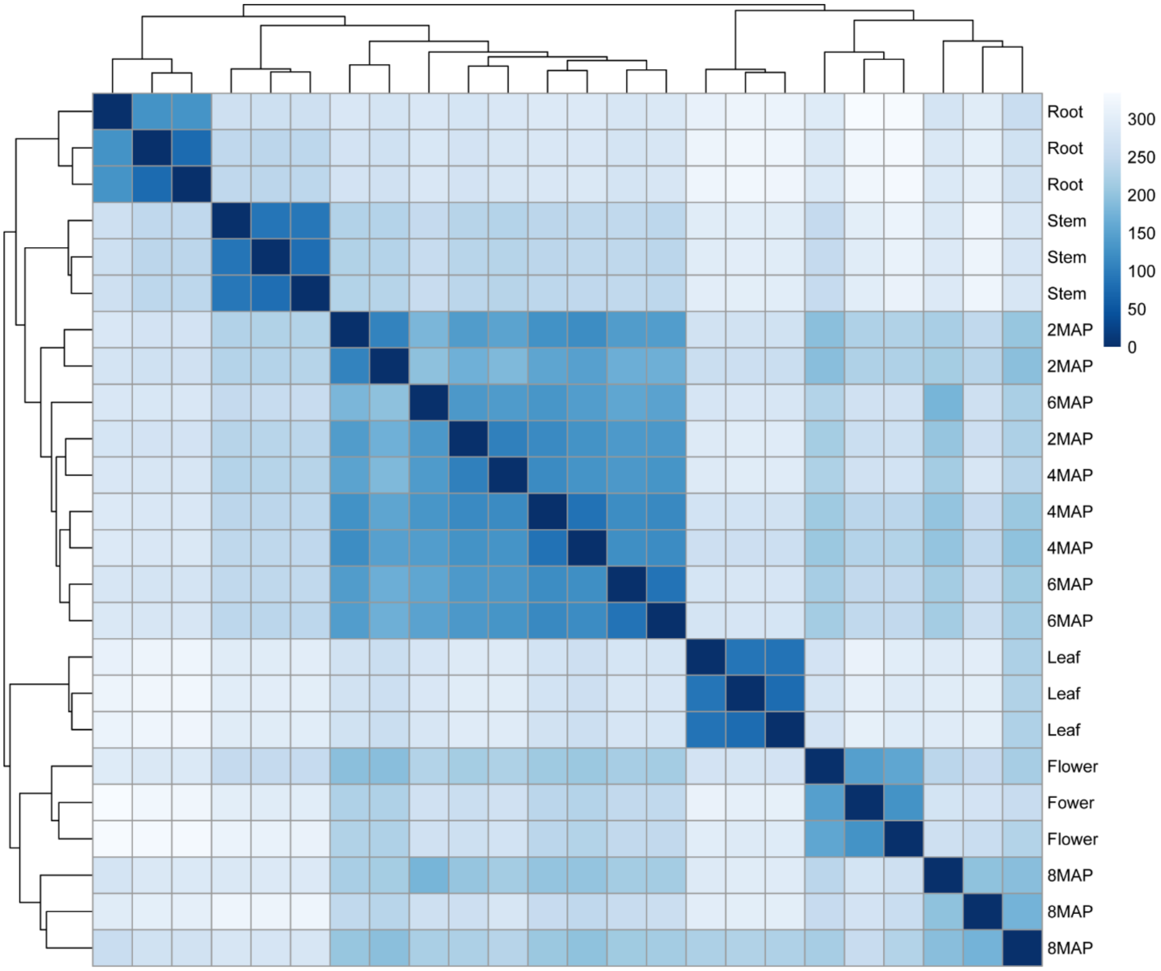

Supplementary Figure 37. Correlation of RNA-seq data in different tissues from black pepper.

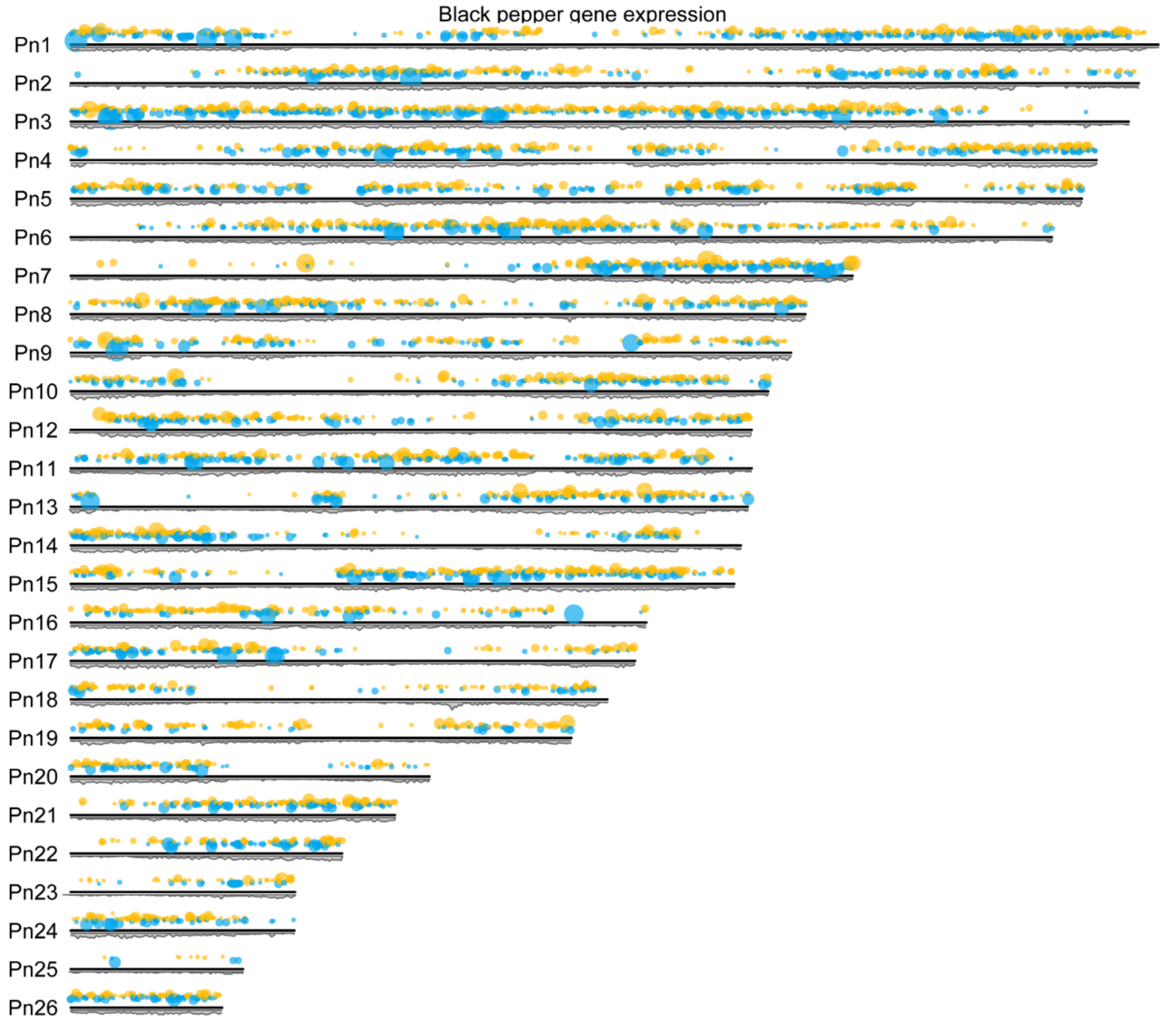

Supplementary Figure 38. The chromosome distribution of differentially expressed genes in berry and other tissues. The distribution of points is based on the log2 fold change and the size represents the p-value. The colours indicated up- (yellow) and under-expressed (cyan) expression genes.

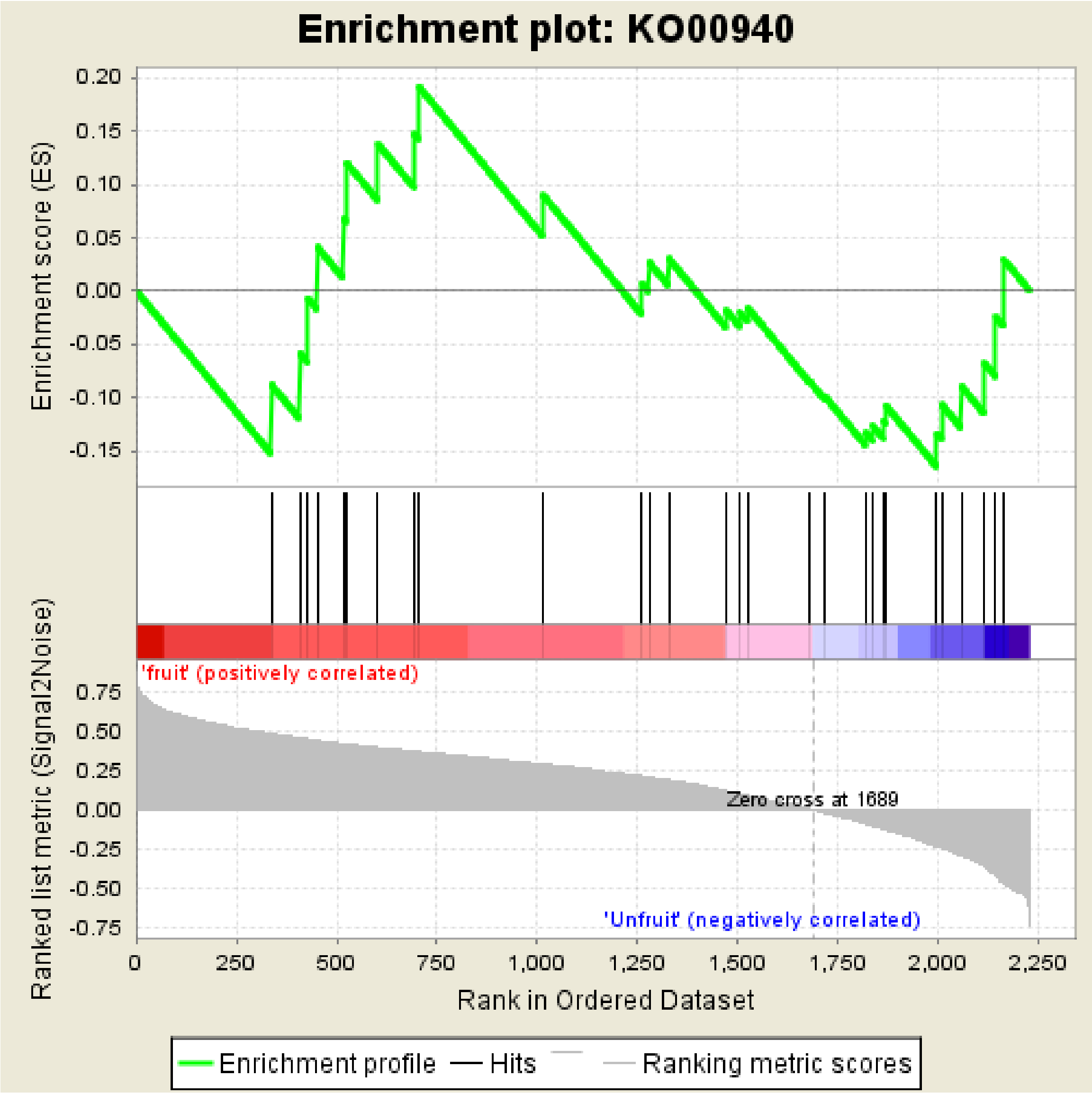

Supplementary Figure 39. Gene set enrichment analysis of the phenylpropanoid pathway in black pepper genome. Following the calculation of the enrichment score (ES), the enrichment plot illustrates specific gene sets associated with the differences between fruit and non-fruit tissues.

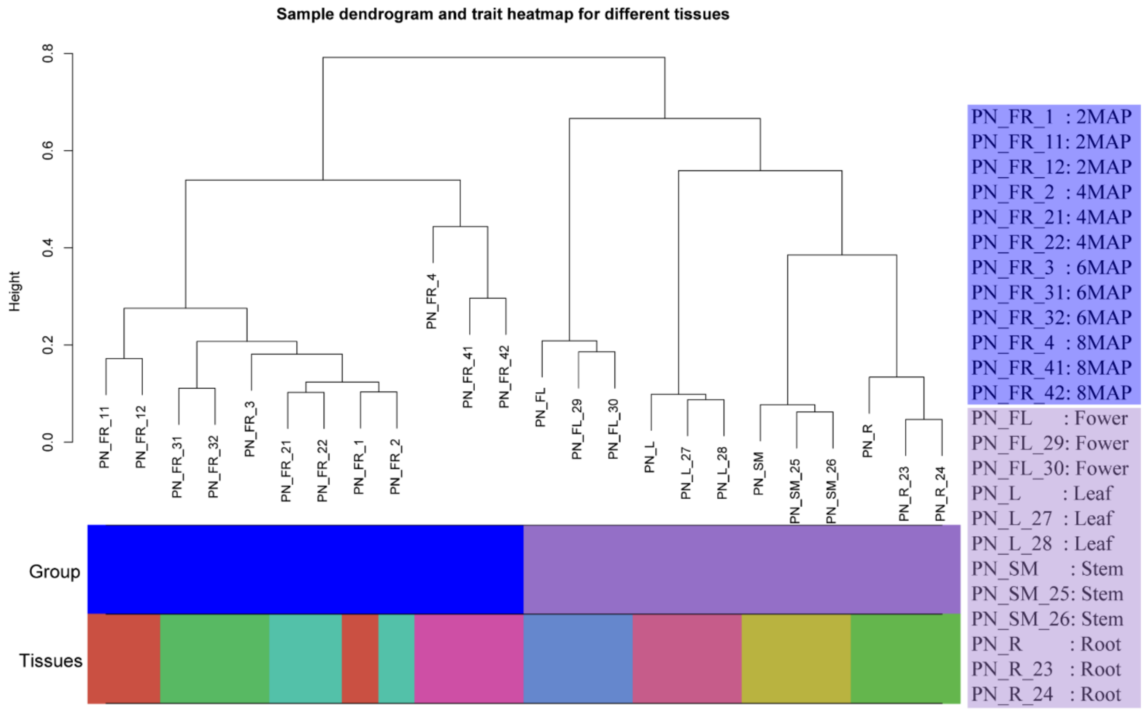

Supplementary Figure 40. Sample dendrogram and trait heatmap for different tissues from the WGCNA. Each colour in the dendrogram indicates one tissue.

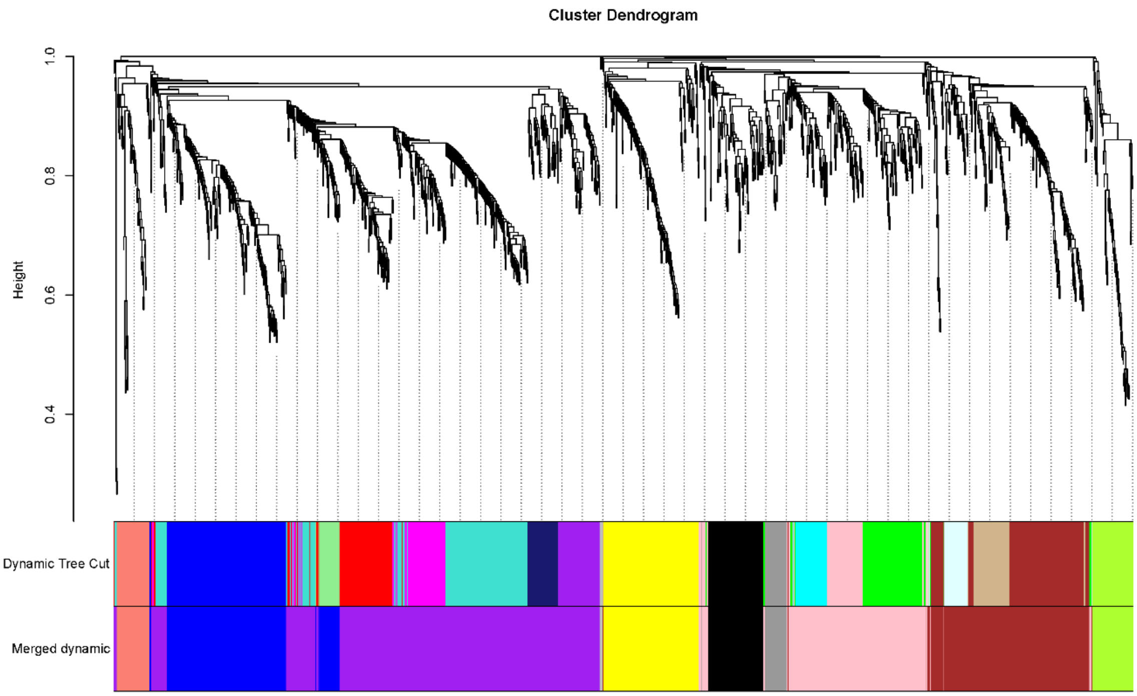

Supplementary Figure 41. Clustering dendrogram of genes together with assigned merged module colours and the original module colours. The different colours under the dendrogram show co-expressed modules identified using WGCNA.

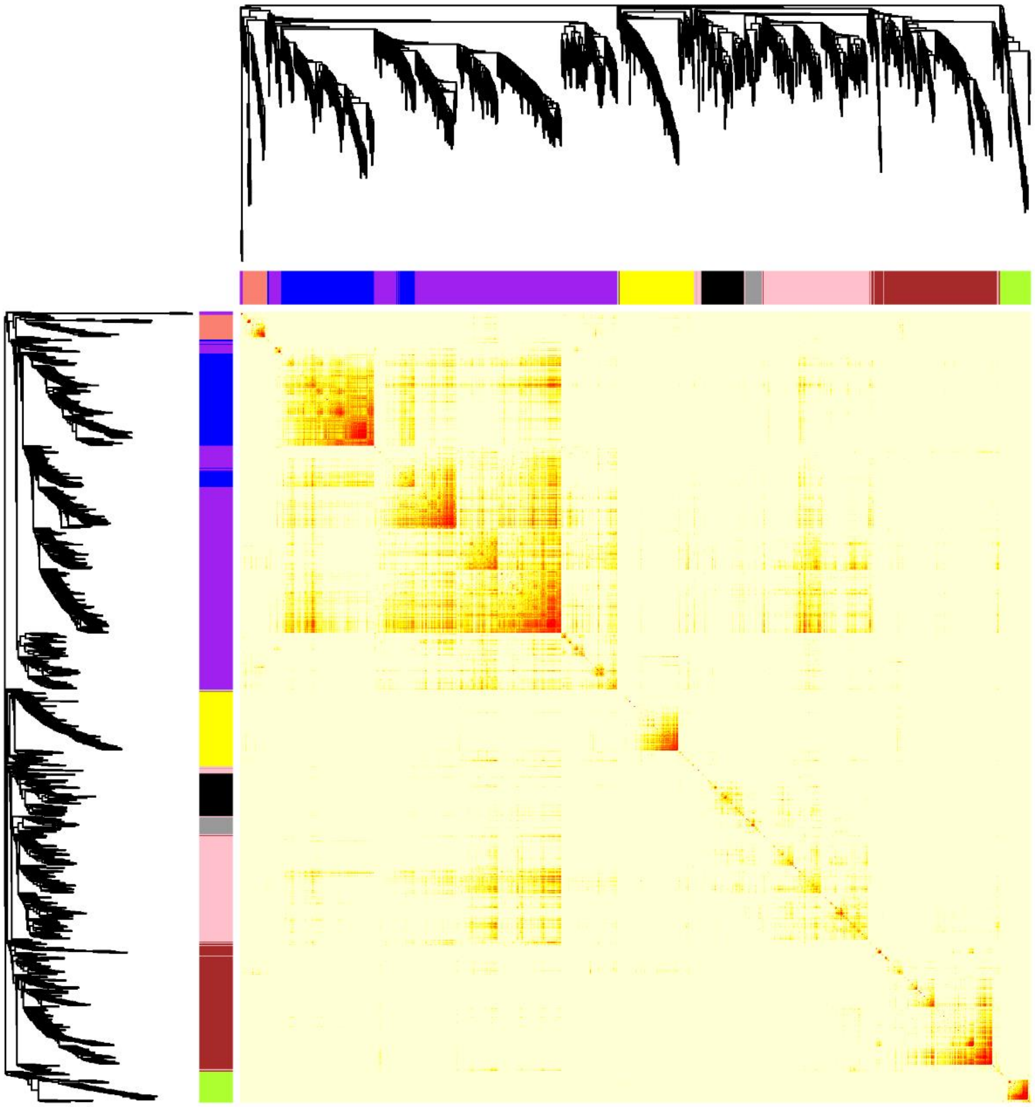

Supplementary Figure 42. Heatmap of the gene network using Topological Overlap Matrix among all genes identified from different tissues. The left side and the top represent the gene dendrogram and module. The colour bar that next to the dendrogram shows the co-expressed modules.

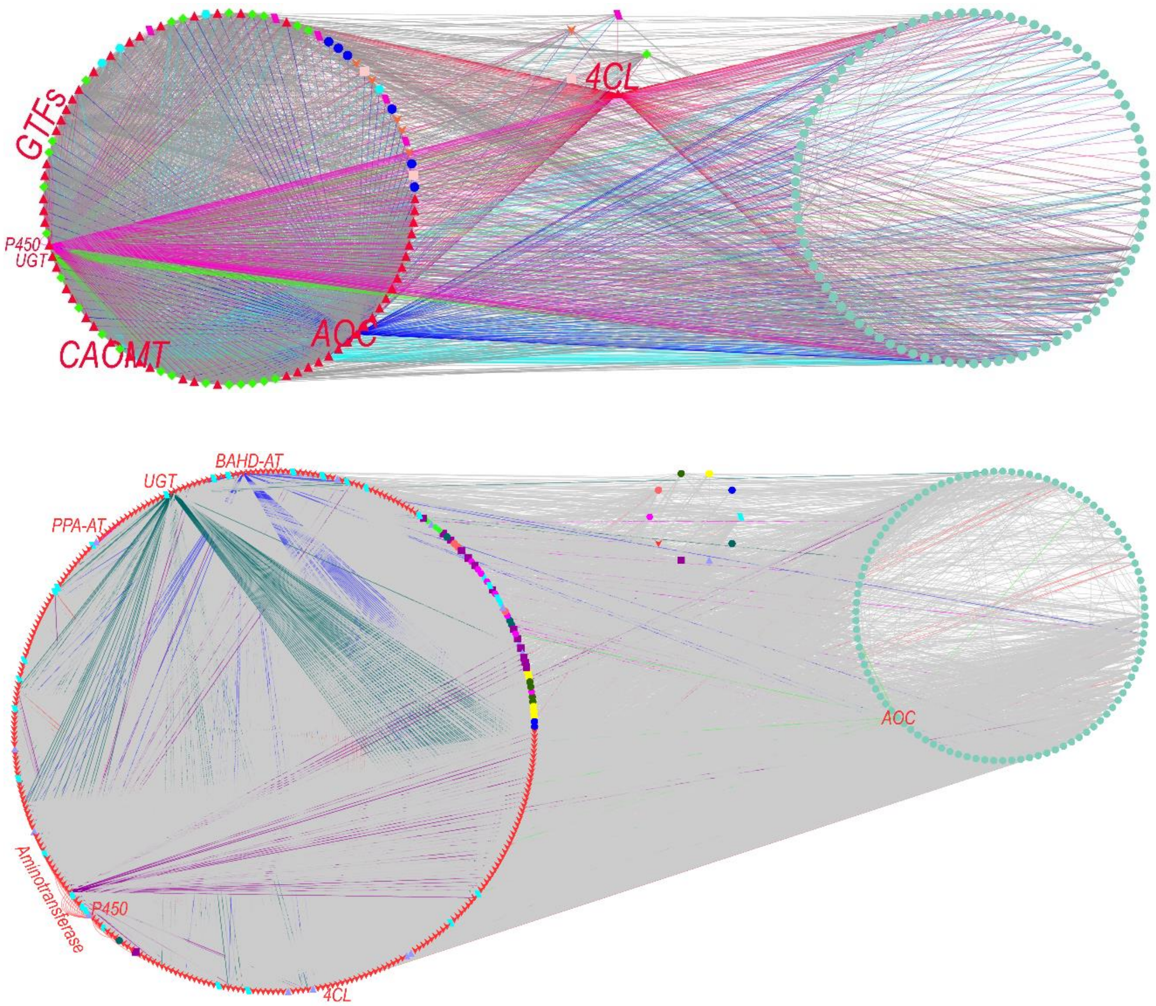

Supplementary Figure 43. **Co-regulatory network shows genes that participate in piperine biosynthesis in black pepper.** The nodes presented in different colour and shapes were used to distinguish clusters with highly interconnected regions in the network. Lines in different colours indicate the connections with specific genes.

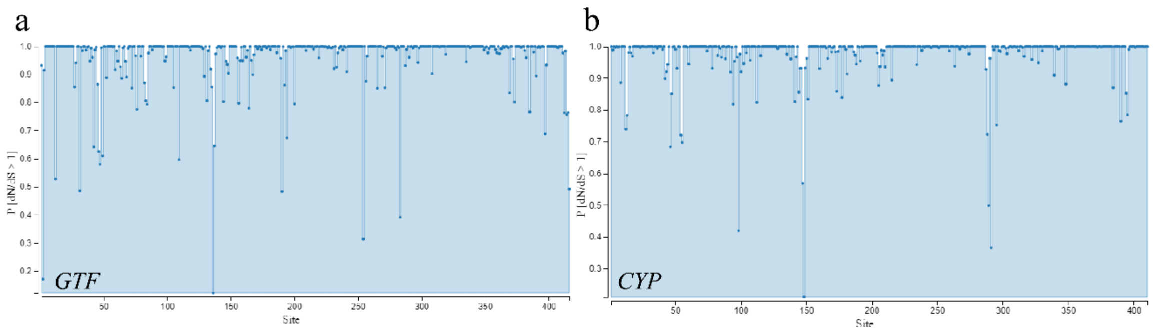

Supplementary Figure 44. SLAC site graph. The p-value of dN/dS >1 obtained using the SLAC method across the alignment of *GTF* (a) and *CYP* (b) sequences. Sites indicate statistically significant evidence for codons under selection when p[dN/dS >1] < 0.1.

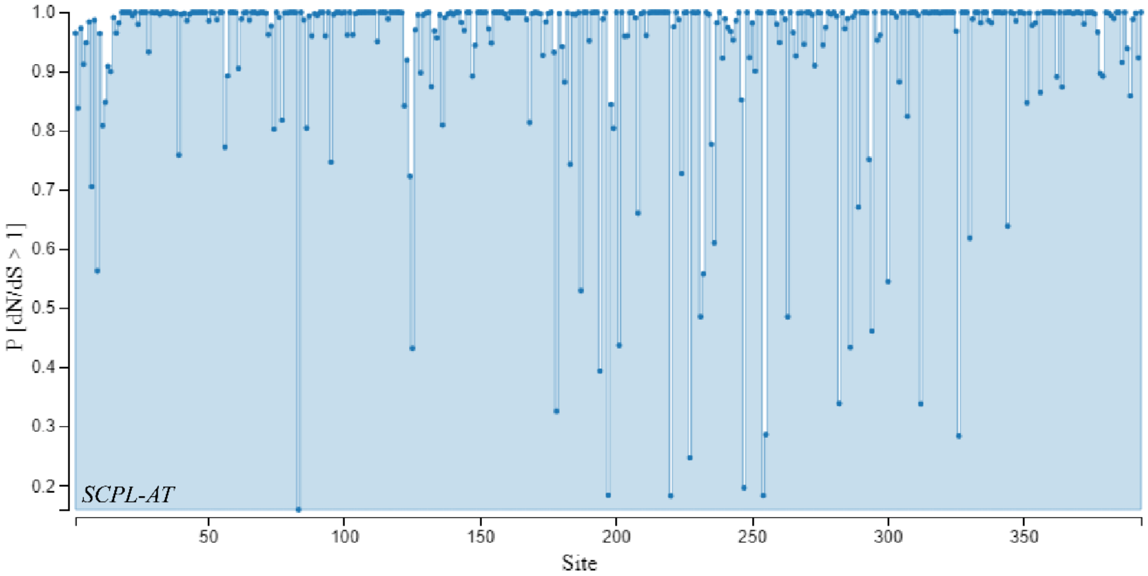

Supplementary Figure 45. SLAC site graph. The p-value of dN/dS >1 obtained using the SLAC (single-likelihood ancestor counting) method across the alignment of *SCPL-AT* sequences. Sites indicate statistically significant evidence for codons under selection when p[dN/dS >1] < 0.1.

Supplementary Table 1. Genome survey of black pepper.

K-mer	K-mer number	K-mer Depth	Genome Size (Mb)	Revised Genome Size (Mb)	Heterozygous Retio (%)	Repeat (%)
17	78,519,660,276	101	777.42	761.74	1.33	59.54

Supplementary Table 2. BioNano molecule quality report.

**Molecule Quality Report**	
Enzyme	DLE-1
Molecules Number	3,336,606
Total Length (Mb)	316,350.85
Quantity (Gb)	177.3
Avg. N50 (Kb) (>=150 Kb)	266
Avg. N50 (Kb) (>=20 Kb)	176
Avg. Label Density (per 100 Kb)	14.21
Avg. Map Rate (%)	50.60%
Estimated Effective Coverage	128X
Avg. False Positive	7.74%
Avg. False Negative	11.42%

Supplementary Table 3. Hi-C data quality report.

Truncating and Mapping		
	**Read 1**	**Read 2**
Total Reads	354,073,485	354,073,485
Not Truncated	186,364,059	190,021,708
Truncated	167,709,426	164,051,777
Too short to map	8,001,178	8,158,149
Average length of truncated sequence	76.99	76.51
Unique Alignments	261,779,237	259,465,003
Multiple Alignments	64,842,355	63,992,019
Failed To Align	19,450,715	22,458,314

Filtering	
	**Di-Tag Count**
Valid Pairs	166,420,467
Invalid Pairs	21,894,375
Same Circularised	1,215,285
Same Fragment Dangling Ends	154,806
Same Fragment Internal	1,834,879
Re-ligation	3,144,692
Contiguous Sequence	289,115
Total Pairs	188,314,842

De-duplication (Percentage uniques: 74.95)		
	**All Di-Tags**	**Unique Di-Tags**
Read Pairs	166,420,467	124,737,933
Cis-close (< 10Kb)	9,548,381	7,120,094
Cis-far (> 10Kb)	60,670,406	45,235,721
Trans	96,201,680	72,382,118

Supplementary Table 4. Statistics of completeness of the black pepper genome based on 248 CEGs.

**Complete Match**					
	**Prots**	**%Completeness**	**Total**	**Average**	**%Ortho**
Complete	234	94.35	640	2.74	78.63
Group 1	62	93.94	145	2.34	69.35
Group 2	51	91.07	120	2.35	64.71
Group 3	57	93.44	165	2.89	87.72
Group 4	64	98.46	210	3.28	90.62
**Partial Match**					
Total	244	98.39	738	3.02	85.25
Group 1	64	96.97	166	2.59	78.12
Group 2	54	96.43	147	2.72	75.93
Group 3	61	100.00	192	3.15	91.80
Group 4	65	100.00	233	3.58	93.85

These results are based on the set of genes selected by Genis Parra

Key:

Prots = number of 248 ultra-conserved CEGs present in genome

%Completeness = percentage of 248 ultra-conserved CEGs present

Total = total number of CEGs present including putative orthologs

Average = average number of orthologs per CEG

%Ortho = percentage of detected CEGS that have more than 1 ortholog

Listing missing proteins in each category

Category: Complete

KOG0018	KOG0062	KOG0209	KOG0346
KOG0376	KOG0434	KOG0741	KOG0969
KOG1272	KOG1795	KOG1889	KOG1936
KOG2036	KOG2311		

Category: Partial

KOG0062	KOG0346	KOG0376	KOG1889

Supplementary Table 5. Report of BUSCO results for the black pepper genome.

C:96.1%[S:77.0%,D:19.1%],F:1.2%,M:2.7%,n:430	
413	Complete BUSCOs (C)
331	Complete and single-copy BUSCOs (S)
82	Complete and duplicated BUSCOs (D)
5	Fragmented BUSCOs (F)
12	Missing BUSCOs (M)
430	Total BUSCO groups searched

Supplementary Table 6. Pfam protein domain models used in LTR retrotransposon/retrovirus-specific domains analysis.

Pfam accession#	Pfam ID	Description
PF00067	p450	Cytochrome P450
PF00069	Pkinase	Protein kinase domain
PF00075	RNase_H	RNase H
PF00076	RRM_1	RNA recognition motif
PF00098	zf-CCHC	Zinc knuckle
PF00153	Mito_carr	Mitochondrial carrier
PF00385	Chromo	Chromo (CHRromatin Organisation MOdifier) domain
PF00628	PHD	PHD-finger
PF01344	Kelch_1	Kelch motif
PF01348	Intron_maturas2	Type II intron maturase
PF01824	MatK_N	MatK/TrnK amino terminal region
PF02160	Peptidase_A3	Cauliflower mosaic virus peptidase (A3)
PF03078	ATHILA	ATHILA ORF-1 family
PF03107	C1_2	C1 domain
PF03357	Snf7	Snf7
PF03463	eRF1_1	eRF1 domain 1
PF03464	eRF1_2	eRF1 domain 2
PF03465	eRF1_3	eRF1 domain 3
PF03732	Retrotrans_gag	Retrotransposon gag protein
PF04094	DUF390	Protein of unknown function (DUF390)
PF04146	YTH	YTH protein domain
PF04195	Transposase_28	Putative gypsy type transposon
PF04578	DUF594	Protein of unknown function, DUF594
PF04852	DUF640	Protein of unknown function (DUF640)
PF04937	DUF659	Protein of unknown function (DUF 659)
PF05699	Dimer_Tnp_hAT	hAT family C-terminal dimerisation region
PF05970	PIF1	PIF1-like helicase

**Pfam accession#**	**Pfam ID**	**Description**
PF06886	TPX2	Targeting protein for Xklp2
PF07279	DUF1442	Protein of unknown function (DUF1442)
PF07727	RVT_2	Reverse transcriptase (RNA-dependent DNA polymerase)
PF08022	FAD_binding_8	FAD-binding domain
PF08284	RVP_2	Retroviral aspartyl protease
PF10551	MULE	MULE transposase domain
PF12776	Myb_DNA-bind_3	Myb/SANT-like DNA-binding domain
PF13359	DDE_Tnp_4	DDE superfamily endonuclease
PF13456	RVT_3	Reverse transcriptase-like
PF13837	Myb_DNA-bind_4	Myb/SANT-like DNA-binding domain
PF13912	zf-C2H2_6	C2H2-type zinc finger
PF13961	DUF4219	Domain of unknown function (DUF4219)
PF13966	zf-RVT	zinc-binding in reverse transcriptase
PF13968	DUF4220	Domain of unknown function (DUF4220)
PF13976	gag_pre-integrs	GAG-pre-integrase domain
PF14111	DUF4283	Domain of unknown function (DUF4283)
PF14223	Retrotran_gag_2	gag-polypeptide of LTR copia-type
PF14244	Retrotran_gag_3	gag-polypeptide of LTR copia-type
PF14372	DUF4413	Domain of unknown function (DUF4413)
PF14392	zf-CCHC_4	Zinc knuckle
PF14624	Vwaint	VWA / Hh protein intein-like
PF14683	CBM-like	Polysaccharide lyase family 4, domain III
PF16561	AMPK1_CBM	Glycogen recognition site of AMP-activated protein kinase
PF17123	zf-RING_11	RING-like zinc finger

Supplementary Table 7. Repeat sequences in the black pepper genome assembly.

		**number of elements***	**length of occupied**	**percentage of sequence**
SINEs:		104	16,555	0
LINEs:		17,482	12,505,878	1.64
	LINE1	9,168	7,756,560	1.02
	LINE2	63	111,025	0.01
	L3/CR1	0	0	0
	RTE	1,224	738,343	0.1
LTR elements:		32,3397	282,982,505	37.17
	Caulimovirus	2,832	3,081,483	0.4
	Copia	92,691	69,684,226	9.15
	Gypsy	201,723	193,127,498	25.37
	Pao	1,390	581,281	0.08
	DIRS	185	110,255	0.01
	Retro	4,001	2,284,508	0.3
	BEL	579	458,550	0.06
DNA elements:		165,462	78,653,714	10.33
	Academ	192	83,366	0.01
	Crypton	222	250,542	0.03
	Dada	333	146,366	0.02
	EnSpm/CACTA	10,804	4,699,167	0.62
	Ginger	546	179,169	0.02
	Harbinger	2,613	1,108,579	0.15
	hAT	15,231	7,703,783	1.01
	Helitron	4,460	1,835,765	0.24
	ISL2EU	133	31,261	0
	Kolobok	487	300,690	0.04
	Mariner	130	94,128	0.01
	MITEs	44,040	14,775,143	1.94
	MuDR	40,737	27,926,964	3.67
	Novosib	82	44,603	0.01
	P	19	8,005	0
	Polinton	3,249	1,634,254	0.21
	Sola	941	275,878	0.04
	Transib	96	32,166	0
Unclassified:		50,072	30,202,270	3.97
Total	interspersed	repeats:	404,360,922	53.12
Simple repeats:		214,761	10,432,062	1.37
Low complexity:		31,635	1,712,306	0.22

Supplementary Table 8. The percentage of transposable elements in study species.

Species	NonLTR(%)	LTR (%)	DNA (%)
Physcomitrella patens	0.27	98.62	1.11
Selaginella moellendorffii	12.08	64.76	23.16
Amborella trichopoda	11.76	67.42	20.81
Piper nigrum	3.37	75.08	21.54
Cinnamomum kanehirae	10.33	71.75	17.92
Liriodendron chinense	3.38	90.99	5.63
Oryza sativa	6.18	59.45	34.37
Dendrobium officinale	20.81	67.28	11.90
Ananas comosus	13.27	58.91	27.82
Arabidopsis thaliana	12.33	46.19	41.48
Camellia sinensis	4.84	84.55	10.61
Capsicum annuum	2.20	91.63	6.17
Citrus sinensis	13.40	65.64	20.95
Coffea canephora	8.49	73.80	17.71
Macleaya cordata	16.50	61.66	21.84
Nelumbo nucifera	14.00	83.87	2.13
Papaver somniferum	10.69	79.67	9.63
Vitis vinifera	13.50	71.12	15.38

Supplementary Table 9. The percentage of all type transposable elements in assembled genomes and ratio of Gypsy-to-Copia.

Species	LINE (%)	SINE (%)	NonLTR (%)	LTR/Gypsy (%)	LTR/Copia (%)	LTR (%)	MITEs (%)	Helitron (%)	DNA (%)	Gypsy/Copia
Physcomitrella patens	0.238882	0.00589554	0.269466	91.9287	6.59107	98.6216	0.564275	0.263793	1.10892	13.9475
Selaginella moellendorffii	10.8795	0.395128	12.0779	55.9411	3.61446	64.7625	1.28266	4.5696	23.1596	15.477
Amborella trichopoda	10.5821	1.10639	11.7641	48.1081	13.232	67.4246	0.0222665	0.157294	20.8113	3.63575
Cinnamomum kanehirae	8.32663	0.701993	10.328	39.2482	25.6013	71.7535	0.0075476	1.06792	17.9185	1.53306
Liriodendron chinense	2.41906	0.627161	3.37903	71.2597	19.4845	90.9897	0.200547	0.452443	5.63122	3.65725
Piper nigrum	3.35176	0.00480597	3.37357	51.161	18.4139	75.0817	4.00207	0.497767	21.5447	2.77839
Ananas comosus	10.265	2.07262	13.2687	36.772	14.0795	58.909	0.0104698	0.481715	27.8224	2.61174
Oryza sativa	5.97437	0.207163	6.18177	46.6105	10.5182	59.4471	13.009	3.44184	34.3712	4.43139
Dendrobium officinale	19.5517	0.545021	20.814	19.9458	45.2208	67.284	0.0111522	0.880335	11.902	0.441076
Arabidopsis thaliana	11.3343	0.815834	12.3345	33.0149	9.7523	46.1852	17.2949	5.37808	41.4802	3.38534
Camellia sinensis	3.96202	0.14366	4.84041	68.84	13.1092	84.5464	0.736759	0.275014	10.6132	5.25127
Capsicum annuum	2.1154	0.0424391	2.20363	81.5558	8.47787	91.6269	1.05834	0.11994	6.16945	9.61984
Citrus sinensis	11.3003	1.31054	13.4011	27.4413	27.337	65.6445	5.38481	0.738124	20.9544	1.00381
Coffea canephora	7.25349	0.168594	8.49411	57.3656	13.8002	73.7984	5.72844	1.55791	17.7075	4.15688
Macleaya cordata	11.9969	1.16828	16.4988	29.096	26.8851	61.6563	0.0511814	1.14564	21.8448	1.08223
Nelumbo nucifera	9.29088	4.70321	13.9987	35.8935	47.8121	83.8698	0.0249146	0.195305	2.13143	0.75072
Vitis vinifera	12.4714	0.267305	13.4965	37.2871	30.7614	71.1203	0.0138565	0.351128	15.3832	1.21214
Papaver somniferum	9.46844	0.0004104	10.6937	45.3976	32.6018	79.6733	0.0923317	0.323755	9.63301	1.39249

Supplementary Table 10. Statistics of synteny analysis for *Amborella trichopoda* and black pepper genome.

Genome and Annotation Statistics

**Species**	**Seqs**	**Total Kb**	**genes**	**genes (%)**	**Max Kb**	**Min Kb**	**< 100Kb**	**100Kb-1Mb**	**1Mb-10Mb**	**>10Mb**
Amborella trichopoda	245	675897	53796	44%	15980	101	0	86	0	4
Piper nigrum	26	760437	63427	23%	48451	14906	0	0	0	26

Anchor Statistics

**Species**	**Anchors**	**InBlocks**	**Annotated**	**Coverage**	**<100bp**	**100bp-1Kb**	**1Kb-10Kb**	**>10Kb**
Amborella trichopoda	22847	19%	78%	8%	598	11881	8276	2092
Piper nigrum	22847	19%	72%	5%	649	12760	9243	195

Block Statistics

**Species**	**Blocks**	**Coverage**	**DoubleCov**	**Inverted**	**GenesHit**	**< 100Kb**	**100Kb-1Mb**	**1Mb-10Mb**	**>10Mb**
Amborella trichopoda	316	40%	31%	139	9	0	34	282	0
Piper nigrum	316	34%	10%	139	8	17	197	101	1

Supplementary References

1 Marçais, G. & Kingsford, C. A fast, lock-free approach for efficient parallel counting of occurrences of k-mers. *Bioinformatics* **27**, 764 (2011).

2 Liu, B. *et al.* Estimation of genomic characteristics by analyzing k-mer frequency in de novo genome projects. *Quantitative Biology* **35**, 62-67 (2013).

3 Quinlan, A. R. & Hall, I. M. BEDTools: a flexible suite of utilities for comparing genomic features. *Bioinformatics* **26**, 841 (2010).

4 Quinlan, A. R. BEDTools: the Swiss‐army tool for genome feature analysis. *Current protocols in bioinformatics* **47**, 11.12. 11-11.12. 34 (2014).

5 Li, H. Aligning sequence reads, clone sequences and assembly contigs with BWA-MEM. **1303** (2013).

6 Li, H. *et al.* The Sequence Alignment/Map format and SAMtools. *Bioinformatics* **25**, 2078-2079 (2009).

7 McKenna, A. *et al.* The Genome Analysis Toolkit: a MapReduce framework for analyzing next-generation DNA sequencing data. *Genome research* **20**, 1297-1303 (2010).

8 Gogarten, S. M. *et al.* GWASTools: an R/Bioconductor package for quality control and analysis of genome-wide association studies. *Bioinformatics* **28**, 3329-3331 (2012).

9 Finn, R. D. *et al.* The Pfam protein families database: towards a more sustainable future. *Nucleic Acids Research* **44**, D279-D285 (2016).

10 Lowe, T. M. & Chan, P. P. tRNAscan-SE On-line: integrating search and context for analysis of transfer RNA genes. *Nucleic Acids Research* **44**, W54-W57 (2016).

11 Lagesen, K. *et al.* RNAmmer: consistent and rapid annotation of ribosomal RNA genes. *Nucleic Acids Research* **35**, 3100-3108 (2007).

12 Li, A., Zhang, J. & Zhou, Z. PLEK: a tool for predicting long non-coding RNAs and messenger RNAs based on an improved k-mer scheme. *BMC Bioinformatics* **15**, 311 (2014).

13 Singh, U., Khemka, N., Rajkumar, M. S., Garg, R. & Jain, M. PLncPRO for prediction of long non-coding RNAs (lncRNAs) in plants and its application for discovery of abiotic stress-responsive lncRNAs in rice and chickpea. *Nucleic acids research* **45**, e183-e183 (2017).

14 Nawrocki, E. P. *et al.* Rfam 12.0: updates to the RNA families database. *Nucleic Acids Research* **43**, D130 (2015).

15 Nawrocki, E. P. & Eddy, S. R. Infernal 1.1: 100-fold faster RNA homology searches. *Bioinformatics* **29**, 2933-2935, doi:10.1093/bioinformatics/btt509 (2013).

16 Lyons, E. & Freeling, M. How to usefully compare homologous plant genes and chromosomes as DNA sequences. *The Plant Journal* **53**, 661-673 (2010).

17 Wang, D., Zhang, Y., Zhang, Z., Zhu, J. & Yu, J. KaKs_Calculator 2.0: A Toolkit Incorporating Gamma-Series Methods and Sliding Window Strategies. *Genomics Proteomics Bioinformatics* **8**, 77-80 (2010).

18 Wang, Y. *et al.* MCScanX: a toolkit for detection and evolutionary analysis of gene synteny and collinearity. *Nucleic acids research* **40**, e49-e49 (2012).

19 Guo, L. *et al.* The opium poppy genome and morphinan production. *Science* **362**, 343-347 (2018).
20 Wood, A. B., Barrow, M. L. & James, D. J. Piperine determination in pepper (Piper nigrum L.) and its oleoresins-a reversed-phase high-performance liquid chromatographic method. *Flavour & Fragrance Journal* **3**, 55-64 (2010).
21 Andrews, S. FastQC: a quality control tool for high throughput sequence data. (2010).
22 Ewels, P., Magnusson, M., Lundin, S. & Käller, M. MultiQC: summarize analysis results for multiple tools and samples in a single report. *Bioinformatics* **32**, 3047-3048 (2016).
23 Kolde, R. & Kolde, M. R. Package 'pheatmap'. (2018).
24 Krzywinski, M. *et al.* Circos: an information aesthetic for comparative genomics. *Genome research* **19**, 1639-1645 (2009).
25 Gel, B. & Serra, E. karyoploteR: an R/Bioconductor package to plot customizable genomes displaying arbitrary data. *Bioinformatics* **33**, 3088-3090 (2017).
26 Love, M. I., Huber, W. & Anders, S. Moderated estimation of fold change and dispersion for RNA-seq data with DESeq2. *Genome Biology* **15**, 550 (2014).
27 Ignatiadis, N., Klaus, B., Zaugg, J. B. & Huber, W. Data-driven hypothesis weighting increases detection power in genome-scale multiple testing. *Nature methods* **13**, 577 (2016).
28 Shannon, P. *et al.* Cytoscape: a software environment for integrated models of biomolecular interaction networks. *Genome research* **13**, 2498-2504 (2003).
29 Bader, G. D. & Hogue, C. W. V. An automated method for finding molecular complexes in large protein interaction networks. *BMC bioinformatics* **4**, 2-2, doi:10.1186/1471-2105-4-2 (2003).
30 Denoeud, F. *et al.* The coffee genome provides insight into the convergent evolution of caffeine biosynthesis. *Science* **345**, 1181-1184 (2014).
31 Kim, S. *et al.* Genome sequence of the hot pepper provides insights into the evolution of pungency in Capsicum species. *Nature Genetics* **46**, 270-278 (2014).
32 Wei, C. *et al.* Draft genome sequence of *Camellia sinensis* var. sinensis provides insights into the evolution of the tea genome and tea quality. *Proc Natl Acad Sci U S A* **115**, 201719622 (2018).
33 Jaillon, O. *et al.* The grapevine genome sequence suggests ancestral hexaploidization in major angiosperm phyla. *Nature* **449**, 463 (2007).
34 Xu, Q. *et al.* The draft genome of sweet orange (*Citrus sinensis*). *Nature Genetics* **45**, 59-66, doi:10.1038/ng.2472 (2012).
35 Gui, S. *et al.* Improving *Nelumbo nucifera* genome assemblies using high-resolution genetic maps and BioNano genome mapping reveals ancient chromosome rearrangements. *Plant Journal for Cell & Molecular Biology* (2018).
36 Liu, X. *et al.* The genome of medicinal plant *Macleaya cordata* provides new insights into benzylisoquinoline alkaloids metabolism. *Molecular plant* **10**, 975-989 (2017).
37 The Arabidopsis Genome Initiative. Analysis of the genome sequence of the flowering plant Arabidopsis thaliana. *Nature* **408**, 796-815, doi:10.1038/35048692 (2000).

38 Goff, S. A. *et al.* A draft sequence of the rice genome (*Oryza sativa* L. ssp. japonica). *Science* **296**, 92-100 (2002).
39 Ming, R. *et al.* The pineapple genome and the evolution of CAM photosynthesis. *Nature Genetics* **47**, 1435-1442 (2015).
40 Liang, Y. *et al.* The genome of *Dendrobium officinale* illuminates the biology of the important traditional Chinese orchid herb. *Molecular Plant* **8**, 922-934 (2015).
41 Chen, J. *et al.* Liriodendron genome sheds light on angiosperm phylogeny and species–pair differentiation. *Nature Plants* **5**, 18-25, doi:10.1038/s41477-018-0323-6 (2019).
42 Chaw, S.-M. *et al.* Stout camphor tree genome fills gaps in understanding of flowering plant genome evolution. *Nature Plants* **5**, 63-73, doi:10.1038/s41477-018-0337-0 (2019).
43 Ibarra-Laclette, E. *et al.* Deep sequencing of the Mexican avocado transcriptome, an ancient angiosperm with a high content of fatty acids. *BMC Genomics* **16**, 599, doi:10.1186/s12864-015-1775-y (2015).
44 Albert, V. A. *et al.* The Amborella genome and the evolution of flowering plants. *Science* **342**, 1241089 (2013).
45 Wan, T. *et al.* A genome for gnetophytes and early evolution of seed plants. *Nature Plants* **4**, 82-89, doi:10.1038/s41477-017-0097-2 (2018).
46 Nystedt, B. *et al.* The Norway spruce genome sequence and conifer genome evolution. *Nature* **497**, 579-584, doi:10.1038/nature12211 (2013).
47 Banks, J. A. *et al.* The Selaginella genome identifies genetic changes associated with the evolution of vascular plants. *Science* **332**, 960-963, doi:10.1126/science.1203810 (2011).
48 Rensing, S. A. *et al.* The Physcomitrella genome reveals evolutionary insights into the conquest of land by plants. *Science* **319**, 64-69 (2008).
49 Li, L., Jr, S. C. & Roos, D. S. OrthoMCL: identification of ortholog groups for eukaryotic genomes. *Genome Research* **13**, 2178-2189 (2003).
50 De Bie, T., Cristianini, N., Demuth, J. P. & Hahn, M. W. CAFE: A computational tool for the study of gene family evolution. *Bioinformatics* **22**, 1269-1271 (2006).
51 Cummings, M. P. *HyPhy (Hypothesis Testing Using Phylogenies)*. (John Wiley & Sons, Ltd, 2014).
52 Delport, W., Poon, A. F., Frost, S. D. & Kosakovsky Pond, S. L. Datamonkey 2010: a suite of phylogenetic analysis tools for evolutionary biology. *Bioinformatics* **26**, 2455-2457 (2010).
53 Kosakovsky Pond, S. L., Posada, D., Gravenor, M. B., Woelk, C. H. & Frost, S. D. GARD: a genetic algorithm for recombination detection. *Bioinformatics* **22**, 3096 (2006).
54 Kosakovsky Pond, S. L. & Frost, S. D. Not so different after all: a comparison of methods for detecting amino acid sites under selection. *Molecular Biology & Evolution* **22**, 1208 (2005).
55 Murrell, B. *et al.* Detecting individual sites subject to episodic diversifying selection. *PLoS genetics* **8**, e1002764 (2012).
