# Supplementary material for: The chromosome-scale reference genome of black pepper provides insight into piperine biosynthesis
Source: Nat Commun. 2019 Oct 16;10:4702. doi: 10.1038/s41467-019-12607-6 (PMC6795880; doi:10.1038/s41467-019-12607-6)
Supplement: Supplementary file 3 — Reporting Summary [file 41467_2019_12607_MOESM3_ESM.pdf]

nature research

Corresponding author(s): Shuangxia Jin

Last updated by author(s): Sep 9, 2019

Reporting Summary

Nature Research wishes to improve the reproducibility of the work that we publish. This form provides structure for consistency and transparency in reporting. For further information on Nature Research policies, see Authors & Referees and the Editorial Policy Checklist .

Statistics

For all statistical analyses, confirm that the following items are present in the figure legend, table legend, main text, or Methods section.

n/a	Confirmed	
☐	☒	The exact sample size (n) for each experimental group/condition, given as a discrete number and unit of measurement
☒	☐	A statement on whether measurements were taken from distinct samples or whether the same sample was measured repeatedly
☒	☐	The statistical test(s) used AND whether they are one- or two-sided<br>*Only common tests should be described solely by name; describe more complex techniques in the Methods section.*
☒	☐	A description of all covariates tested
☒	☐	A description of any assumptions or corrections, such as tests of normality and adjustment for multiple comparisons
☒	☐	A full description of the statistical parameters including central tendency (e.g. means) or other basic estimates (e.g. regression coefficient) AND variation (e.g. standard deviation) or associated estimates of uncertainty (e.g. confidence intervals)
☒	☐	For null hypothesis testing, the test statistic (e.g. F, t, r) with confidence intervals, effect sizes, degrees of freedom and P value noted<br>*Give P values as exact values whenever suitable.*
☐	☒	For Bayesian analysis, information on the choice of priors and Markov chain Monte Carlo settings
☒	☐	For hierarchical and complex designs, identification of the appropriate level for tests and full reporting of outcomes
☒	☐	Estimates of effect sizes (e.g. Cohen's d, Pearson's r), indicating how they were calculated

Our web collection on statistics for biologists contains articles on many of the points above.

Software and code

Policy information about availability of computer code

Data collection	No software was used during data collection.
Data analysis	Falcon (v1.8.7), Blasr (v5.1), Arrow (v2.1.0), fragScaff (140324.1), Pilon (v1.22), bowtie2 (v2.3.4), PBSuite(15.8.24), LACHESIS (https://github.com/shendurelab/LACHESIS), Juicebox (v1.8.8), PacBio Iso-Seq pipeline (https://github.com/PacificBiosciences/IsoSeq3), Bionano Solve (https://bionanogenomics.com/wp-content/uploads/2017/10/30182-Bionano-Tools-Installation-Guide.pdf), BUSCO (v3), Augustus (v3.3.2), TransposonPSI (http://transposonpsi.sourceforge.net/), tRNAscan-SE (v2.0), BRAKER (https://github.com/Gaius-Augustus/BRAKER), GenomeTools (v1.5.10), EVidenceModeler (v1.1.1), InterProScan (v5.31-70.0), CAFE (v4.2), KaKs_Calculator (v2.0), MCScanx (http://chibba.pgml.uga.edu/mcscan2/), orthomclSoftware(v2.0.9), RAxML (v8.2.12), BEAST(v2.5.2), HISAT2 (v2.1.0), StringTie (v1.3.5), circos (v0.69-4).

For manuscripts utilizing custom algorithms or software that are central to the research but not yet described in published literature, software must be made available to editors/reviewers. We strongly encourage code deposition in a community repository (e.g. GitHub). See the Nature Research guidelines for submitting code & software for further information.

Data

Policy information about availability of data

All manuscripts must include a data availability statement. This statement should provide the following information, where applicable:

- Accession codes, unique identifiers, or web links for publicly available datasets
- A list of figures that have associated raw data
- A description of any restrictions on data availability

All the raw sequencing data generated during the current study have been deposited at NCBI as a BioProject under accession PRJNA529758. Transcriptome sequence reads have been deposited in the SRA database under BioProject number PRJNA529760. The genome assemblies and annotation files are available at the website http://cotton.hzau.edu.cn/EN/download.php. All the datasets generated and analyzed during the current study are available upon reasonable request. The source data underlying Figs. 1, 2a, 2b, 3 and 4a-h are provided as a Source Data file. A reporting summary for this Article is available as a Supplementary Information file.

Field-specific reporting

Please select the one below that is the best fit for your research. If you are not sure, read the appropriate sections before making your selection.

[x] Life sciences [ ] Behavioural & social sciences [ ] Ecological, evolutionary & environmental sciences

For a reference copy of the document with all sections, see nature.com/documents/nr-reporting-summary-flat.pdf

Life sciences study design

All studies must disclose on these points even when the disclosure is negative.

Sample size	For RNA-Seq and piperine determination experiments of 8 different tissues (root, stem, leaf, flower and berry at four different stages: 2 month after pollination (MAP), 4 MAP, 6 MAP, 8 MAP) of black pepper and used 3 biological replicates.
Data exclusions	All sequences data in this study, we only excluded sequences that were of low quality. For genome assembly, we only excluded the redundant sequences that were caused because of high heterozygosity.
Replication	There is various approaches were used to evaluate the completeness of assemblies genome. All replicates reported in the manuscript are biological replicates.
Randomization	No randomization was needed in this study.
Blinding	No blinding was done.

Reporting for specific materials, systems and methods

We require information from authors about some types of materials, experimental systems and methods used in many studies. Here, indicate whether each material, system or method listed is relevant to your study. If you are not sure if a list item applies to your research, read the appropriate section before selecting a response.

Materials & experimental systems

n/a	Involved in the study	
[x]	[ ]	Antibodies
[x]	[ ]	Eukaryotic cell lines
[x]	[ ]	Palaeontology
[x]	[ ]	Animals and other organisms
[x]	[ ]	Human research participants
[x]	[ ]	Clinical data

Methods

n/a	Involved in the study	
[x]	[ ]	ChIP-seq
[x]	[ ]	Flow cytometry
[x]	[ ]	MRI-based neuroimaging
