# Supplementary material for: The chromosome-scale reference genome of black pepper provides insight into piperine biosynthesis
Source: Nat Commun. 2019 Oct 16;10:4702. doi: 10.1038/s41467-019-12607-6 (PMC6795880; doi:10.1038/s41467-019-12607-6)
Supplement: Supplementary file 2 — Peer Review [file 41467_2019_12607_MOESM2_ESM.pdf]

Reviewers' comments:

Reviewer #1 (Remarks to the Author):

There are a number of issues with the phylogenomic aspects of the paper

There is no "dicot" clade. The term is used throughout the ms. That term is no longer used and has not been used for many years. Dicots were shown over three decades ago to be paraphyletic. There is a eudicot clade (the authors do use the term in a few places). The relationship of that eudicot clade to monocots and magnoliids is the proper phrasing.

The authors need to realize the limitations of their current phylogenomic hypothesis. Yes it is based on a lot of sequence data (although I was not able to find how many genes were actually used) but very few taxa. As a result, readers should place little confidence in the topology. The authors should read the recent summary of Soltis and Soltis in Nature Plants (2019; volume 5: 6-7). As they note, critical lineages are not included in the paper they have submitted-Chloranthales, Ceratophyllales. Another major problem in discussing relationships of magnoliids is that one clade of magnoliids still has not been sampled - Canellales. Thus, to make any statement about relationships with genome scale data remains incomplete and inconclusive.

Several studies have already employed numerous nuclear and plastid genes and numerous taxa in much better phylogenetic studies of angiosperms. Some have also shown that magnoliids are well supported and sister to monocots + eudicots. Thus, the results here support a lot of previous literature and that should be noted. But again, no hard conclusions can be made here with so few taxa. Again, see the Soltis and Soltis review for discussion of these concerns.

There are other problems with the phylogenomic portion of the ms. The authors use the term "paleoherb"; this is an old term for these plants that has inappropriate connotations and is not really used now. I would simply note that this is the first time a genome from a member of Piperales has been included.

Piper nigrum did not diverge from Amborella trichopoda at 226 mya. The ancestors may have diverged at that time. Not the living species -see below.

The divergence time estimate portion of the study is inadequate. It is very important to give a range of values around these divergence times-not a single absolute value. These are rough estimates and must be presented as such (see below)

Given the concerns raised in Soltis and Soltis 2019 (and the papers cited there in) it is hard to justify the publication of the phylogenomic part of this study. As noted the sampling is so poor that the results cannot be given serious credence. A new phylogenomic tree is not justified every time a new genome is published

Probably the main phylogenomic point to make if made at all is that with limited taxon sampling this is another nuclear genome level study to show the same tree revealed by plastid and some nuclear data. But then stress more crucial species must be sampled- the point made by Soltis and Soltis. This would require one or two sentences of text and then everything is placed in a supplement. The dating should either removed or done properly to provide error bars around estimates. The other option is to remove all mention of phylogeny-the authors really don't have the data to do those analyses correctly---many more taxa are needed.

Many parts of the paper are poorly written. The paper requires careful editing

Could the authors do more with their genome? What about synteny between the two subgenomes?

How much rearrangement has occurred post polyploidy? And could synteny to other angiosperm genomes, particularly Amborella be discussed? Is there conservation of gene order at that scale?

Other comments below

32. Piper was also considered as a model genus for studies of evolution because of strikingly diverse lineages amongst basal angiosperms Poorly worded-what is this meant to say?
47-48. which showing an evolution association and molecular basis of species-specific piperine biosynthesis from three major metabolic pathway

85-86. A very important point is not just that Piper is a magnoliid but first representative of Piperales. There are four sub clades recognized as orders within the magnoliids

206. These plants are not dicots. That group no longer is recognized or discussed. The authors must remove that term from the text throughout and also get caught up on the angiosperm phylogenetic literature-simple summaries as in the Judd et al. and Soltis et al. 2018 text books

212-216. Very poorly worded text

219 Piper nigrum did not diverge from Amborella trichopoda at 226 mya. Their ancestors may have diverged at that time. Not the living species

219-222 The remaining text discussing divergences is similarly presented incorrectly for the same reason
Also it is also important to give a range of values around these divergence times. They are rough estimates and must be presented as such

552 The formed time of black pepper allotetraploid poorly written

559. For assess the evolution Poor wording

560-565 How many genes were used to build the trees?

599 positive selection and episodic selection of gene poor wording
574 which obtained from Timetree poorly worded

Figures are not numbered

Phylogeny in fig 2 c. Ranges of values for divergence times must be given. Not absolute values-see comments above

Phylogeny in Fig 3-how was this rooted?

Reviewer #2 (Remarks to the Author):

Hu et al. described the high quality reference genome sequence of black pepper. By adopting multiple technology including long-read sequencing, optical mapping and chromatin interaction mapping, the authors assembled 26 pseudochromosomes with N50 scaffold length of 30 Mb. Furthermore, they cleared the taxonomic position of Piperales by comparative genomics and the molecular basis of piperine biosynthesis by comprehensive transcriptomic analyses. Overall the manuscript has fair enough merit to be published as a first high quality reference genome paper of a plant belong to

Piperales.

However, for improvement the quality of the manuscript, following concerns should be considered.

i) Highlighting gene family evolution across plant kingdom. The authors briefly mentioned gene copy-number evolution among plants including lower, basal, gymnosperm, and angiosperm plants. Because there are only a few of sequenced basal plants, we could poorly understand global gene evolution of the plants. If the authors could highlight features and differences for copy-number and evolution of important gene families (eg. disease-resistance genes and TFs) among the basal, gymnosperm and angiosperm plants given gene duplication history and selection pressures, it will be valuable biological resources and knowledge for the readers.

ii) Transposable elements (TEs) are one major evolutionary forces especially in plants. Like the gene family evolution study, authors need to present insights of TE evolution to help understanding of genome evolution in plant kingdom for readers. Compared to gymnosperms, angiosperms (dicot and monocot) and low plants, what are specific differences in TE repertories, expansion and insertion pattern?. LTR-retrotransposons and DNA-transposons are major TEs in plants and they could be classified as multiple subgroups. For example, LTR-gypsy family could be divided as groups like del, athila and the others considering their pol proteins. The repertories of subgroups are extremely diversified among plants. What are major elements and differences of TEs among the plants?. To address this issue, the author should perform exquisite annotation and comparison of TEs.

iii) The authors focused on their biological study for piperine biosynthesis which is the unique feature of Piper nigrum but I think that this story is too long and verbose.

Minors
Line 47: “evolution association” should be “evolutionary association”
Line 93: “assembled” should be “assembly”

Reviewer #3 (Remarks to the Author):

Hu et al present a chromosome scale genome assembly of the Magnoliid black pepper. Black pepper is presumably an allotetraploid and much of the genome is retained in duplicate. Using comparative genomics and expression analysis, the authors identified gene family expansions that may be related to piperine biosynthesis. Although the authors present a nice resource for the community, I have major and fundamental concerns that should be addressed before this manuscript is suitable for publication.

Major concerns:
1. Line 160. The total number of genes (24,814) and lack of duplicated single copy genes (based on BUSCO) would strongly suggest black pepper is diploid. Black pepper has 50 or 65% fewer genes than quinoa or cotton so this total gene number is not in line with other tetraploids.
Lines 191-194 state that 33,206 genes (or 52.16%) are retained as duplicates, bringing the total gene model number to 63,661 (I think?) and not 24,814 as stated in the text and in Table 1. I’m not sure which of these numbers is correct, but this is important for downstream analyses.

2. More details should be provided on the genome assembly, including the statistics for each assembly step. The authors estimated the heterozygosity of the genome was around 1.3% which should have resulted in assembly of two haplotypes by Falcon-Unzip. The total size of the bionano genome map was 1.3 Gb, or roughly 2x the haploid genome size (suggesting 2 haplotypes were assembled). It is unclear how this was integrated with the genome assembly, or how haplotypes were removed/filtered.

3. The HiC based heatmaps in Supplemental Figures 7 and 8 highlight major assembly issues in this genome. This most obvious errors can be seen in supplemental Figure 8e, but mistakes in scaffold order and orientation can be found in each chromosome. These issues are based on a lack of continuous interactions between neighboring contigs/scaffolds, but strong interactions elsewhere in the chromosome. Scaffolds in Figure 8u and 8z are pretty good (with some very small orientation issues) for comparison. The authors claim these regions are classified TADs and loops, but this is not true. These issues are manifested in Figure 1 where syntenic blocks between homeologous chromosomes are highly fragmented, and gene and LTR density are erratic instead of decreasing and increasing respectively near the centromeres. Because of these clear issues, it is difficult to assess the comparative genomics aspects of this manuscript. I would suggest the authors redo the HiC analysis using a different pipeline and manually inspect the final interaction matrix in a program like JuiceBox (https://github.com/aidenlab/Juicebox/wiki/Juicebox-Assembly-Tools) to fix these errors. This will substantially improve the assembly quality and utility of this genome for the community.

4. The venn diagram of shared and unique gene families in Figure 2a contains an unusual assortment of unrelated specie making it hard to interpret. It would be better to include the basal angiosperm Amborella and the two other magnoolids (Cinnamomum kanehirae and Liriodendron tulipifera) at a minimum.

5. The phylogeny in Figure 3 is wrong, making it difficult to interpret gene family gain/loss between lineages. For instance, Arabidopsis and Carica are both Brassicales (and should be sister here) whereas Malus is a Rosales and Citrus is in the order Sapindales. These fundamental issues make it difficult to interpret these results.

6. The two other Magnoolid genomes should be included in Figure 4a for gene family enrichment analysis. Figure 4a and b are difficult to interpret and it is hard to see if the expanded gene families have higher expression (indicating they are involved in unique processes in black pepper). I would suggest the authors use different colors and a scale in Figure 4b. The CYP90 clan is expanded in black pepper, but the expression of only one gene is shown in Figure 4b, again making it difficult to judge how meaningful these results are. It would be interesting to see if homologous genes are contributing to piperine biosynthesis differently.

We sincerely appreciate the editors and reviewers for their thoughtful comments and recommendations on our manuscript. Those comments are very helpful for revising and improving our paper, as well as the important guidance to other research. We have studied the comments carefully and have modified the manuscript accordingly. We hope this revised manuscript will meet the journal’s high standards. The main corrections are included in the revised manuscript and the response to the reviewers’ comments are as follows:

Reviewers' comments and our response:

Reviewer #1 (Remarks to the Author):

Question:

There are a number of issues with the phylogenomic aspects of the paper. There is no "dicot" clade. The term is used throughout the ms. That term is no longer used and has not been used for many years. Dicots were shown over three decades ago to be paraphyletic. There is a eudicot clade (the authors do use the term in a few places). The relationship of that eudicot clade to monocots and magnoliids is the proper phrasing.

Response: Thanks for your valuable comments of this manuscript. According to your suggestion, we have changed "dicot" to "eudicot " throughout the manuscript.

Question: The authors need to realize the limitations of their current phylogenomic hypothesis. Yes it is based on a lot of sequence data (although I was not able to find how many genes were actually used) but very few taxa. As a result, readers should place little confidence in the topology. The authors should read the recent summary of Soltis and Soltis in Nature Plants (2019; volume 5: 6-7). As they note, critical lineages are not included in the paper they have submitted-Chloranthales, Ceratophyllales. Another major problem in discussing relationships of magnoliids is that one clade of magnoliids still has not been sampled - Canellales. Thus, to make any statement about relationships with genome scale data remains incomplete and inconclusive.

Response: Thanks. These are helpful comments, and accordingly, we have rephrased all phylogenetic discussion and conclusions and have included the more recent relevant

literature on this topic (including Soltis and Soltis in Nature Plants, 2019). We realize the importance that included Canellales, Chloranthales and Ceratophyllales in the phylogenomics analysis. Unfortunately, in this report, we used the nuclear genome to construct the phylogenetic tree, but there has no published genomes for all these three orders. Then we attempted to use transcriptome datasets despite only a few accesses are available on NCBI. Less and poor-quality transcriptome data resulted in erroneous phylogenetic placements for some species when we performed same analysis (as shown below). Therefore, we did not include this analysis in the revised manuscript.

The used transcriptome datasets are list as follows:

Ceratophyllales: Ceratophyllum demersum:
https://www.ncbi.nlm.nih.gov/sra/ERX2099177%5Baccn%5D

Chloranthales: Chloranthus japonicus:
https://www.ncbi.nlm.nih.gov/sra/SRX3469562%5Baccn%5D

Canellales: Drimys winteri:
https://www.ncbi.nlm.nih.gov/sra?LinkName=biosample_sra&from_uid=7408298

Canellales: Canella winterana:
https://www.ncbi.nlm.nih.gov/sra?LinkName=biosample_sra&from_uid=7408296 and
https://www.ncbi.nlm.nih.gov/sra?LinkName=biosample_sra&from_uid=7408297

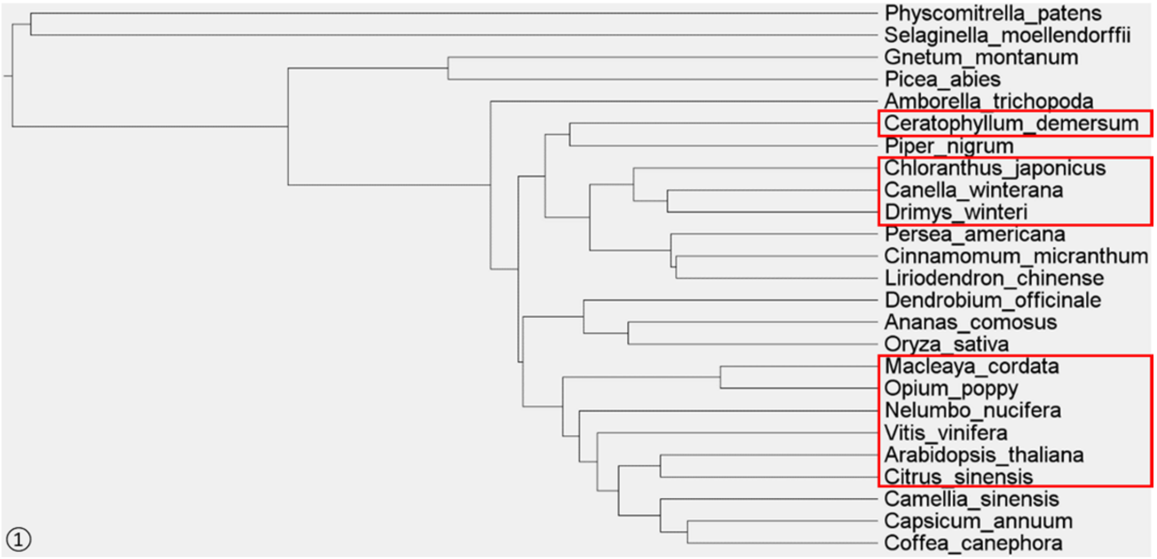

In figure 1, we used all the *Ceratophyllum demersum*, *Chloranthus japonicus*, *Drimys winteri* and *Canella winterana* with 21 other species. The results shows a spurious position for the Ceratophyllales (should be between eudicots and monocots), Chloranthales (should be sister with magnoliids), Canellales (should be sister with Piperales) and some species in eudicots compared to APG IV[1].

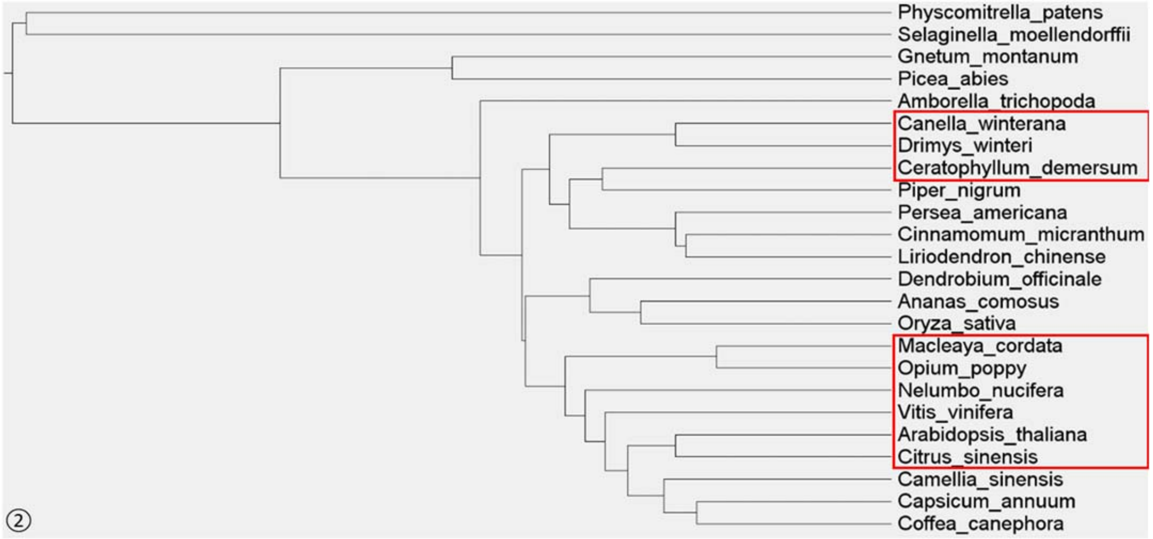

In figure 2, we used *Ceratophyllum demersum*, *Drimys winteri* and *Canella winterana* with 21 other species. The results shows similar conflicts with relationships supported by recent literature and APG IV[1].

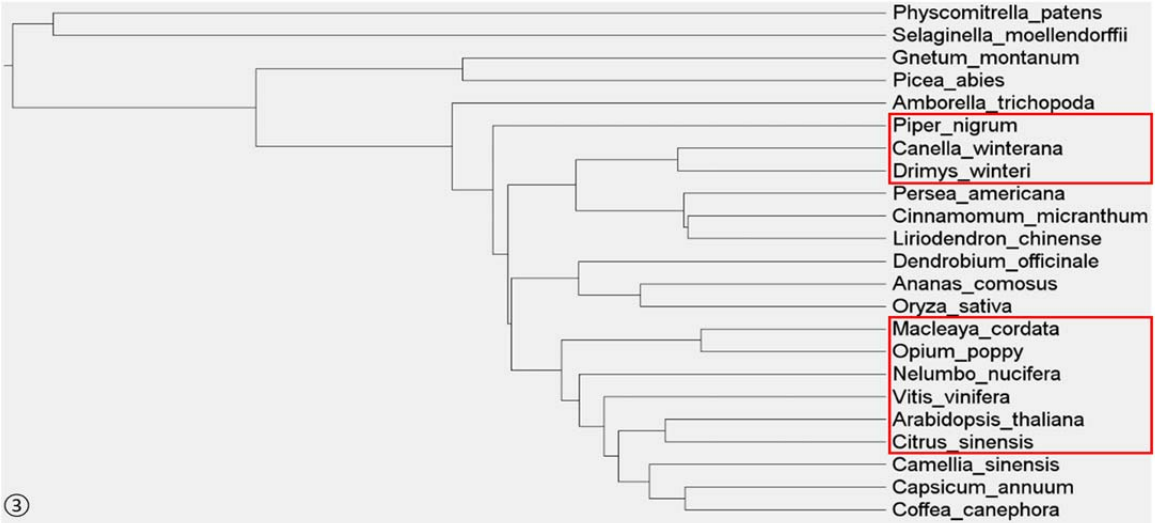

In figure 3, we only used the *Drimys winteri* and *Canella winterana* with 21 other species. The results show an erroroneous position for the Piperales (should be sister with Canellales) and some species in eudicots compare to APG IV[1].

Although this biological problem is important, the lack of high quality reference nuclear genomes for these long-isolated and ancient lineages prevents a current robust phylogenomics analysis. To summarize, we think the phylogenetic relationship was relatively accurate under the current dataset, but the formulation has been modified appropriately. By the way, we have another project for the de novo assembly of representativw species in the Canellales, to study the phylogenetic relationships amongst the magnoliids relative to eudicots and monocots.

Question: Several studies have already employed numerous nuclear and plastid genes and numerous taxa in much better phylogenetic studies of angiosperms. Some have also shown that magnoliids are well supported and sister to monocots + eudicots. Thus, the results here support a lot of previous literature and that should be noted. But again, no hard conclusions can be made here with so few taxa. Again, see the Soltis and Soltis review for discussion of these concerns.

Response: Thanks. As noted above, high quality reference nuclear genomes are absent currently for the phylogenetic study. At the same time, we revised the formulation and our results are consistent with Plastid Phylogenomic Angiosperm (PPA)[2] and APG IV[1]. The major purpose for our project is to provide a high-quality reference genome of black pepper for the functional genomics research such as sgRNA design of CRISPR-Cas9 genome editing.

Question: There are other problems with the phylogenomic portion of the ms. The authors use the term "paleoherb"; this is an old term for these plants that has inappropriate connotations and is not really used now. I would simply note that this is the first time a genome from a member of Piperales has been included.

Response: Thanks, as suggested, we have corrected this throughout the manuscript.

Question: Piper nigrum did not diverge from Amborella trichopoda at 226 mya. The ancestors may have diverged at that time. Not the living species -see below.

The divergence time estimate portion of the study is inadequate. It is very important to give a range of values around these divergence times-not a single absolute value. These are rough estimates and must be presented as such (see below)

Given the concerns raised in Soltis and Soltis 2019 (and the papers cited there in) it is hard to justify the publication of the phylogenomic part of this study. As noted the sampling is so poor that the results cannot be given serious credence. A new phylogenomic tree is not justified every time a new genome is published.

Probably the main phylogenomic point to make if made at all is that with limited taxon sampling this is another nuclear genome level study to show the same tree revealed by plastid and some nuclear data. But then stress more crucial species must be sampled- the point made by Soltis and Soltis. This would require one or two sentences of text and then everything is placed in a supplement. The dating should either removed or done properly to provide error bars around estimates. The other option is to remove all mention of phylogeny-the authors really don't have the data to do those analyses correctly---many more taxa are needed.

Response: Thanks. As suggestion, we have removed the description of divergence about *Piper nigrum* from *Amborella trichopoda* and add a range of values around divergence times in Figure 2c. For question of limited taxon sampling, the same as we reply above.

Question: Many parts of the paper are poorly written. The paper requires careful editing

Response: Thanks for your valuable comments of this manuscript. According to your suggestion, we invited native English speakers to revise the manuscript thoroughly. What’s more, we future modified by Nature Research Editing Service (Key: E682-B615-82C8-30FF-8FEP).

Question: Could the authors do more with their genome? What about synteny between the two subgenomes? How much rearrangement has occurred post polyploidy? And could synteny to other angiosperm genomes, particularly Amborella be discussed? Is there conservation of gene order at that scale?

Response: Thanks. We agree with your suggestion that it's important to consider the synteny between subgenomes for allopolyploid species. However, we need preliminary research (especially high quality reference nuclear genomes) of ancestor species that provided the subgenome. Unfortunately, we even don't know which diploid species are two closest extant progenitors. We are identifying the subgenome of *Piper nigrum*, including the pan-genome sequence and cytological analysis of some candidate piper species. Alternatively, we performed synteny analysis for the black pepper genome to Amborella and *Cinnamomum kanehirae* (Line 195-202). Really important suggests for further research.

Question: Other comments below :

32. Piper was also considered as a model genus for studies of evolution because of strikingly diverse lineages amongst basal angiosperms Poorly worded-what is this meant to say?

Response: Thank you very much for your comment. We want to emphasize the importance of black pepper in evolutionary studies. We have modified it to "Piper was also considered as a model genus for evolutionary studies for its strikingly diverse lineages amongst basal angiosperms".

Question: 47-48. which showing an evolution association and molecular basis of species-specific piperine biosynthesis from three major metabolic pathway

Response: Thanks. We appreciate your comments very much. There are three major metabolic processes (phenylpropanoid pathway, lysine metabolism and acyl transfer). We have modified this to "three major metabolic processes".

Question: 85-86. A very important point is not just that Piper is a magnoliid but first representative of Piperales. There are four sub clades recognized as orders within the magnoliids

Response: Thanks. Yes, there is Magnoliales, Laurales, Canellales and Piperales; We have improved the manuscript with clearer and more accurate phylogenetic perspectives and terminology.

Question: 206. These plants are not dicots. That group no longer is recognized or discussed. The authors must remove that term from the text throughout and also get caught up on the angiosperm phylogenetic literature-simple summaries as in the Judd et al. and Soltis et al. 2018 text books

Response: Thanks. We appreciate the positive feedback from the reviewer. As suggested, we have modified or removed that term from the manuscript throughout.

Question: 212-216. Very poorly worded text

Response: Thanks, as suggested, we have modified the manuscript.

Question: 219 Piper nigrum did not diverge from Amborella trichopoda at 226 mya. Their ancestors may have diverged at that time. Not the living species

Response: Thanks, as suggested, we have removed this sentence.

Question: 219-222 The remaining text discussing divergences is similarly presented incorrectly for the same reason

Also it is also important to give a range of values around these divergence times. They are rough estimates and must be presented as such

Response: Thanks, as suggested, we have modified and add the range of divergence times.

Question: 552 The formed time of black pepper allotetraploid poorly written

Response: Thanks, as suggested, we have modified it to "The divergence time of black pepper was calculated".

Question: 559. For assess the evolution Poor wording

Response: Thanks, as suggested, we have modified it to "To investigate the evolution".

Question: 560-565 How many genes were used to build the trees?

Response: Thanks, a total 1,722 genes within 82 single-copy orthologs among the 21 plant species were used to build the trees.

Question: 599 positive selection and episodic selection of gene poor wording

Response: Thanks, as suggested, we have modified it to "positive selection and episodic selection sites of gene".

Question: 574 which obtained from Timetree poorly worded

Response: Thanks, as suggested, we have modified it to "which obtained using".

Question: Figures are not numbered

Response: Thanks, we were numbered the figure on file name. We thank the reviewer for this suggestion, and have now add the number in figures.

Question: Phylogeny in fig 2 c. Ranges of values for divergence times must be given. Not absolute values-see comments above

Response: Thanks, as suggested, we have modified this.

Question: Phylogeny in Fig 3-how was this rooted?

Response: Thanks, follow the advice of another reviewer, we have reconstructed the phylogenetic tree in Figure 3.

Reviewer #2 (Remarks to the Author):

Hu et al. described the high quality reference genome sequence of black pepper. By adopting multiple technology including long-read sequencing, optical mapping and chromatin interaction mapping, the authors assembled 26 pseudochromosomes with N50 scaffold length of 30 Mb. Furthermore, they cleared the taxonomic position of Piperales by comparative genomics and the molecular basis of piperine biosynthesis by comprehensive transcriptomic analyses. Overall the manuscript has fair enough merit to be published as a first high quality reference genome paper of a plant belong to Piperales.

However, for improvement the quality of the manuscript, following concerns should be considered.

Question: i) Highlighting gene family evolution across plant kingdom. The authors briefly mentioned gene copy-number evolution among plants including lower, basal, gymnosperm, and angiosperm plants. Because there are only a few of sequenced basal plants, we could poorly understand global gene evolution of the plants. If the authors could highlight features and differences for copy-number and evolution of important gene families (eg. disease-resistance genes and TFs) among the basal, gymnosperm and angiosperm plants given gene duplication history and selection pressures, it will be valuable biological resources and knowledge for the readers.

Response: Thanks. It's really a critical comment that will substantially improve our research. We performed the gene family expansion and expression analysis centered around black pepper and piperine. For species belong to monocots-eudicots clade, we were selected based on the typical secondary metabolism characteristics that they can synthesise. Based on this analysis, we want to find genes/gene family involved in secondary metabolism (especially related to alkaloid synthesis) and expansion in black pepper and specific expression in the berry. Interestingly, the black pepper specific expansion genes were significantly enriched in two main types: secondary metabolite-associated functions and disease resistance (Supplementary Fig. 18). Then we compared the copy-number and evolution of genes/gene family that related to piperine biosynthesis among the basal, gymnosperm and angiosperm plants (Fig. 4 and Supplementary Fig. 32-33).

Question: ii) Transposable elements (TEs) are one major evolutionary forces especially in plants. Like the gene family evolution study, authors need to present insights of TE evolution to help understanding of genome evolution in plant kingdom for readers. Compared to gymnosperms, angiosperms (dicot and monocot) and low plants, what are specific differences in TE repertories, expansion and insertion pattern?. LTR-retrotransposons and DNA-transposons are major TEs in plants and they could be classified as multiple subgroups. For example, LTR-gypsy family could be divided as groups like del, athila and the others considering their pol proteins. The repertories of subgroups are extremely diversified among plants. What are major elements and

differences of TEs among the plants? To address this issue, the author should perform exquisite annotation and comparison of TEs.

Response: We are grateful for this comment as it points to an important feature of this study. As suggested, we annotated the TEs for the other 17 genomes (Figure 4) as method used in black pepper, except *Gnetum montanum* and *Persea americana* because of no genome can be access. Based on this result, we performed a comparative analysis of different transposable elements (TEs) in all species, which included Gypsy and Copia subgroups in LTR retrotransposons, MITE and helitrons subgroups in DNA transposons. This resulted have add to the reviewed manuscript as an independent section titled "Transposable elements (TEs) in black pepper".

Question: iii) The authors focused on their biological study for piperine biosynthesis which is the unique feature of Piper nigrum but I think that this story is too long and verbose.

Response: Thanks. We appreciate the reviewers comment, and agree that it would be good to simplified these story. Of course, the piperine biosynthesis in *Piper nigrum* was major focus on the phenylpropanoid pathway, lysine metabolism and acyl transfer processes. Our analysis of gene family expansion and tissues expression shown that many genes were involved in these three metabolic processes. So, we arrange paragraphs and organize sentences from three parts. We are very sorry for our verbose writing and we have simplified these sentences as suggested.

Minors:

Question: Line 47: "evolution association" should be "evolutionary association"

Response: Thanks, as suggested, we have modified.

Question: Line 93: "assembled" should be "assembly"

Response: Thanks, as suggested, we have modified.

Reviewer #3 (Remarks to the Author):

Hu et al present a chromosome scale genome assembly of the Magnoliid black pepper. Black pepper is presumably an allotetraploid and much of the genome is retained in duplicate. Using comparative genomics and expression analysis, the authors identified gene family expansions that may be related to piperine biosynthesis. Although the authors present a nice resource for the community, I have major and fundamental concerns that should be addressed before this manuscript is suitable for publication.

Major concerns:

Question: 1. Line 160. The total number of genes (24,814) and lack of duplicated single copy genes (based on BUSCO) would strongly suggest black pepper is diploid. Black pepper has 50 or 65% fewer genes than quinoa or cotton so this total gene number is not in line with other tetraploids.

Lines 191-194 state that 33,206 genes (or 52.16%) are retained as duplicates, bringing the total gene model number to 63,661 (I think?) and not 24,814 as stated in the text and in Table 1. I'm not sure which of these numbers is correct, but this is important for downstream analyses.

Response: Thanks. The total number of genes should be 63,466. This has been corrected.

Question: 2. More details should be provided on the genome assembly, including the statistics for each assembly step. The authors estimated the heterozygosity of the genome was around 1.3% which should have resulted in assembly of two haplotypes by Falcon-Unzip. The total size of the bionano genome map was 1.3 Gb, or roughly 2x the haploid genome size (suggesting 2 haplotypes were assembled). It is unclear how this was integrated with the genome assembly, or how haplotypes were removed/filtered.

Response: We thank the reviewer for this comment. In the PacBio assembly step, we performed additional redundancy process. For Bionano data, we first filtered the raw molecules via molecular length, label density, molecule backbone intensity and label SNR. Then, we performed the first time De Novo assembly of filtered molecules and hybrid scaffold assembly. Here, the total genome map length is nearly 1.5G and resulted

in bigger assembled genome size. We were discussed with Bionano company to find a solution for assembly of high heterozygosity genome, but they also didn't have an effective solution.We have had several rounds of discussions with other experts in genome assembly that besides first filtered of raw molecules, we also aligned the filtered molecules to assembled genome Piper_nigrum_v1 to remove the no mapping molecules. Finally, the filtered molecules through two step were performed a second time De Novo assembly with no reference genome. As filtered as the molecules have performed, is still getting a roughly 2x the haploid genome size (1.3G genome map size). So, this is may the technical defects that current BioNano algorithms still cannot effectively overcome the challenge of complex genome assembly with higher heterozygosity and polyploidy, although there is a huge contribution in diploid species. Especially for direct label and stain (DLS) optical mapping data.   This time we have an assembled genome size (800M) nearly the survey when finished hybrid scaffold assembly.

Question: 3. The HiC based heatmaps in Supplemental Figures 7 and 8 highlight major assembly issues in this genome. This most obvious errors can be seen in supplemental Figure 8e, but mistakes in scaffold order and orientation can be found in each chromosome. These issues are based on a lack of continuous interactions between neighboring contigs/scaffolds, but strong interactions elsewhere in the chromosome. Scaffolds in Figure 8u and 8z are pretty good (with some very small orientation issues) for comparison. The authors claim these regions are classified TADs and loops, but this is not true. These issues are manifested in Figure 1 where syntenic blocks between homeologous chromosomes are highly fragmented, and gene and LTR density are erratic instead of decreasing and increasing respectively near the centromeres. Because of these clear issues, it is difficult to assess the comparative genomics aspects of this manuscript. I would suggest the authors redo the HiC analysis using a different pipeline and manually inspect the final interaction matrix in a program like JuiceBox (https://github.com/aidenlab/Juicebox/wiki/Juicebox-Assembly-Tools) to fix these errors. This will substantially improve the assembly quality and utility of this genome for the community.

Response: We really appreciate this helpful comment and agree that another pipeline is of potential importance for the presented analysis. As per your suggestion, we redid the HiC analysis using the LACHESIS and manually corrected the obviously erroneous rearrangement in JuiceBox (Please see **Scaffolding the long read and BioNano assemblies with Lachesis** in **Methods**). Based on this pipeline, the quality of assembled genome has been further improved, which is reflected in fewer scaffolds numbers (71 than to 45) and high consecutiveness. It is noteworthy that the structure and number of genes have hardly changed. Unfortunately, there is still some small fragment rearrangement in intrachromosomal because of complex genomic characteristics (a highly heterozygous polyploid genome) and lack of subgenome information. Based on this chromosome-scale reference genome, we will do more in functional genomics and improve the assembly quality in subsequent studies. Once again, special thanks to you for your good comments.

Question: 4. The venn diagram of shared and unique gene families in Figure 2a contains an unusual assortment of unrelated specie making it hard to interpret. It would be better to include the basal angiosperm Amborella and the two other magnoolids (Cinnamomum kanehirae and Liriodendron tulipifera) at a minimum.

Response: We thank the reviewer for pointing out the necessity to include these data in the Figure 2a. As suggestion, we have to redo this venn diagram in Figure 2a that included *Amborella trichopoda*, *Cinnamomum kanehirae*, *Liriodendron chinense*, *Persea americana* and *Arabidopsis thaliana*.

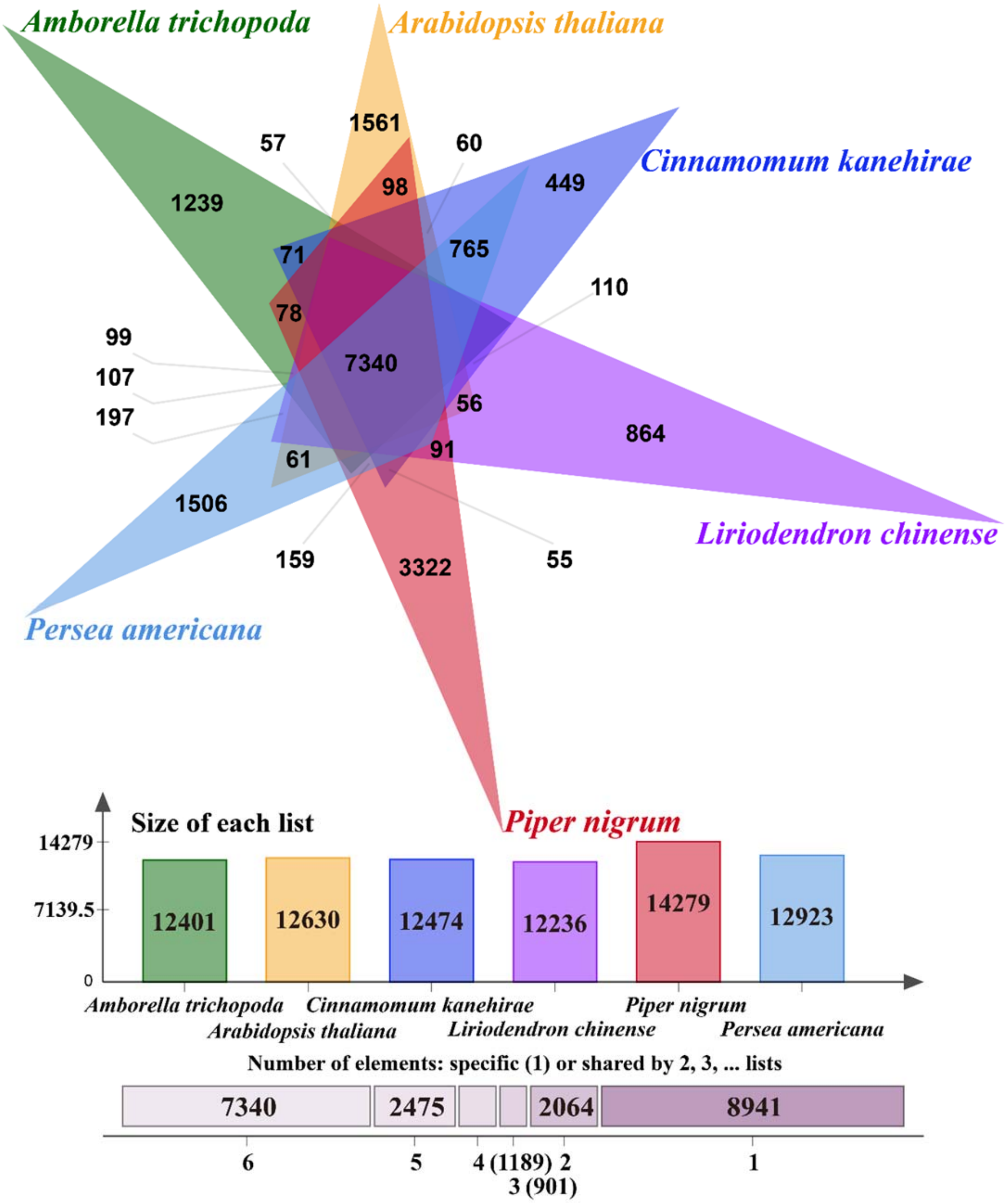

Question: 5. The phylogeny in Figure 3 is wrong, making it difficult to interpret gene family gain/loss between lineages. For instance, Arabidopsis and Carica are both Brassicales (and should be sister here) whereas Malus is a Rosales and Citrus is in the order Sapindales. These fundamental issues make it difficult to interpret these results.

Response: Thanks. We thank the reviewer for their detailed consideration of the phylogeny in Figure 3. As suggestion, we have redo this phylogenetic tree in Figure 3 (Please see as follows).

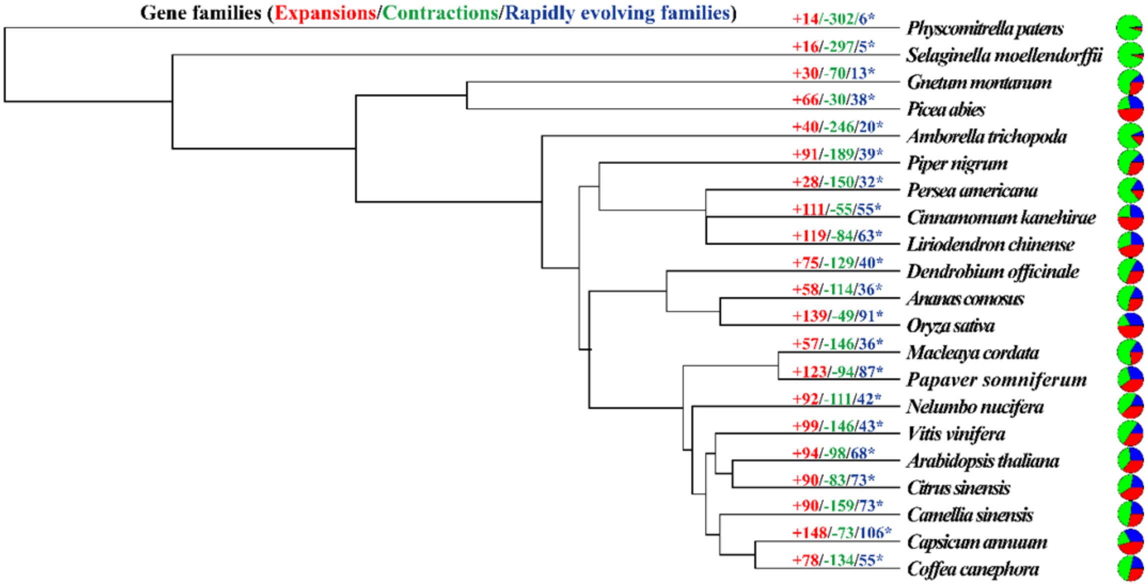

Question: 6. The two other Magnoolid genomes should be included in Figure 4a for gene family enrichment analysis. Figure 4a and b are difficult to interpret and it is hard to see if the expanded gene families have higher expression (indicating they are involved in unique processes in black pepper). I would suggest the authors use different colors and a scale in Figure 4b. The CYP90 clan is expanded in black pepper, but the expression of only one gene is shown in Figure 4b, again making it difficult to judge how meaningful these results are. It would be interesting to see if homologous genes are contributing to piperine biosynthesis differently.

Response: We thank the reviewer for this useful comment and have added other Magnoliid genomes in Figure 4a and use different colors and a scale in Figure 4b, which gives greater confidence in interpret the expanded gene families have higher expression in the berry. Following your suggestion, we have redone the gene family expansion analysis used the species in comparative genomics for phylogenomic analysis process, which include *Cinnamomum kanehirae*, *Liriodendron chinense* and *Persea americana* in the magnoliids. We also used different colors and performed Z-Score scale in both Figure 4a and 4b. Besides, in Figure 4b we only selected genes that have a berry-specific upregulated expression for each expanded gene family.

1 Group, T. A. P. *et al.* An update of the Angiosperm Phylogeny Group classification for the orders and families of flowering plants: APG IV. *Botanical Journal of the Linnean Society* **181**, 1-20 (2016).

2 Li, H.-T. *et al.* Origin of angiosperms and the puzzle of the Jurassic gap. *Nature plants*, 1 (2019).

Reviewers' comments:

Reviewer #1 (Remarks to the Author):

The manuscript is improved but still has issues (below). The strong part is the chemistry, but other areas need revision. The goals and work done in the polyploidy section are unclear. The TE section in particular seems to add little if anything of significance as written. The phylogenetic section is better but still overstates the significance of these results and also should be revised.

Quite a few corrections to English writing. A number of awkwardly worded sentences. The paper requires additional careful editing.

Pg 4. The authors do not "resolve the position" of magnoliids as claimed. They provide insights. They have results that agree with previous studies. But as stressed, much more sampling of species is needed and that was not done here.

Pg 8. "To assess black pepper polyploidy." Not really sure what was done here or what the authors had as a goal. The comparisons to coffee and sunflower and Vitis are puzzling. What was compared? Timing of WGD? This is a problematic section. What was the question? What was done? What was found?

Pg 10. Transposable elements
Does this section really add anything substantial? A number of comparisons are made that show variation across species and clades. But are there any clear trends or an important take home message? This is a not a particularly informative section. Either provide a clear take home message or remove.

Reviewer #2 (Remarks to the Author):

All the concerns that I raised in the first review process were resolved in the revised manuscript. I agree to accept the manuscript for publication.

Reviewer #3 (Remarks to the Author):

The authors have addressed my previous concerns in their revision.

We appreciate you for spending time to review our paper and providing very valuable comments. It is your valuable and insightful comments that led to possible improvements in the current version. The authors have carefully considered the comments and tried our best efforts to address every one of them. The main corrections are included in the revised manuscript and the response to the reviewers' comments are as follows:

Reviewers' comments and our response:

Reviewer #1 (Remarks to the Author):

The manuscript is improved but still has issues (below). The strong part is the chemistry, but other areas need revision. The goals and work done in the polyploidy section are unclear. The TE section in particular seems to add little if anything of significance as written. The phylogenetic section is better but still overstates the significance of these results and also should be revised.

Question:

Quite a few corrections to English writing. A number of awkwardly worded sentences. The paper requires additional careful editing.

Response: We thank the reviewer for this comment. According to your suggestion, We reorganize language and more rigorous expressions, especially for comparative genome and phylogenetic sections. Furthermore, we have sent this manuscript to Nature Research Editing Service (Key: E682-B615-82C8-30FF-8FEP) and invited two native English speakers (Jonathan F. Wendel from Organismal Biology Iowa State University Ames and Keith Lindsey from Durham University) for polishing the language, included the grammar modification and optimization of language representation.

Question:

Pg 4. The authors do not "resolve the position" of magnoliids as claimed. They provide insights. They have results that agree with previous studies. But as stressed, much more sampling of species is needed and that was not done here.

Response: We thank the reviewer for pointing out this expression that maybe not be precise. We have changed “resolve” to “gain insight into” in the sentence with red font on page 4.

In this report, we used the nuclear genomes to construct the phylogenetic tree, we couldn’t add the sampling of species as you had mentioned for the following two major reasons:

(1) Based on the uniqueness of black pepper in phylogeny, we have selected representational monocots, eudicots, basal angiosperms, gymnosperms and lower plants to build a phylogenetic tree for black pepper. We think the phylogenetic relationship of black pepper (basal angiosperms) is relatively accurate under the current dataset.

(2) There has no published genomes for all these Canellales, Chloranthales and Ceratophyllales, only a few early transcriptome datasets are available on NCBI. Less and poor-quality transcriptome data resulted in erroneous phylogenetic placements for some species when we performed same analysis (as shown below).

We sincerely hope that you can understand and accept our response.

The used transcriptome datasets are list as follows:

Ceratophyllales: Ceratophyllum demersum:
https://www.ncbi.nlm.nih.gov/sra/ERX2099177%5Baccn%5D

Chloranthales: Chloranthus japonicus:
https://www.ncbi.nlm.nih.gov/sra/SRX3469562%5Baccn%5D

Canellales: Drimys winteri:
https://www.ncbi.nlm.nih.gov/sra?LinkName=biosample_sra&from_uid=7408298

Canellales: Canella winterana:
https://www.ncbi.nlm.nih.gov/sra?LinkName=biosample_sra&from_uid=7408296 and

https://www.ncbi.nlm.nih.gov/sra?LinkName=biosample_sra&from_uid=7408297

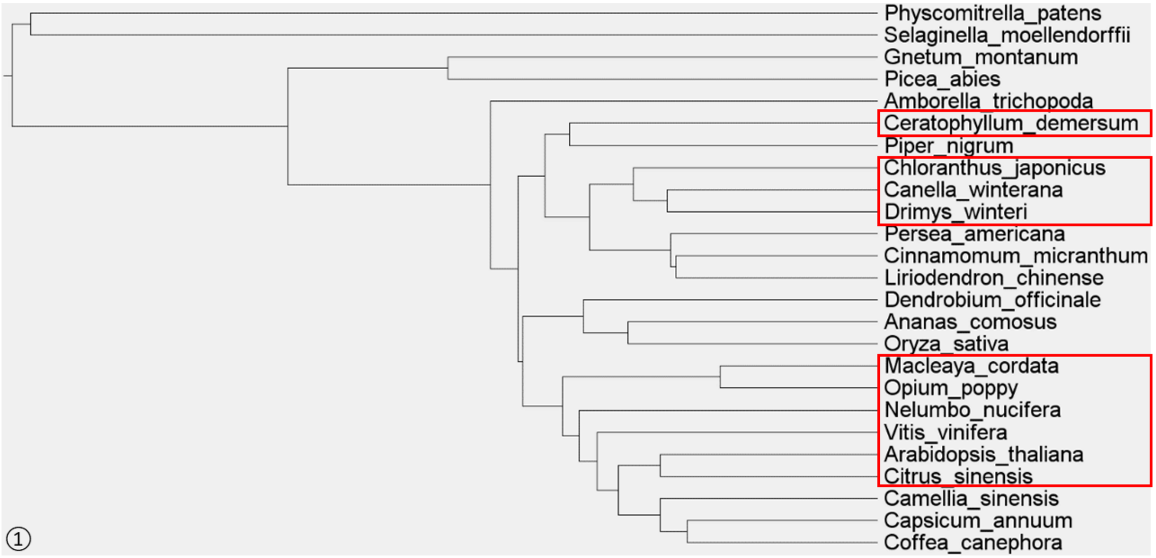

As shown in figure 1, we used all the *Ceratophyllum demersum*, *Chloranthus japonicus*, *Drimys winteri* and *Canella winterana* with 21 other species to construct the phylogenetic tree. The comparative genomics analysis only identified 24 single-copy orthologous gene families and phylogenomics analysis showed a spurious position for the Ceratophyllales (should be between eudicots and monocots), Chloranthales (should be sister with magnoliids), Canellales (should be sister with Piperales) and some species in eudicots compared to APG IV[1].

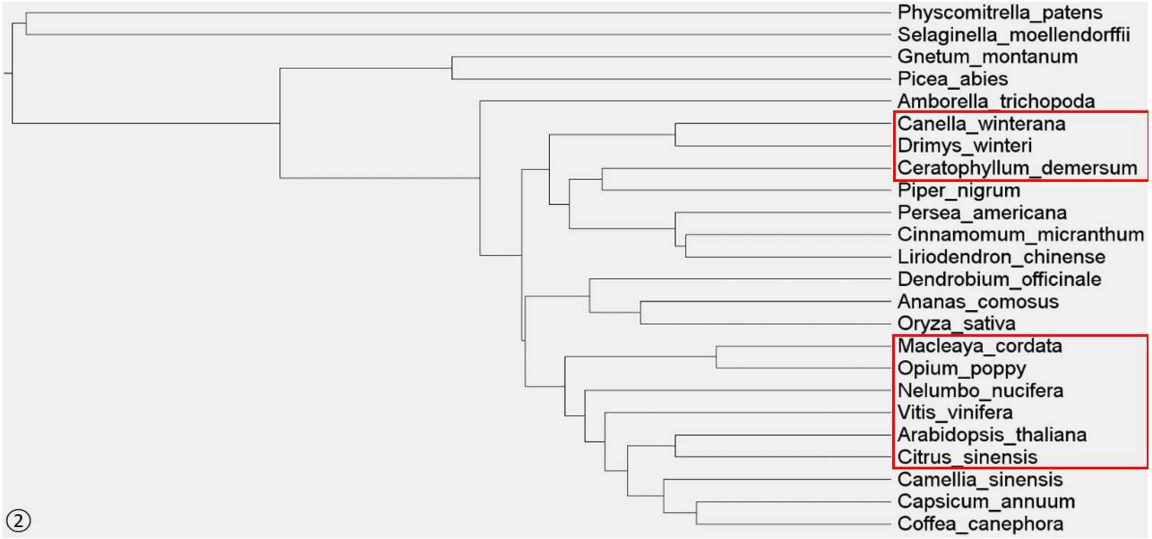

In figure 2, when we used *Ceratophyllum demersum*, *Drimys winteri* and *Canella winterana* with 21 other species, the results contained 51 single-copy orthologous gene families and showed similar conflicts with relationships supported by recent literature and APG IV[1].

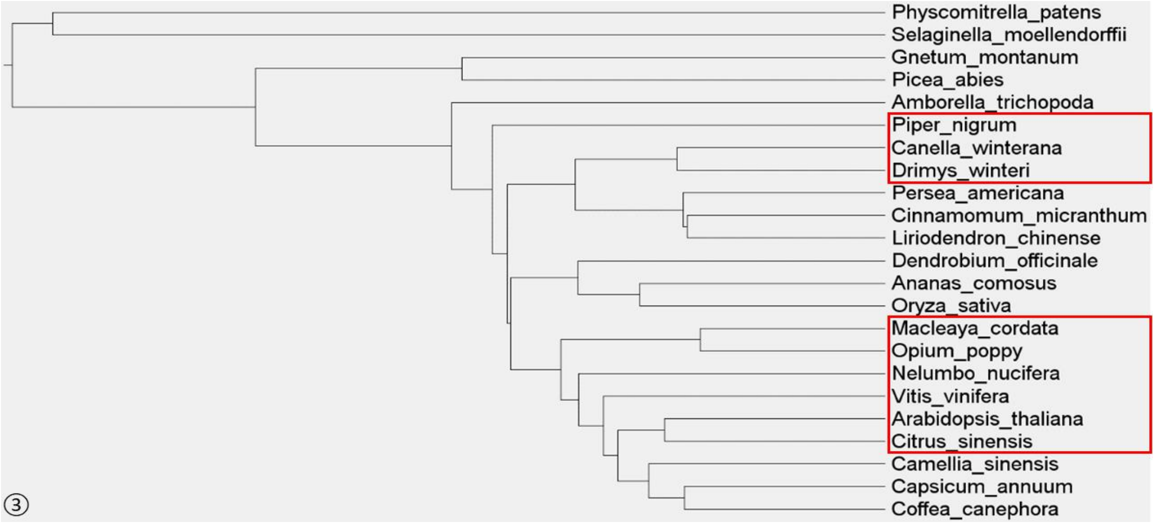

In figure 3, we only used the *Drimys winteri* and *Canella winterana* with 21 other species. The results have 54 single-copy orthologous gene families and also exhibit an erroneous position for the Piperales (should be sister with Canellales) and some species in eudicots compare to APG IV[1]. For these reason, we did not include more genomes to construct the phylogenetic tree.

Question:

Pg 8. "To assess black pepper polyploidy." Not really sure what was done here or what the authors had as a goal. The comparisons to coffee and sunflower and Vitis are puzzling. What was compared? Timing of WGD? This is a problematic section. What was the question? What was done? What was found?

Response: Thanks. It's really a critical comment that will substantially improve our research. Ancient whole-genome duplication (WGD) (also known as polyploidization) events are an important driving force of the evolution of animals, fungi and other organisms, especially plant lineages[2-5]. We want to investigate whether and when such an event happened in the black pepper's evolution history. So as suggestion in sunflower

genome evolution analysis[6], we carefully chose *Liriodendron chinense*, *Coffea canephora*, *Helianthus annuus* and *Vitis vinifera*, with special evolutionary attributes of each species, to identify the orthologs and paralogs genes, and check the *Ks* distributions. Our study revealed that both the RBH and syntenic block gene pair *Ks* distribution provided convincing evidence for a WGD event during black pepper genome evolution.

More importantly, the WGD is regarded as the basic process of species evolution. With the occurrence of WGD event, many multiple copies of genes have been generated, followed by gene silencing, disappearance and functional differentiation. Finally, WGD also strongly promotes the formation of new traits of species[7,8]. In this report, gene family analysis revealed the significant expansion of genes related to piperine biosynthesis (Fig. 4).

Question:

Pg 10. Transposable elements

Does this section really add anything substantial? A number of comparisons are made that show variation across species and clades. But are there any clear trends or an important take home message? This is a not a particularly informative section. Either provide a clear take home message or remove.

Response: Thanks for the good evaluation and kind suggestion. The transposable elements (TEs) play an important role in plant genome evolution[9-11]. Based on the uniqueness of black pepper in phylogeny, our original intention is to perform a comprehensive comparison of TE repertories in all species (used in phylogenomics analysis in this manuscript) and explore potential trends. However, too much species that span different order, to the point that no obvious trend was identified. As suggestion, we have remove this "Transposable elements (TEs) in black pepper" section into Supplementary as a note for TEs analysis of black pepper genome.

1 Group, T. A. P. *et al.* An update of the Angiosperm Phylogeny Group classification for the orders and families of flowering plants: APG IV. *Botanical Journal of the Linnean Society* **181**, 1-20 (2016).

2 Cui, L. *et al.* Widespread genome duplications throughout the history of flowering plants. *Genome Research* **16**, 738 (2006).

3 Adams, K. L. & Wendel, J. F. Polyploidy and genome evolution in plants. *Current Opinion in Plant Biology* **8**, 135-141 (2005).

4 Adams, K. Genomic clues to the ancestral flowering plant. *Science* **342**, 1456-1457 (2013).

5 Jiao, Y. *et al.* Ancestral polyploidy in seed plants and angiosperms. *Nature* **473**, 97-100 (2011).

6 Badouin, H. *et al.* The sunflower genome provides insights into oil metabolism, flowering and Asterid evolution. *Nature* **546**, 148 (2017).

7 Freeling, M. Bias in Plant Gene Content Following Different Sorts of Duplication: Tandem, Whole-Genome, Segmental, or by Transposition. *Annual Review of Plant Biology* **60**, 433-453 (2009).

8 Eric Schranz, M., Mohammadin, S. & Edger, P. P. Ancient whole genome duplications, novelty and diversification: the WGD Radiation Lag-Time Model. *Current Opinion in Plant Biology* **15**, 147-153 (2012).

9 Fedoroff, N. Transposons and genome evolution in plants. *Proceedings of the National Academy of Sciences* **97**, 7002-7007 (2000).

10 Lisch, D. How important are transposons for plant evolution? *Nature Reviews Genetics* **14**, 49 (2012).

11 Fedoroff, N. V. Transposable Elements, Epigenetics, and Genome Evolution. *Science* **338**, 758-767 (2012).

REVIEWERS' COMMENTS:

Reviewer #1 (Remarks to the Author):

The authors have addressed most of the remaining concerns. I have a number of edits to the English writing as well as issues that remain regarding the evolutionary interpretations

201 -203 Poorly worded. 51.3% of the genes were classified as being the result of either WGD or segmental duplication…

Also-how many genes are only the result of WGD-that is what is important here

214. 16.6 mya they should provide a range of estimates. Dating events requires that the uncertainty be recognized and a range of ages given. The event clearly did not occur precisely 16.6 mya

227 positions should be position

227 This is incorrect as written Piper nigrum did not diverge then….but the ancestor of Piperales diverged at that time from Magnoliales + Laurales.

260 in gene clusters

358 change to "within the magnoliid clade have remained unclear.."

We really appreciate reviewers and editors for all thoughtful comments and constructive suggestions to improve the manuscript. Therefore, we tried our best efforts to address all the concerns in this last version. In this version, we also have modified the format as editorial requests. All the major corrections are indicated by Word Tracking system in the revised manuscript and our response to the reviewers' comments are as follows:

Reviewers' comments and our response:

Reviewer #1 (Remarks to the Author):

The authors have addressed most of the remaining concerns. I have a number of edits to the English writing as well as issues that remain regarding the evolutionary interpretations

Question:

201 -203 Poorly worded. 51.3% of the genes were classified as being the result of either WGD or segmental duplication…

Also-how many genes are only the result of WGD-that is what is important here

Response: We thank the reviewer for this comment. We have modified this sentence to 'In addition, analysis of duplication types of the black pepper paralogs by MCScanX indicate that most genes were classified as WGD or segmental duplication (32,547 genes and accounting for 51.3%), followed by three other types: dispersed (19.1%), proximal (7.4%) and tandem (3.6%).' in this reviewed manuscript.

Question:

214. 16.6 mya they should provide a range of estimates. Dating events requires that the uncertainty be recognized and a range of ages given. The event clearly did not occur precisely 16.6 mya

Response: We totally agree this comments. As suggested, this estimates time has been modified to add a range of ages in the new version.

Question:

227 positions should be position

Response: Thanks, this typo has been corrected in the new version.

Question:

227 This is incorrect as written Piper nigrum did not diverge then….but the ancestor of Piperales diverged at that time from Magnoliales + Laurales.

Response: We agree with this point and modified this sentence to ‘Our results further suggest that Piperales, representative by *Piper nigrum*, first diverged from the Magnoliales (*Liriodendron chinense*) plus Laurales (*Cinnamomum kanehirae* and *Persea americana*) approximately 175-187 MYA.’ in the new version.

Question:

260 in gene clusters

Response: Thanks, following the suggestion, this has been corrected in the new version.

Question:

358 change to "within the magnoliid clade have remained unclear.."

Response: Thanks, as suggested, we revised it in the new version.
